# Comparative Proteomic and Physiological Analyses of Two Divergent Maize Inbred Lines Provide More Insights into Drought-Stress Tolerance Mechanisms

**DOI:** 10.3390/ijms19103225

**Published:** 2018-10-18

**Authors:** Tinashe Zenda, Songtao Liu, Xuan Wang, Hongyu Jin, Guo Liu, Huijun Duan

**Affiliations:** 1 Department of Crop Genetics and Breeding, College of Agronomy, Hebei Agricultural University, Baoding 071001, China; tzenda@hebau.edu.cn (T.Z.); m15028293845@163.com (S.L.); 15733289921@163.com (X.W.); m15633790536@163.com (H.J.); m15612245597@163.com (G.L.); 2 North China Key Laboratory for Crop Germplasm Resources of the Education Ministry, Hebei Agricultural University, Baoding 071001, China

**Keywords:** proteome profiling, iTRAQ, differentially abundant proteins (DAPs), drought stress, physiological responses, *Zea mays* L.

## Abstract

Drought stress is the major abiotic factor threatening maize (*Zea mays* L.) yield globally. Therefore, revealing the molecular mechanisms fundamental to drought tolerance in maize becomes imperative. Herein, we conducted a comprehensive comparative analysis of two maize inbred lines contrasting in drought stress tolerance based on their physiological and proteomic responses at the seedling stage. Our observations showed that divergent stress tolerance mechanisms exist between the two inbred-lines at physiological and proteomic levels, with YE8112 being comparatively more tolerant than MO17 owing to its maintenance of higher relative leaf water and proline contents, greater increase in peroxidase (POD) activity, along with decreased level of lipid peroxidation under stressed conditions. Using an iTRAQ (isobaric tags for relative and absolute quantification)-based method, we identified a total of 721 differentially abundant proteins (DAPs). Amongst these, we fished out five essential sets of drought responsive DAPs, including 13 DAPs specific to YE8112, 107 specific DAPs shared between drought-sensitive and drought-tolerant lines after drought treatment (SD_TD), three DAPs of YE8112 also regulated in SD_TD, 84 DAPs unique to MO17, and five overlapping DAPs between the two inbred lines. The most significantly enriched DAPs in YE8112 were associated with the photosynthesis antenna proteins pathway, whilst those in MO17 were related to C5-branched dibasic acid metabolism and RNA transport pathways. The changes in protein abundance were consistent with the observed physiological characterizations of the two inbred lines. Further, quantitative real-time polymerase chain reaction (qRT-PCR) analysis results confirmed the iTRAQ sequencing data. The higher drought tolerance of YE8112 was attributed to: activation of photosynthesis proteins involved in balancing light capture and utilization; enhanced lipid-metabolism; development of abiotic and biotic cross-tolerance mechanisms; increased cellular detoxification capacity; activation of chaperones that stabilize other proteins against drought-induced denaturation; and reduced synthesis of redundant proteins to help save energy to battle drought stress. These findings provide further insights into the molecular signatures underpinning maize drought stress tolerance.

## 1. Introduction

Maize (*Zea mays* L.) is one of the world’s most agro-economically important crops because of its raw material use in the food, feed, and biofuel production for humans and animals [1,2,3]. However, it is under severe threat from various abiotic stresses including drought, salinity, cold, heat, and flooding [4,5,6,7,8]. Among these, drought or moisture deficit is the most serious environmental factor posing a substantial menace to maize production worldwide, especially under rain-fed conditions [9,10,11].

The crop is susceptible to drought at various growth stages, including seedling, pre-flowering and grain-filling [4]. In particular, drought stress can affect plant growth at the seedling stage [12]. In arid and semi-arid regions such as Hebei Province in Northern China, maize often undergo drought stress in spring and early summer when water deficits threaten germination and seedling growth [3,13]. Although maize seedlings require less water compared to later vegetative and reproductive stages, moisture stress at seedling stage influences their adaptation at the early crop establishment phase and their grain yield potential, due to premature flowering and a longer anthesis-silk interval [14,15]. Revealing the mechanism of maize drought response at the seedling stage and improving early crop establishment in regions where drought occurs during the early crop development phase therefore become priority goals of the maize drought-tolerant breeding program [3].

Scientific research has made tremendous progress in unravelling maize drought stress response mechanisms at the vegetative and reproductive stages [16]. Despite this, however, and the existence of several reports on drought tolerance analyses between inbred lines at the seedling stage [4,17,18], our understanding of seedling drought stress response mechanisms and genes involved still remain unclear. Several reports have focused on physiological and biochemical [19,20,21], as well as large-scale transcriptomic analyses [1,3,22,23,24]. However, transcriptome profiling has limitations because mRNA levels are not always correlated to those of corresponding proteins due to post-transcriptional and post-translational modifications [5,25,26].

Elucidating the molecular changes at protein level has become extremely important for studying drought stress responses in plants. Since proteins are directly involved in plant stress responses, proteomic studies can eventually contribute to dissecting the possible relationships between protein changes and plant stress tolerance [27,28]. This, therefore, provides new insights into plant responses to drought stress at the protein level [10,29,30]. High-throughput proteomics has become a powerful tool for performing large-scale studies and comprehensive identification of drought responsive proteins in plants [31,32,33,34,35]. The iTRAQ (isobaric tags for relative and absolute quantification) analysis method is a second generation proteomic technique that provides a gel-free shortgun quantitative analysis. It utilizes isobaric reagents to label tryptic peptides and monitor relative changes in protein and PMT (peptide mass tolerance) abundance, and it allows for up to eight samples [36]. Thus, the method especially facilitates the analysis of time courses of plant stress responses or biological replicates in a single experiment, and the technique has become increasingly popular in plant stress response studies [37].

Here, in order to study maize drought stress responses at the protein level, we have also employed an iTRAQ-based quantitative strategy to perform proteome profiling of two contrasting maize inbred lines (drought-tolerant YE8112 and drought-sensitive MO17) at the seedling stage. We conducted a comparative proteomic analysis of these two lines′ leaves after a seven-day moisture-deficit exposure period. In addition, we evaluated some physiological responses of these two inbred lines under drought stress, and the results of this study provide further insights into the drought stress tolerance signatures in maize.

## 2. Results

### 2.1. Phenotypic and Physiological Differences between YE8112 and MO17 in Response to Drought Stress

**Figure 1:**
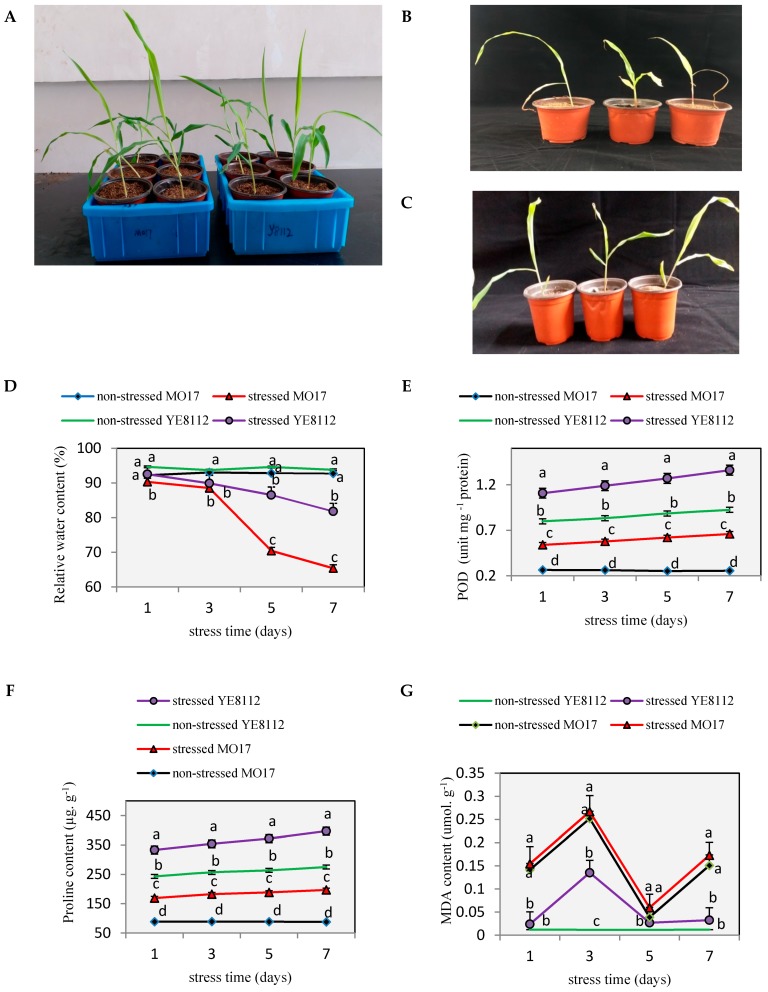
Phenotypic (**A**–**C**) and physiological (**D**–**G**) responses of two maize inbred lines to drought stress. Phenotypic displays presented here are for three-leaf-stage seedlings after 7 days of moisture deficit treatment. (**A**) MO17 and YE8112 inbred lines under non-stressed (water-sufficient) conditions; (**B**) sensitive line MO17 drought stressed; (**C**) tolerant line YE8112 drought stressed; (**D**–**G**) physiological changes were measured in leaf tissues at different stress exposure periods/time points (1, 3, 5, and 7 days); (**D**) leaf relative water content, (**E**) peroxidase (POD) enzyme activity, (**F**) proline content and (**G**) level of lipid peroxidation (MDA (malondialdehyde) content). Data are presented as the mean ± standard error (*n* = 3). Different letters above line graphs show significant difference among treatments at a given day of treatment (*p* ≤ 0.05).

To validate the previous observations that MO17 is drought-sensitive [38] and YE8112 drought tolerant [39] and to investigate the molecular mechanisms underlying YE8112 drought tolerance, seedlings at the three-leaf stage were treated with or without moisture deficit stress for 7 days in a greenhouse environment. Several drought-induced phenotypic responses were then observed. As expected, no significant phenotypic differences were observed between the two lines under water-sufficient conditions, as they both maintained intact plant architecture (Figure 1A). However, post drought exposure; there were significant differences in the performances of the two lines. The leaves of MO17 were distinctly shriveled up (Figure 1B), whilst YE8112 seedlings displayed little phenotypic change by maintaining fully expanded green leaves and intact plant architecture (Figure 1C).

**Figure 2:**
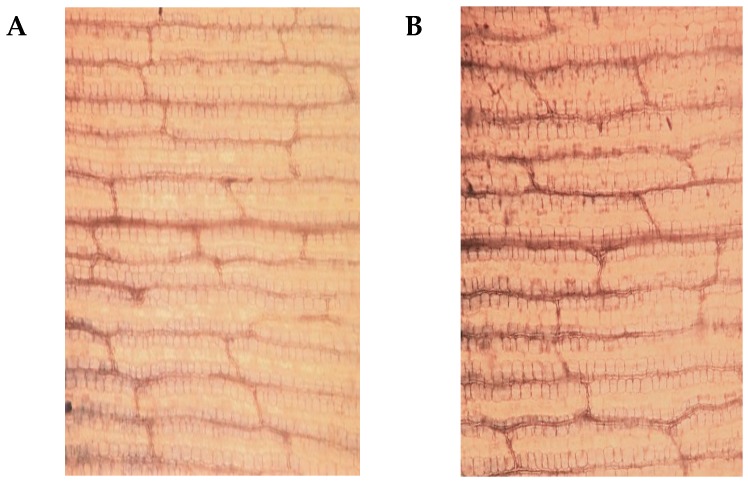

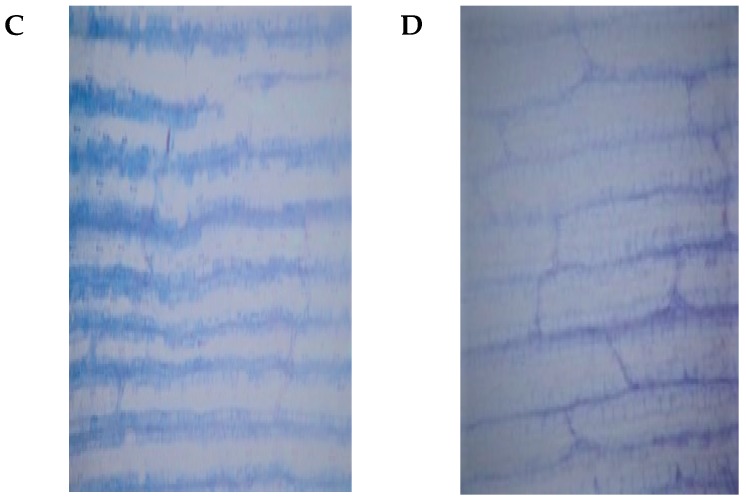
Results of trypan blue staining of leaves. (**A**) Non-stressed sensitive inbred line MO17, (**B**) non-stressed tolerant line YE8112, (**C**) drought-stressed MO17, and (**D**) drought stressed YE8112, seven days post drought exposure. Scale bars = 200 μm.

Drought stress significantly (*p* < 0.05) decreased the leaf relative water content (RWC) from day 1 in MO17, and from day 3 in YE8112 (Figure 1D). This shows that, upon exposure to drought stress, the sensitive line MO17 lost leaf water significantly quicker than tolerant line YE8112. Moreover, the RWC of YE8112 was higher than that of MO17 in water-deficit conditions (Figure 1D); these results corresponding to our visual observation. Further, the RWC change in the sensitive line MO17 was evidently higher than that of the tolerant line (Figure 1D), which indicates that the tolerant line YE8112 had higher water retention capacity than sensitive line MO17. The POD activity showed an increasing trend, in pace with increasing number of treatment days (Figure 1E). This indicates that certain drought stress intensity could result in increased production and activity of antioxidant enzymes and protective osmolytes in maize seedlings leaves. The proline content was significantly (*p* < 0.05) increased in both MO17 and YE8112 upon drought stress exposure, commencing from day 1 in both inbred lines (Figure 1F). Additionally, the proline content was generally higher in YE8112 than in MO17 at most time points under stress conditions (Figure 1F). Results on leaf malondialdehyde (MDA) content showed that overall; it was significantly higher in MO17 than in YE8112 under both stressed and non-stressed conditions. In both inbred lines, MDA content showed an increasing trend, until the third day, and then declined significantly thereafter (Figure 1G). From the fifth day onwards, MDA content exhibited a gradual decline or a uniform level in MO17 and YE8112, respectively (Figure 1G). This may suggest that with the increase of stress exposure period, leaf cell membranes are severely injured, ultimately leading to membrane lipid release and destruction of membrane structures. Trypan blue staining results indicated that under control conditions, leaf cells of both inbred lines remained intact and viable, hence, unstained (Figure 2A,B). However, post drought exposure, sensitive line MO17 had lower active cells and cell membranes were significantly damaged (Figure 2C). In contrast, tolerant line YE8112 still had more active cells (Figure 2D).

### 2.2. Inventory of Maize Seedling Leaf Proteins Identified by iTRAQ

Using the Mascot software, 172,775 spectra were matched with known spectra, and 19,678 peptides, 12,054 unique peptides, and 3785 proteins were identified. Amongst these 3785 identified proteins (Table S1), 100 (2.65%) were <10 kDa, 3301 (87.21%) were 10–70 kDa, 259 (6.84%) were 70–100 kDa, and 125 (3.30%) were >100 kDa in weight (Figure S1A). In addition, 2084 (55.06%) proteins were detected based on at least two unique peptides whilst the remaining 1701 (44.94%) proteins had only one identified unique peptide (Figure S1B). Protein sequence coverage was generally below 25% (Figure S1C). Proteins with at least one unique peptide were used for a subsequent analysis of differentially abundant proteins (DAPs). The distribution of the peptide lengths defining each protein showed that over 85% of the peptides had lengths between 5 and 20 amino acids, with 9–11 and 11–13 amino acids being modal lengths (Figure S1D).

### 2.3. Analysis of Diffentially Abundant Proteins (DAPs) Observed in Different Experimental Comparisons

**Figure 3:**
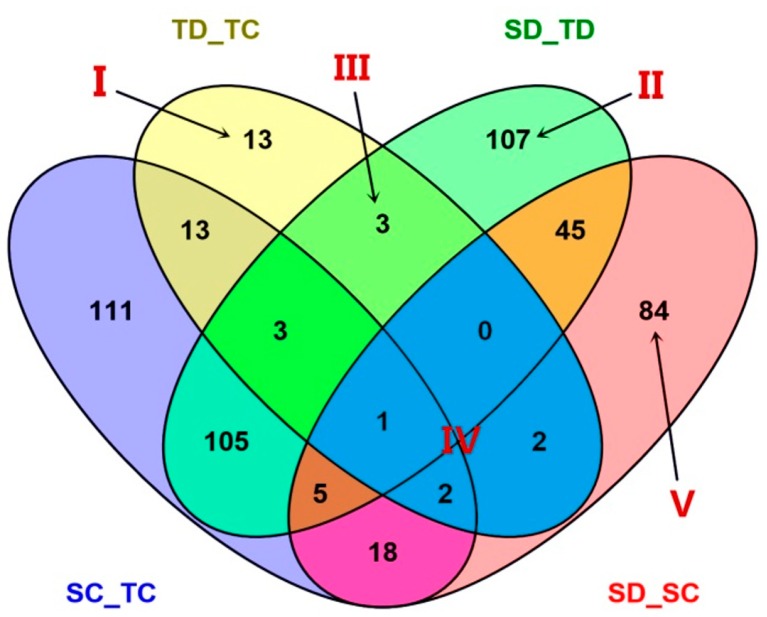
Venn diagram analysis of differentially abundant proteins (DAPs) identified in the four experimental comparisons. The overlapping regions of the Venns indicate the DAPs shared between/among corresponding groups. Area I represents 13 drought responsive DAPs specific to TD_TC; Area II represents 107 DAPs exclusive to SD_TD; Area III shows the 3 DAPs specifically shared between TD_TC and SD_TD; Area IV shows the five overlapping DAPs within line (shared between TD_TC and SD_SC); Area V shows 84 DAPs exclusive to SD_SC comparison.

Comparative proteomic analysis was used to investigate the changes of protein profiles in leaves of YE8112 (drought-tolerant, T) and MO17 (drought-sensitive, S) inbred lines under drought stress conditions. A pairwise comparison of before and after treatments (drought, D, and control, C) was performed in YE8112 (TD_TC) and MO17 (SD_SC) individually. In addition, a comparative study on the drought stress proteome was performed between the tolerant and sensitive lines, under drought (SD_TD) and under water-sufficient (control) (SC_TC) conditions, giving four comparison groups (Table 1). Before drought treatment, a total of 258 differentially abundant proteins were identified between the tolerant and sensitive lines (SC_TC). Of these DAPs, 119 had higher accumulation levels in the tolerant line compared to the sensitive line (Table 1). After drought treatment, we found 269 DAPs between the tolerant and sensitive lines (SD_TD). Of these DAPs, 116 had higher expression levels in the tolerant line compared to the sensitive line (Table 1). In the tolerant line, 37 proteins (Table S2) showed differential abundance before and after drought treatment (TD_TC); 11 of these DAPs were up-regulated (Table 1). In the sensitive line, we observed 157 DAPs (Table S3) before and after drought treatment (SD_SC); 65 of these DAPs were up-regulated whilst 92 were down-regulated (Table 1). In total, 721 DAPs were found among the four comparison groups (Table 1, Figure 3).

**Figure 4:**
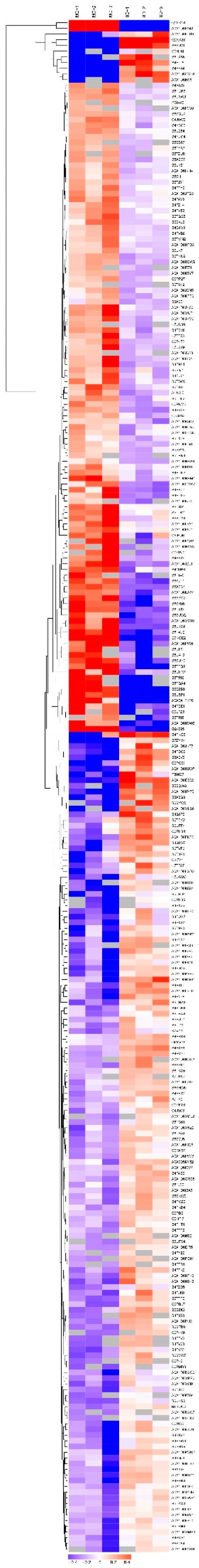
Clustering analysis of differentially abundant proteins (DAPs) in SD_TD comparison. Each row represents a protein significantly abundantly expressed. First three columns refer to technical replicates (MD1–3) for MO17 drought stressed, whilst the last three columns (8D1–3) refer to replicates for YE8112 drought stressed. The scale bar on the *X*-axis indicates the logarithmic value (log 2) expression of the DAPs, up-regulated (red) and down-regulated (blue).

With reference to Figure 3, the combinations of the four comparisons reflect the impact of lines or treatment. Some of the combinations are more important than others in respect of drought tolerance. Area I represents specific DAPs of TD_TC, that is, the specific drought responsive DAPs of the drought tolerant line YE8112. Of these 13 DAPs, five were up-regulated and eight were down-regulated (Table 2). For comparative analysis, Table 3 shows the 84 drought responsive DAPs unique to SD_SC (labeled V in Figure 3); of which 35 were up-regulated and 49 down-regulated. Area II represents specific DAPs of SD_TD, that is, specific DAPs shared between the drought sensitive and drought tolerant lines after drought treatment. For detailed analysis of these 107 specific DAPs of SD_TD, please refer to Figure 4 and Table S4.

Area III represents the three specifically shared DAPs between TD_TC and SD_TD, that is, drought responsive DAPs of the tolerant line that were also differentially expressed between the tolerant and sensitive lines after drought treatment. Of these three DAPs, all were up-regulated in the TD_TC comparison, but all down-regulated in the SD_TD comparison (Table 4). Area IV represents the five DAPs shared by TD_TC and SD_SC, that is, the common (overlapping) drought responsive DAPs within line. Of these five common drought responsive DAPs, all were down-regulated in tolerant line YE8112; whereas three were up-regulated and two down-regulated in sensitive line MO17 (Table 5).

An analysis of the log_2_ fold-changes of the significant differentially abundant proteins revealed that, in response to drought stress, DAPs in MO17 had significantly higher fold changes than DAPs in drought tolerant line YE8112 (Figure 4, Figures S2 and S3).

### 2.4. Gene Ontology (GO) Annotation and Functional Classification of the Drought Responsive DAPs

We performed gene ontology (GO) annotation to assign GO terms to the DAPs using Blast2GO web-based program (https://www.blast2go.com/). Further, GO functional classification of the GO-term-assigned-DAPs into biological processes (BP), molecular functions (MF), and cellular component (CC) categories was carried out. For the tolerant inbred line YE8112-specific DAPs (Area I of Figure 4), GO:0010196 (non-photochemical quenching), GO:1990066 (energy quenching), GO:0010155 (regulation of proton transport), GO:0009644 (response to high light intensity), and GO:0009743 (response to carbohydrates) were the most significantly enriched terms in the BP category; GO:0010333 (terpene synthase activity), GO:0003937 (IMP cyclohydrolase activity) and GO:0004126 (cytidine deaminase activity) were significant in the MF category; whereas GO:0009503 (thylakoid light-harvesting complex), GO:0030076 (light-harvesting complex), GO:0009783 (photosystem II antenna complex), GO:0098807 (chloroplast thylakoid membrane protein complex), and GO:0009517 (PSII associated light-harvesting complex II) were significant GO terms in the CC function (Table S5; Figure S4A).

In the SD_TD comparison (Area II of Figure 4), GO:0065004 (protein-DNA complex assembly), GO:0006323 (DNA packaging), GO:0006325 (chromatin organization) and GO:0006334 (nucleosome assembly) were the most significant terms in BP category; whilst GO:0046982 (protein heterodimerization activity), GO:0046983 (protein dimerization activity) and GO:0004473 (malate dehydrogenase (decarboxylating) (NADP+) activity) were the most significantly enriched under the MF category (Table S6; Figure S4B). Among the significant GO terms in the sensitive line MO17 (SC_SD) were GO:0051276 (chromosome organization), GO:0007059 (chromosome segregation) and GO:0006338 (chromatin remodeling) in the BP category; GO:0008135 (translation factor activity, RNA binding), GO:0003676 (nucleic acid binding), and GO:0003924 (GTPase activity) in the MF category; GO:0005694 (chromosome), GO:0000785 (chromatin) and GO:0044427 (chromosomal part) in the CC functions (Table S7; Figure S4C).

**Figure 5:**
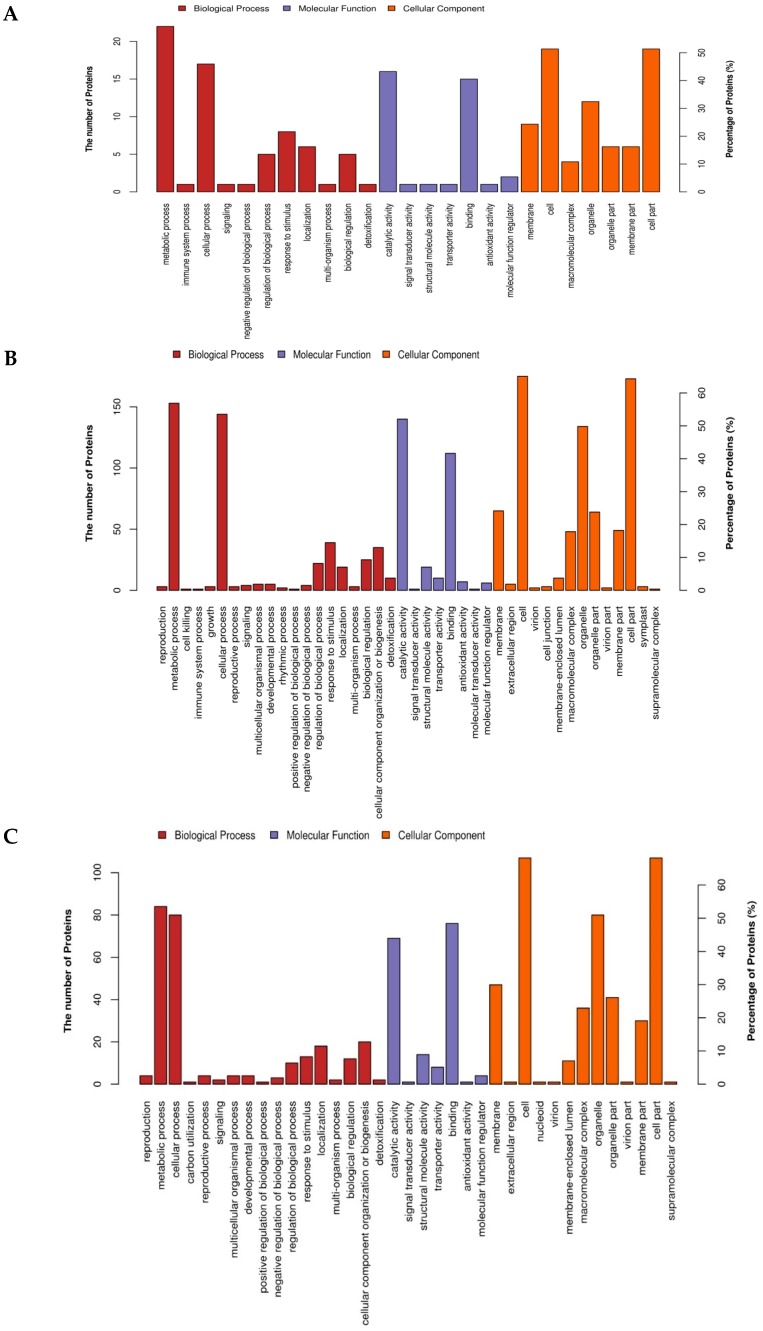
Gene ontology (GO) functional classification of drought responsive proteins. (**A**) YE8112 specific DAPs; (**B**) SD_TD specific DAPs; and (**C**) MO17 specific DAPs. *Y*-axis represents the number (and%) of proteins in each function; *X*-axis displays the protein functions, categorized into three broad biological functional groups.

The significantly enriched GO terms in each of the three comparison groups (TC_TD, SD_TD, SC_SD) were mapped to the top 20 biological functions. Among the tolerant line YE8112 (TC_TD) -specific DAPs, metabolic process (46.86%), cellular process (36.23%) and response to stimuli (7.69%) were the most popular BP functions; catalytic activity (48.0%) and binding (40.47%) most prominent in MF category; whilst cells and cell parts (47.0%), organelles (22.31%), organelle parts (5.88%), membrane (17.01%), and membrane parts (7.75%) were the popular locations for the DAPs under CC functions (Figure 5A). In the Area II (SD_TD) DAPs, metabolic process (50%), cellular process (35%), and response to stimuli (15%) in BP category; catalytic activity (55%) and binding (43%) in MF category; cell (55%), cell part (45%), and organelle (50%) in CC functional category were prominent (Figure 5B). Among the sensitive line MO17 (SD_SC)-specific DAPs, metabolic process (48.03%), cellular process (37.41%) and cellular component organization (10.22%) were the most common biological processes; catalytic activity (46.37%), binding (48.19%), and structural molecule activity (5%) in the MF category; whereas cells and cell parts (44%), organelles and organelle parts (20%) and membrane (23%) were prominent in CC functions category (Figure 5C).

### 2.5. KEGG Pathway Enrichment Analysis

**Figure 6:**
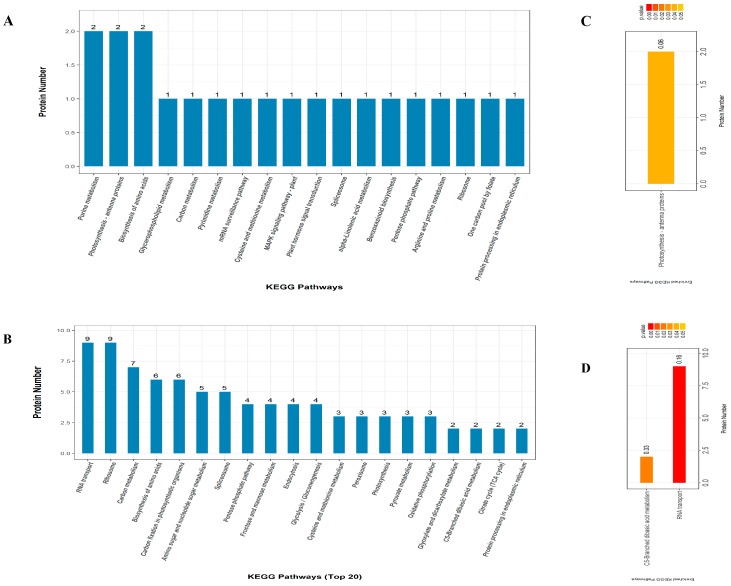
KEGG pathway enrichment analysis of the DAPs. (**A**) TD_TC comparison; (**B**) SD_SC comparison. The whole number above the bar (blue) graph represents number of DAPs enriched in the corresponding pathway. (**C**) Most significantly enriched pathway in TD_TC. (**D**) Most significantly enriched pathways in SD_SC based on the hypergeometric test. The significance of the enrichment of the KEGG path is based on the Student’s *t*-test, *p* < 0.05. The color gradient represents the size of the *p* value; the color is from orange to red, and the nearer red represents the smaller the *p* value, the higher the significant level of enrichment of the corresponding KEGG pathway. The label above the bar graph shows the enrichment factor (rich factor ≤ 1), and the enrichment factor indicates the number of differentially abundant proteins participating in a KEGG pathway as a proportion of proteins involved in the pathway in all identified proteins.

To further analyze the functional consequences of the drought-responsive DAPs, we mapped them to the Kyoto Encyclopedia of Genes and Genomes (KEGG, available online: https://www.genome.jp/kegg/; accessed on 16 March 2018) database and the DAPs were assigned to various biological pathways. Additionally, significant KEGG pathway enrichment analysis was performed using the hypergeometric test. Proline metabolism (two proteins), photosynthesis antenna proteins (2) and biosynthesis of amino acids (2) were the top three enriched pathways in YE8112 (Figure 6A). However, the composition of the enriched KEGG pathways in MO17 differed significantly, with RNA transport (nine proteins), ribosome (9), carbon metabolism (7), biosynthesis of amino acids (6), and carbon fixation in photosynthetic organisms (6) being the top most enriched pathways (Figure 6B). These results show that more proteins were observed in the enriched pathways of MO17 than YE8112 and that the two inbred lines diverge significantly in pathway responses to drought stress. Using a hypergeometric test, KEGG pathways that had a *p* value < 0.05 were considered to be significantly affected by drought stress. We observed that only one pathway (photosynthesis antenna proteins) was considerably enriched (0.06) among the YE8112 pathways (Figure 6C), whist two KEGG pathways, RNA transport (0.16) and C5-branded dibasic acid metabolism (0.33), were significantly enriched among MO17 pathways (Figure 6D).

### 2.6. Protein-Protein Interactions (PPI)

**Figure 7:**
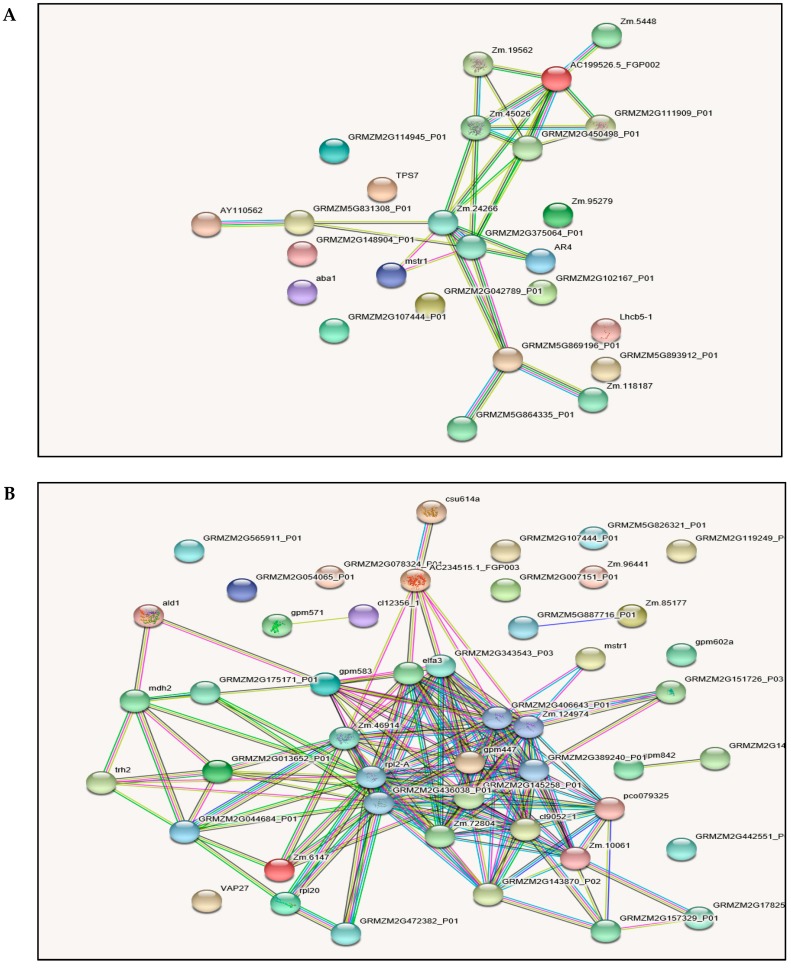
Protein interaction network consisting of DAPs identified in drought stressed maize seedling leaves of (**A**) YE8112 (**B**) MO17. The network was constructed using the String program (http:// www.string-db.org/) with a confidence score higher than 0.5. Nodes represent proteins, and the line thickness represents the strength of the supporting data.

Plant cell and tissue proteins do not act as individual molecules, but, rather, play coordinated and interlinked roles in the context of networks [30]. To determine how maize leaf cells’ drought stress signals are transmitted through protein-protein interactions to affect specific cellular functions, the identified YE8112 and MO17 DAPs were further analyzed using the String 10.5 database. Three groups of interacting proteins were identified in YE8112 (Figure 7A). The first and largest network comprise Adenosylhomocysteinase (Zm 19562); hypothetical protein LOC100194360 (AC 199526.5_FGP002); 5-methyltetrahydropteroyltriglutamate-homocysteine methyltransferase (Zm 45026); O-succinylhomoserine sulfhydrylase (GRMZM2G450498_P01); Adenosylhomocysteinase (GRMZM2G111909_P01); uncharacterized protein (Zm 24266); and Glutamate synthase 2 (NADH) (GRMZM2G375064_P01). These proteins are crucial in amino acid metabolism, maintaining antioxidant defense and epigenetic regulation (DNA methylation and histone modifications). The second group was constituted by (Zm 24266)—(GRMZM2G375064_P01)—electron transporter/thiol-disulfide exchange intermediate (GRMZM5G869196_P01) linkage. These proteins are involved in amino acid metabolism, energy metabolism (NADPH production), electron transport and stress signaling, and maintaining redox homeostasis.

The third interaction network involved (Zm 24266)—hypothetical protein LOC 100274507 (AR4)—(GRMZM2G375064_P01)—Arginase 1 mitochondrial-like (GRMZM5G831308_P01). These proteins interact in energy (NADH) production and polyamines and proline synthesis. In addition, four protein pairs (including AY110562—GRMZM5G831308_P01, Zm 5448—AC 199526.5_FGP002, GRMZM5G869196_P01—GRMZM5G864335_P01, and GRMZM5G869196_P01—Zm 118187) were observed (Figure 7A). Meanwhile, separate protein interaction networks were predicted for MO17, including a large and complex network, several small networks, and protein pairs (Figure 7B).

### 2.7. Quantitative Real-Time RT-PCR (qRT-PCR) Analysis

**Figure 8:**
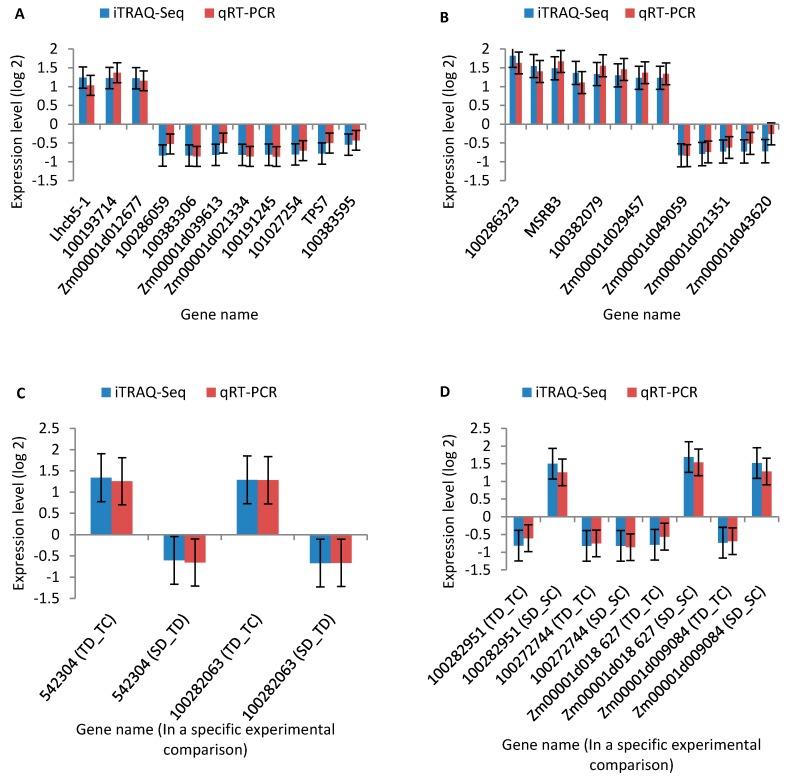
Confirmation of iTRAQ-seq results by quantitative real-time PCR (qRT-PCR). Quantitative RT-PCR analysis of the expression patterns of the maize seedling leaf genes encoded by differentially abundant proteins (DAPs) from different comparisons: (**A**) DAPs specific to TD_TC; (**B**) DAPs specific to SD_TD; (**C**) DAPs shared between TD_TC and SD_TD; and (**D**) Common DAPs shared between TD_TC and SD_SC. The *y*-axis represents qPCR relative expression levels (log_2_-fold change) and fold-change of the iTRAQ-seq data. All genes with negative values of expression level means that they were down-regulated in response to drought stress. Maize gene *GAPDH* (accession no. X07156) was used as the internal reference. Error bars represent the SE (*n* = 3).

To confirm our findings based on iTRAQ sequencing data, we conducted a supporting experiment by using quantitative real-time PCR (qRT-PCR). We made the selection of genes based on the following criteria: highly differentiated in response to drought stress and reported to be potentially associated with drought tolerance. A sample of 30 genes (Table S8) was selected from the drought responsive DAPs from different groups (labeled Areas I–IV of Figure 3). Results of the qRT-PCR analysis confirmed our findings based on iTRAQ seq data. In particular, the patterns of iTRAQ seq expression on all 30 genes were replicated by the qRT-PCR approach (Figure 8A–D; Table S9). A correlation coefficient (R^2^) (of the fold changes between qRT-PCR and iTRAQ seq) of 83.51% was obtained (Figure S5), endorsing that our iTRAQ sequencing data was reliable.

## 3. Discussion

Drought stress is the most serious environmental stress posing a severe threat to maize production worldwide [9,10,11,16,40]. In response to drought stress, plants evolve complex adaptive mechanisms at the physiological, biochemical, and molecular levels [41,42,43,44,45]. To gain in-depth understanding of the determinants underpinning drought tolerance in maize, herein, we have performed proteomic analysis of two contrasting maize inbred lines (drought-tolerant YE8112 and drought-sensitive MO17) after a seven-day moisture-deficit exposure period at the seedling stage. Further, we compared some physiological responses of these two inbred lines under drought stress conditions, and our findings provide further insights into the drought stress tolerance signatures in maize.

### 3.1. Inbred Lines YE8112 and MO17 Showed Significant Differences in Physiological Response to Drought Stress

Our experimental findings on physiological indices showed that the two maize inbred lines performed differently under drought stressed conditions. Relative water content (RWC) decreased significantly in leaf tissues of both inbred lines under drought stress conditions, and at most stress exposure time periods (days). It was generally higher in YE8112 seedlings both under non-stress and drought stress conditions (Figure 1D). We suggest that the high relative water content could help the tolerant inbred line YE8112 to perform physio-biochemical processes more efficiently under drought stress environment than the sensitive line MO17. Similarly, Moussa and Abdel-Aziz [46] observed RWC to be significantly higher in the tolerant maize genotype Giza 2 than sensitive genotype Trihybrid 321 under both control and water stress conditions.

Upon exposure to abiotic stresses, tolerant cells activate their enzymatic antioxidant system, which then starts quenching the ROS (reactive oxygen species) and protecting the cell [47]. Peroxidases (POD) and superoxide dismutase (SOD) constitute the first line of defense via detoxification of superoxide radicals, by acting as H_2_O_2_ scavenging enzymes [48]. Our investigation of the POD and proline contents of the two lines revealed that tolerant YE8112 seedlings always accumulated greater amounts of the antioxidant enzyme and protective osmolyte than sensitive MO17 seedlings under drought conditions (Figure 1E,F). The POD activity was enhanced continuously with increasing drought stress exposure period (days) in both inbred lines. However, the percent enhancement was significantly greater in tolerant line YE8112 than sensitive line MO17 (Figure 1E). It has been recognized that improved POD activity under stress conditions protects plant cells from oxidative damage emanating from reactive oxygen species (ROS) generated under such conditions [46]. In the current study, the tolerant inbred line YE8112 had greater POD activity than sensitive line MO17, which may infer better ROS quenching capacity of YE8112 than MO17. Moreover, higher proline content in YE8112 leaf tissues may explain the improved cell homeostasis in YE8112 than MO17 [49]. Higher proline content in the cells has been revealed to lower cell water potential, consequently promoting increased water absorption into those cells, thereby providing cells with immediate short-term cushion against the effects of water deficit [50].

In the present investigation, tolerant maize inbred line YE8112 maintained a higher cell membrane stability index under drought-stress conditions (Figure 2). Contrastingly, the lower membrane stability index in MO17 reflects the extent of lipid peroxidation, which in turn is a consequence of higher oxidative stress due to water stress conditions [48]. The MDA content was significantly higher in MO17 than in YE8112, both under non-stress and drought-stress conditions (Figure 1G). The rise in MDA content in both inbred lines under stress conditions suggests that drought stress could induce membrane lipid peroxidation and membrane injury by means of ROS [47,51]. In the current study, the tolerant line YE8112 had lower MDA content values than the sensitive line MO17, indicating that YE8112 cells had a better ROS quenching ability than MO17 cells, hence improved cell membrane stability. Previously, it has been revealed that higher cell membrane stability and improved cell water preservation capacity of the tolerant maize lines help them better endure moisture deficit as compared to (the low membrane stability and poor water retention capacity of) the sensitive lines [3,46]. Further, the iTRAQ analysis showed that the two genotypes’ responses to drought stress were quite different. After drought stress treatment and at the standard fold change of ≥2 and false discovery rates (FDR) <0.001, drought-tolerant YE8112 had relatively lower DAPs than drought-sensitive MO17 (Figure 4). Under drought conditions and compared to inbred line MO17, tolerant line YE8112 maintained higher leaf RWC (Figure 1D), consequently leading to relatively lower stress at the cellular level. This has been further confirmed by trypan blue staining (Figure 2). Thus, YE8112 had a more limited proteome response. A series of reports on maize seedling-stage abiotic stress analyses between different inbred lines exist [2,4]. In particular, Li et al. [2] found relatively large number of differentially expressed genes (DEGs) in freezing-sensitive inbred line Hei8834 than freezing-tolerant line KR701 after freezing treatment. Similarly, Zheng et al. [4] realized greater proportion of DEGs in drought-sensitive Ye478 than drought-tolerant Han21 after drought treatment. From the above analysis, we could confidently conclude that in our study, there was high consistence between proteome profiling data and the phenotypic and physiological characterization of the two inbred lines.

Thus, from these findings, it can be inferred that the stress tolerance mechanism exists at seedling stage of maize inbred lines. The YE8112 inbred line is comparatively tolerant to drought stress owing to its maintenance of higher RWC and proline contents under both non-stressed and stressed conditions, higher increase in POD enzyme activity, along with decreased level of lipid peroxidation (MDA content). The higher membrane stability index and high water retention capacity might have also imparted drought stress tolerance in YE8112.

### 3.2. Drought Responsive DAPs Observed in the Tolerant Inbred Line YE8112

#### 3.2.1. Photosynthesis (Photosystem II) Related Proteins Are the Major Drought Tolerance Signature in YE8112

Among the up-regulated DAPs observed in YE8112 were chlorophyll a-b binding proteins (Q41746 encoding *Lhcb5-1*; and B4FL55 encoding *542320/Lhcb5-2*). It has been noted that drought stress induced mismatch between photosynthetic light capture and utilization limits the overall plant cell photosynthetic efficiency [48]. The inhibition in photosynthesis activity results from the cell-damaging ROS that are generated in the PSII reaction center of the thylakoid membranes when cells exude excess light energy [52]. In response, plants activate the proteins involved in balancing photosynthesis light capture and utilization and non-photochemical quenching. In the current study, chlorophyll a-b binding proteins (Q41746 and B4FL55) were significantly up-regulated in response to drought stress. In addition, GO annotation analysis (See Section 2.4 above; Figure S4A) showed that under the biological process and cell component categories, the terms related to non-photochemical quenching, energy quenching, response to high light intensity, PSII antenna complex, PSII associated light harvesting complex II, and thylakoid light-harvesting complex were dominating and most significantly enriched. Furthermore, ‘photosynthesis antenna proteins’ KEGG pathway was the most significantly enriched in YE8112 (Figure 6A). Thus, these genes (*Lhcb5-1*, *542320/Lhcb5-2*) play pivotal roles in PSII associated light-harvesting complex and cysteine biosynthesis process [53,54]. This appears to be the tolerant inbred line YE8112’s major molecular signature in drought stress tolerance.

#### 3.2.2. Up-Regulation of Lipid-Metabolism Related Proteins Could Contribute to Increased Signaling and Water Conservation in the Cell

Lipid metabolism related proteins (Q2XX23, nsLTPs; A0A1D6GAZ6, GDPD) were up-regulated in response to drought stress (Table 2; Table S2). Several potential biological functions of nsLTPs have been proposed, including their (nsLTPs) involvement in long-distance signaling that possibly is implicated in plant defense against pathogens [55], and the formation of protective hydrophobic layer on the surfaces of plant aerial organs [56]. In barley (*Hordeum vulgaries* L.) and *Zea mays* L. leaves, nsLTPs, working in synergy with thionins, were identified as potent inhibitors of bacterial and fungal plant pathogens [57]. This may indicate that plants may have developed cross-tolerance mechanisms to cope with abiotic and biotic stresses [5,58]. The GDPD (Glycerophosphodiester phosphodiesterase) which hydrolyzes glycerophosphodiesters into sn-glycerol-3-phosphate (G-3-P) and the corresponding alcohols, plays a crucial role in lipid metabolism in both prokaryotes and eukaryotes [59]. Cheng et al. [60], studying on *Arabidopsis thaliana*, suggested that the GDPD-mediated lipid metabolic pathway may be involved in release of inorganic phosphate from phospholipids during phosphate starvation. Here, we also submit that the enhancement (up-regulation) of lipid-metabolism related proteins could contribute to increased signaling and water conservation in the cell through formation of hydrophobic layer on leaf surface (which enables the leaves of stressed maize to normal growth under stress), and thus, is an indispensable adaptive response to drought stress in maize seedlings.

#### 3.2.3. Enhancement of Molecular Chaperons Is a Vital Strategy for Drought Stress Tolerance in YE8112

To confront protein inactivation or denaturation resulting from drought stress, plants activate protective mechanisms that include chaperones and chaperone-like proteins, osmolytes or compatible solutes [61]. Here, abscisic acid stress ripening 1 (ASR1) protein was up-regulated in response to drought stress. Previously, the combined effort of tomato *ASR1* gene analogue (*S1ASR1*) and osmolyte glycine-betaine has been shown to stabilize other proteins against heat and cold stress induced denaturation, thereby protecting those proteins under such conditions [62]. Kalifa et al. [63] had observed that overexpression of the water and salt stress-regulated *Asr1* gene confers an increased salt tolerance. Earlier, they had concluded that steady-state cellular levels of tomato ASR1 mRNA and protein are transiently increased following exposure of plants to poly (ethylene glycol), NaCl or abscisic acid [64]. Universal stress proteins (USP) are widely spread proteins in nature, belonging to the PF00582 superfamily (COG0589) and are suggested to function in nucleotide binding and signal transduction [65]. In stress conditions such as heat shock, nutrient starvation, the presence of oxidants, DNA-damaging agents, or other stress agents which may arrest cell growth, USPs are overproduced and through a variety of mechanisms aid the organism survive such uncomfortable condition [66]. Furthermore, HSP protein (B4FVB8), alpha/beta hydrolase superfamily protein (B6T6N9) and Clp B3 protein chloroplastic (A0A1D6HFD3) were up-regulated in YE8112 (in the SD_TD comparison) in response to drought (Table S4; Figure 4). Alpha/beta hydrolase (ABH) functions as chaperons and hormone precursors in the stress response process, by way of its fold acting as bona fide ligand receptor in the strigolactone, karrin-smoke receptor, and gibberellin response pathways [67]. Chaperon protein Clp B3 chloroplastic confers thermo-tolerance to chloroplasts during heat stress in Arabidopsis [68]. From these reports, we can conclude that up-regulation of chaperons and USP genes is an important strategy to tolerate drought in maize seedlings.

#### 3.2.4. Proteins/Enzymes Involved in Cellular Detoxification under Drought Stress

Plant stress response process is a complex phenomenon, involving stress signals perception, cell homeostasis adjustment, DNA cell cycle check points arresting, and damage-induced DNA repair processes [9]. In addition, mitogen-activated protein kinase (MAPK) cascades, calcium-regulated proteins, ROS, and transcriptional factors cross-talk are active in stress signaling and defense response and acclimation pathways, rendering the whole network intricate [69]. Generally, ROS perturb cellular redox homeostasis resulting in oxidative damage to many mitochondrial cellular components along with over-reduction of electron transport chain components in the mitochondria, plastids and several detoxification reaction centers. This also results in an imbalance between ROS and the antioxidative defense system [70]. It is critical that proteins involved in redox homeostasis be instituted for fine regulation of the steady state and responsive signaling levels of ROS in order to avoid injury and maintain an appropriate level by which different developmental and environmental signals can be perceived and transmitted [30,71]. Here, we observed that glutathione transferase (B4G1V3), thioredoxin-like protein (A0A1D6K5D2) and ferredoxin-oxidoreductase (COP472) were up-regulated in response to drought (see SD_TD comparison, Figure 4; Table S4).

Glutathione transferases (GSTs) are key cellular detoxification enzymes involved in scavenging of excessive amounts of ROS generated in plant tissues under oxidative stress conditions, and thus, protect plants from oxidative damage [72,73]. They also participate in the signal transduction pathways, cellular responses to auxins and cytokinins, as well as metabolic turnover of cinnamic acid and anthocyanins [74,75]. GSTs have also been up-regulated in response to aluminum toxicity [76]. Ferrodoxin oxido-reductase is vital in oxidation-reduction, electron transfer and signaling processes, as well as catalyzing light dependent photosynthesis [77,78]. Thioredoxins (TRXs) are involved in the protection against oxidative stress as electron donors for thioredoxin peroxidases, which detoxify hydrogen peroxide and alkyl hydroperoxides [79]. Potato plants lacking the CDSP32 plastidic thioredoxin exhibited overoxidation of the BAS1 2-cysteine peroxiredoxin and increased lipid peroxidation in thylakoids under photooxidative stress [79]. Thus, the up-regulation of these antioxidant enzymes herein aids in countering the ROS effects, thereby protecting cells from oxidative damage. Overall, we can suggest that YE8112 endured drought stress better than MO17 because of its enhanced activation of proteins involved in detoxification signaling, response to stress and oxidation-reduction.

However, in the TD_TC comparison, we observed that five proteins involved in stress oxidation-reduction (B6TD62, membrane steroid binding protein; B4FQR3, Aldose reductase; Q84TC2, DIBOA-glucoside dioxygenase BX6; B4FTP2; and H9BG22) and ribosome biogenesis (A0A1D6PT84 and C4J0F8) were differentially down-regulated (Table S2). The down-regulation of these stress redox homeostasis proteins in TD_TC implies the complexity of the cell redox system in stress response. Further, the repression of proteins involved in ribosome biogenesis in leaves of YE8112 may, on one hand, simply indicate the drastic effect of drought on stress-defense protein biosynthesis [80]. However, on the other hand, here, we suggest that the down-regulation of proteins involved in ribosome biosynthesis is an indication that, under drought stress, the tolerant line YE8112 had the ability to reduce the synthesis of redundant proteins, which may help the plant save energy to battle that stress [5,81].

#### 3.2.5. Proteins Related to ‘Response to Stimuli’ under Drought Stress

Several DAPs were enriched in ‘response to stimuli’ under the biological processes (BP) category of the GO functional classification in the tolerant line YE8112 (Table S5). Among the up-regulated DAPs in this function were two uncharacterized proteins (C0HJ06, B6UFE3), two chlorophyll a-b binding proteins (Q41746, B4FL55); Abscic acid stress ripening 1 (B4FKG5), and a universal stress protein (C0HGH7) (Table 2). Additionally, in the SD_TD comparison, cytokinin riboside 5′–monophosphate phosphoribohydrolase protein (A0A1D6NKY3) (*LOG*) was up-regulated in response to drought stress (Figure 4; Table S4). The *LOG* enzyme is involved in cytokinin activation [82]. Cytokinin is a multifaceted phytohormone that plays crucial roles in diverse aspects of plant growth and development, including leaf senescence, apical dominance, lateral root formation, stress signaling and tolerance [83]. Cytokinin signaling cascades are evolutionarily related to the two-component systems that participate in environmental-stimuli-triggered signal transduction [84]. Taken collectively, we can conclude that cytokinin metabolism and signaling; in cross-link with photosynthesis proteins and some chaperons constitute a vital drought response cascade in YE8112.

However, six proteins (A0A1D6IUI1, ubiquitin carboxyl-terminal hydrolase 13; H9BG22, alpha-dioxygenase; A0A1D6PQ00, U2 snRNP auxiliary factor large subunit; B4FTP2, thioredoxin like protein CDSP32; Q5GJ59, terpene synthase 7; COPHF6, AAA-ATPase ASD mitochondrial) were down-regulated in response to drought in the TC_TD group (Table 2). The ubiquitin-dependent proteolytic pathway degrades most proteins and is the primary proteolysis mechanism in eukaryotic cells [85]. Whereas ubiquitin regulates the degradation of proteins, deubiquitinating enzymes (deubiquitinases) play the antagonistic role, therefore reversing the fate of the proteins [86]. Here, the down-regulation of ubiquitin carboxyl-terminal hydrolase 13 implies that cells suppress the proteins and enzymes involved in protein ubiqutination in order to protect themselves against unnecessary protein degradation under drought stress. Alpha-dioxygenase (α-DOX) catalyzes the primary oxygenation of fatty acids into oxylipins, which are important in plant signaling pathways. It has been shown to be up-regulated in response to different abiotic stresses including drought, salt, cold, and heavy metal; and may also be involved in the leaf senescence process [87]. Here we suggest that the down-regulation of α-DOX may be a way to retard leaf senescence in stressed maize seedlings, thereby improving drought tolerance.

Terpenes constitute a large class of secondary metabolites that serve multiple roles in the interactions between plants and their environment, including biotic and abiotic stress responses [88]. They are involved in environmental stimuli perception, stress, and phytohormone signaling [89,90]. In addition, MAPK cascade (signal transduction mechanism) plays an important role in activation and de-activation of enzymes through phosphorylation/de-phosphorylation, which allows for fast and specific signal transduction and amplification of external stimuli [91]. Previous studies [92,93,94] have revealed the role of MAPK cascade in intracellular pathogen immunity and abiotic stress signaling. However, in the current study, MAPK (A0A1D6GZE2) and terpene proteins were down-regulated reflecting the importance and complexity of the cell redox system, signaling, and abiotic-biotic stress cross talks in drought response. Furthermore, splicing is an essential process in eukaryotic gene expression, and the precise excision of introns from premRNA requires a dynamically assembled RNA protein complex (spliceosome). U2 snRNP is one such essential splicing factor that participates in intron and exon definition [95]. Thus, here, the down-regulation of U2 snRNP may imply that mRNA processing is negatively hampered by drought stress.

#### 3.2.6. Key Epigenetic Regulation Mechanisms of the Tolerant Line YE8112

Plants also cope with abiotic stresses by prompt and harmonized changes at transcriptional and post-transcriptional levels, including the epigenetic mechanisms [96]. DNA methylation is essential for stress memory and adaptation in plants [97]. Abiotic or biotic factors can influence gene expression regulation via DNA methylation [98]. In chick pea (*Cicer arietinum* L.) leaf tissues, drought stress triggered DNA hyper-methylation [99]. Combined drought and salinity stresses triggered a shift from C3 to CAM photosynthesis mode in *Mesembryanthemum crystallinum* L. plants, as a result of DNA CpHpG-hypermethylation [100]. In the current study, proteins involved in S-adenosyl-methionine (SAM) dependent methyltransferase (MTases) activity (A0A1D6NE76 and C0HDZ4) were differentially expressed in response to drought stress (Table S2). SAM serves as methyl donor for SAM-dependent methyltransferases (MTases). The resultant transmethylation of biomolecules constitutes a significant biochemical mechanism in epigenetic regulation, cellular signaling, and metabolite degradation [101]. The DEP C0HDZ4 encode the maize gene ZEAMMB73_Zm0001d009084 and is important for DNA methylation. Thus, here, YE8112 induced dynamic DNA methylation alterations as part of a complex drought-stress response network, with bias towards down-regulation of SAM-D-MTase. Furthermore, acetyltransferase (B6UHR7) was up-regulated in YE8112 (see the SD_TD comparison, Table S4). Histone acetyltransferases (HATs) play an important role in eukaryotic transcriptional activation in the epigenetic regulation process [102]. Thus, the key epigenetic regulation mechanisms in YE8112 were DNA methylation (via down-regulation of overlapping protein A0A1D6NE76) and enhanced histone acetylation through up-regulation of HATs related proteins.

### 3.3. Drought Responsive DAPs Observed in Sensitive Inbred-Line MO17

The iTRAQ analysis identified a higher number of DAPs in MO17 than in YE8112 in response to drought stress (compare Table 2 and Table 3). Variation in abundance of the DAPs in response to drought stress implies specific sensitivity or adaptation of these two maize lines [30]; the two inbred line plants detected the extent of the same drought stress conditions differentially. Drought tolerant-line YE8112 might have perceived the prevailing drought conditions as mild and then modulated fewer DAPs, whilst sensitive-line MO17 perceived the same conditions as severe and modulated more abundant DAPs in response.

#### 3.3.1. Enhanced Expression of Heat Shock Proteins (HSP20-Like Chaperons) and 50S Ribosomal Proteins Constitutes a Critical Defensive Response in MO17

Among the dominating up-regulated DAPs in MO17, we observed heat shock proteins (HSP 20-like chaperons superfamily), chaperon DNA-J domain superfamily proteins and ribosomal proteins (50S Ribosomal protein L20) (Table 3). Molecular chaperons facilitate the stabilization of other macromolecular structures, including other proteins, under stress conditions [80]. Precisely, heat shock proteins (HSPs) are vital in protecting plants against stress by preserving other proteins in their functional confirmations [103]. HSPs have been greatly accumulated in alfalfa (*Medicago sativa* L.) leaves in response to salinity stress [104]. As anticipated, the increased accumulation (up-regulation) of HSPs could be regarded as a crucial defensive response of MO17 against drought stress. Additionally, ribosomal proteins (40S, 50S, and 60S) are an integral component of stress-defense protein biosynthesis machinery [105], hence were up-regulated under drought stress. Similarly, Ziogas et al. [106] found out that the 40S and 60S ribosomal proteins were up-regulated in citrus response to PEG-induced osmotic stress.

#### 3.3.2. Up-Regulation of Cell Detoxification and Photosynthesis Related Proteins May Contribute to Enhanced Drought Stress Tolerance in MO17

Superoxide dismutase protein (B4F925), together with the photosynthesis related proteins: chlorophyll a-b binding protein (B4FV94), oxygen evolving enhancer protein (B6SUJ9), photosystem II CP47 reaction center protein (A0A1X7YHJ3), and pyruvate phosphate dikinase proteins were up-regulated in response to drought stress (Table 3). Enhanced antioxidant enzyme activity is a part of an array of complex detoxification and defense mechanisms to protect cells from the oxidative damage by excessive ROS [9]. Enhanced accumulation of SOD proteins suggests that the activation of enzymatic antioxidant systems is a crucial protective mechanism for drought stressed MO17. The SOD and oxygen evolving enhancer proteins may increase drought tolerance by playing a role in cellular detoxification and protecting cells from oxygen toxicity [80,85]. Photosystem II proteins, together with other auxiliary proteins, enzymes, or components of thylakoid protein trafficking/targeting systems, are directly or indirectly involved in de novo assembly and/or the repair and reassembly cycle of PSII [107,108]. Pyruvate phosphate dikinase (PPDK) is one of the most important enzymes in C_4_ photosynthesis, catalyzing the reversible phosphorylation of pyruvate to phospho*enol*pyruvate, thus, the most crucial rate-limiting C_4_ cycle enzyme [109,110]. Taken collectively, the above results indicated that the up-regulation of cell detoxification and photosynthesis enhancing proteins constitute a vital drought stress response strategy in the sensitive maize inbred-line MO17.

#### 3.3.3. Glutathione Transferases and Ca^2+^-Dependent Kinases Negatively Influenced by Short Term Drought Stress

Among the down-regulated DAPs in MO17 were those associated with signaling recognition, especially glutathione transferases (GSTs; A0A1D6M4E1) and calcium dependent protein kinase (A0A1D6ICZ3) (Table 3). The GSTs are key participants in plant growth and development, shoot regeneration processes, and adaptability to adverse environmental stimuli [72]. Crucially, GSTs are major cellular detoxification enzymes protecting plants from oxidative damage [73]. Calcium-dependent protein kinases (CDPKs) represent potential Ca^2+^ decoders to translate developmental and environmental stress cues [111,112]. However, the down-regulation of DAPs regulating these enzymes herein implies that short-term drought stress negatively influenced the signal transduction processes involving these enzymes.

#### 3.3.4. Key Epigenetic Regulation Mechanisms of the Sensitive Line MO17

In addition to the DNA methylation related protein A0A1D6NE76 (overlapping between the two inbred lines; down-regulated in YE8112, but up-regulated in MO17), we also observed proteins associated with histones (histones H2A and H1) to be down-regulated in response to drought stress (Table 3). Histone modification is the key epigenetic regulation mechanisms in plants and eukaryotic cells [113]. Phosphorylation of H2A histones functions in DNA double strand breaks (DSBs) repair [114]. Thus, whilst DNA methylation (through down-regulation of related proteins) and histone acetylation were dominant epigenetic regulation mechanisms in YE8112, DNA methylation (via up-regulation of related proteins) and histone modification (probably phosphorylation; via down-regulation of H2A and H1 proteins) were preferred in MO17 in response to drought stress.

### 3.4. Overlapping Drought Responsive Proteins Between YE8112 and MO17 under Drought Conditions

Venn diagram (Figure 3) analysis showed that only five significant DAPs were common between TD_TC and SD_SC. All the 5 proteins (Table 5) were down-regulated in tolerant line YE8112 in response to drought treatment. Comparably, among these five common proteins, two (ribose-phosphate pyrophosphokinase and uncharacterized protein C4JOF8) were down-regulated, whilst the other 3 (membrane steroid binding protein 1, monosaccharide transporter 1, SAM-dependent methyltransferase superfamily protein) were up-regulated in sensitive line MO17 in response to drought treatment. Moreover, the two common down-regulated proteins showed similar fold changes in both inbred lines under drought stress (Table 5). In *Arabidopsis thaliana*, membrane steroid binding protein 1 (MSBP1) is involved in inhibition of cell elongation [113]. Additionally, Yang et al. [114] realized that the inhibitory effects by 1-N-naphthylphthalamic acid (NPA), an inhibitor of polar auxin transport, are suppressed under the MSBP1 overexpression, suggesting the positive effects of MSBP1 on polar auxin transport. They concluded that MSBP1stimulates tropism by regulating vesicle trafficking and auxin redistribution in Arabidopsis seedling roots. Here, we suggest that maize seedlings endure drought stress by down-regulating MSBP1 in tolerant line YE8112, but up-regulating (overexpression) it in sensitive line MO17, as a way to enhance cell elongation and growth under stress. Ribose-phosphate pyrophosphokinase (PRPP synthetase) catalyzes the nucleotide biosynthesis process. PRPP is an essential substrate for purine and pyrimidine nucleotides, both in the de novo synthesis and in the salvage pathway [115]. In the current study, therefore, the down-regulation of the PRPP synthetase enzyme in both inbred lines under drought stress is consistent with the inhibition of nucleotide biosynthesis as a general feature of abiotic stresses. Moreover, our observation that an uncharacterized protein C4J0F8 was down-regulated, and at the same fold change in both lines, suggests that the protein has a common function in the two maize inbred lines’ drought stress responses. This could serve as a targeted protein for further elucidation in our future studies.

Monosaccharide transporters (MSTs) are integral membrane proteins whose trans-membrane-spanning domains interact to form a central pore that shuttles soluble monosaccharides across hydrophobic membranes [116]. Expression of plant MST genes is also regulated by environmental stimuli such as pathogen infection (AtSTP4) [117] or wounding (AtSTP3 and AtSTP4) [118]. The MSTs catalyze monosaccharide import into classic sinks such as root tips and anthers, and, most importantly, help to meet the increased carbohydrate demand of cells responding to environmental stress [117]. Based on these discussions, we herein suggest MSTs to play an important adaptive role in the supply of carbohydrates to rapidly growing or metabolically hyperactive cells or tissues fighting drought stress, especially in sensitive line MO17, whilst down-regulation in tolerant line YE8112 may imply genotype diversity and the negative effects of drought stress on carbohydrates translocation in YE8112. The SAM synthetase gene is expressed in all living cells, and its product, Sadenosyl-l-methionine, is the major methyl donor in all cells [119]. Previously, the expression of SAM synthetase in soybean root was shown to be decreased upon exposure to drought stress [120]. Here, we state that, on one hand, the down-regulation of this enzyme in tolerant inbred YE8112 is consistent with the inhibition of photosynthetic activity as a general feature of abiotic stresses. On the other hand, this observation may imply SAM-dependent methyltransferase (SAM-D-Mtases) protein’s variability in epigenetic mechanism (DNA methylation) regulation, as determined by genotypic differences, considering that the same protein was up-regulated in sensitive line MO17 in response to drought stress.

### 3.5. Significantly Enriched Metabolic Pathways of DAPs under Drought Stress

Metabolic adaptation of plants exposed to different stress requires sophisticated metabolic reorganization of multiple metabolic pathways [80], hence, we employed KEGG pathway enrichment analysis to identify key pathways related to drought stress response in maize seedlings. Photosynthesis antenna proteins pathway was the most significantly enriched, followed by proline metabolism and biosynthesis of amino acids pathways (Figure 6A). Photosynthesis of C_4_ plants is highly sensitive to drought stress [121,122]. Chloroplasts, particularly the thylakoid membranes—PSII reaction centers, are one of the organelles most influenced by drought stress [54,123]. In the current study, the protein (B4FL55) encoding the *Lhcb5-2* gene and protein (Q41746) encoding *Lhcb5-1* gene were up-regulated in both inbred lines and significantly enriched in the photosynthesis (antenna protein) pathway (Table 2 and Table 3; Figure 6A). These proteins are a part of the light harvesting complexes (LHCs) and the electron transport components of the photosystem II (PSII) of the plant photosynthesis machinery [124]. They act as peripheral antenna systems enabling more efficient absorption of light energy [125]. Further, Lhch5-1 is involved in the intracellular non-photochemical quenching and the cysteine biosynthesis processes [124]. Previously, Zhao et al. [85] observed photosynthesis as the top signaling pathway affected by drought stress in maize, with chlorophyll a-b binding protein being up-regulated in an ABA-dependent manner. Remarkably, Dudhate et al. [126] also observed photosynthesis pathway to be highly enriched in pearl millet in response to drought stress. Taken together, these proteins play critical roles in light capture and utilization balancing to avoid photoinhibition (photodamage or photoinactivation) of the PSII due to excess light, as well as electron transport system, thus their involvement in photosynthesis pathway in tolerant line YE8112.

Comparatively, drought sensitive line MO17 showed two significantly enriched pathways, C5-branched dibasic acid metabolism (C5-BDAM) and RNA transport (Figure 6D). In *Physcomitrella patens* L., the C5-BDAM pathway has been observed critical in protoplast reprogramming to stem cells during the process of cell division [127]. In a stage-specific analysis, C5-BDAM pathway is specifically enriched from 24 h to 48 h during the process (a stage of stem cell re-entering cell cycle). Together with other pathways such as pentose phosphate pathway and leucine and isoleucine biosynthesis, C5-BDAM is closely associated with cell fate transition during protoplast reprogramming into stem cells [127]. Transport of RNAs from the nucleus to the cytoplasm, as ribonucleoprotein complexes (RNPs), is functionally coupled to gene expression processes such as splicing and translation [128]. Here, we suggest that translation and post translational processes are altered by drought stress as the cells modulate gene expressions related to stress tolerance, more prominently in sensitive line MO17. Similarly, Zhao et al. [85] observed RNA transport pathway to be significantly enriched in maize leaves in response to drought. For the pictorial view of the two most significantly enriched pathways described herein, please refer to Figure S6.

### 3.6. Function-Unknown Proteins Identified Under Drought Stress Conditions

We identified proteins with known critical roles in drought stress responses, together with unknown or predicted proteins that may have important functions in the regulatory network for drought stress. Of the 37 DAPs identified in tolerant-line YE8112, seven were of unknown functions, including four (B6SQW8, C0HJ06, B6UFE3, and C0P948) up-regulated and three (A0A1D6MJP2, B4F845, and C4J0F8) down-regulated. Interestingly, protein B6SQW8 was the most significantly expressed in tolerant line (TD_TC) (Table S2). Additionally, out of the 157 DAPs identified in sensitive-line (SD_SC), thirty were uncharacterized proteins, including 12 up-regulated and 18 down-regulated (Table S3). Moreover, one unknown protein (C4J0F8) was observed to overlap and exhibited a similar expression pattern (down-regulation) under drought stress, suggesting it has a common stress response function in the two inbred lines. These stress-responsive proteins with predicted functions may confer drought tolerance. Therefore, further studies of these proteins will help elucidate the molecular mechanisms underlying drought stress responses of maize lines differing in drought tolerance.

### 3.7. Protein-Protein Interaction (PPI) Analysis

Proteins in the cell are usually found as complexes, and biological processes within the cell are controlled by interactions between various proteins [119]. Therefore, identifying potential protein partners and studying protein–protein interactions becomes imperative for drought stress response research. Here, we used String 10.5 database analysis to determine how the identified differentially abundant proteins interact with others in networks to effect specific cellular functions. Some of the drought responsive proteins were predicted to interact with each other and hold central positions in certain PPI networks whereas some nodes showed no direct connections (Figure 7). The linkages created by these identified proteins in interaction networks can provide deeper insights into their relative importance in biological processes. ‘Protein hubs’ (connected to various other proteins) such as uncharacterized protein (Zm45026) in YE8112 and elongation factor 1-alpha (GRMZM2G343543_P03) in MO17; and ‘bottlenecks’ (key connectors of sub-networks), such as electron transporter/thiol-disulfide exchange intermediate (GRMZM5G869196_P01) in YE8112 and hypothetical protein LOC100383576 (AC234515.1_FGP003) in MO17 represent central points for communication co-ordination within the interaction network and tend to play critical roles in drought stress responses.

Analysis of PPI networks in tolerant inbred line YE8112 (Figure 7A) revealed that the interaction constituted by proteins involved in stress signaling, maintaining antioxidant defense, electron transport, and amino acid (protein) metabolism occupied a central position and may play a critical role in maize seedling drought stress responses. In addition, another protein interaction made up of proteins involved in energy metabolism, amino acid metabolism, maintaining redox homeostasis, and epigenetic regulation was prominent in YE8112. Moreover, a smaller connection had proteins involved in energy (NADH) metabolism and secondary metabolite (polyamines and proline) synthesis. These observations confirm the importance of these metabolic processes in drought stress response as revealed previously [71,80,85,103,105].

Most hub proteins in the larger complex and small networks in MO17 such as elfa3 (elongation factor 1-alpha), 50S Ribosomal protein L2 (rpl2-A), plastid specific 30S ribosomal protein 2 (GRMZM2G143870) and GRMZM2G343543_P03 were involved in protein biosynthesis and de-ubiquitination, suggesting these processes are critical drought responses [105,106], in sensitive line MO17. Furthermore, the several nodes that are not connected with other proteins within the interaction networks (for example Lhcb1 and aba1 in YE8112, and GRMZM5G826321_P01 in MO17) showed that those proteins did not interact with others based on the String database analysis [30]. However, these proteins may play indirect roles in maize seedling responses to drought stress.

### 3.8. Proposed Models of Drought Stress Tolerance in Maize Seedlings

**Figure 9:**
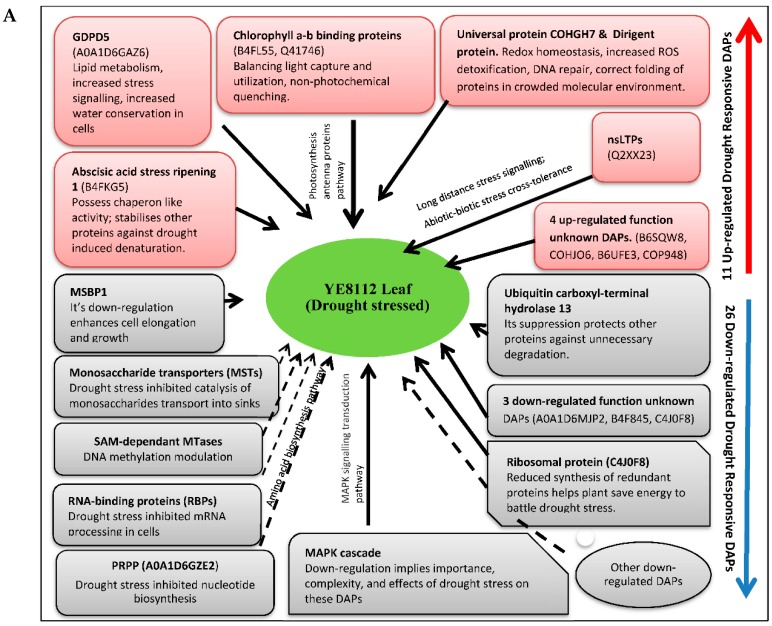

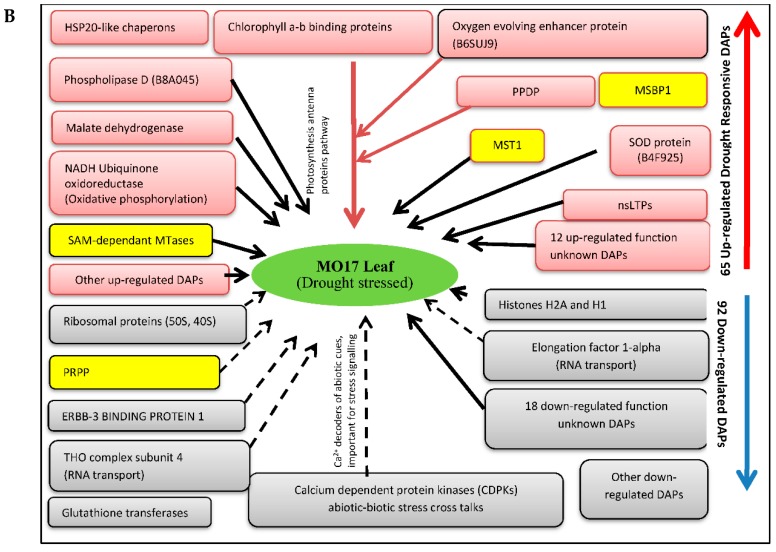
Molecular models of drought tolerance in maize seedling leaves of: (**A**) tolerant inbred line YE8112 and (**B**) sensitive line MO17. Red nodes (rectangles/circles) signify up-regulated DAPs; gray nodes signify down-regulated DAPs; yellow nodes in MO17 model (Figure 9B) represents overlapping DAPs also observed in YE8112. Dotted black connectors/arrows imply drought stress imposed negative effects on respective proteins or pathways; compound type black connectors imply desirable drought stress response outcomes on respective proteins. Note: nsLTPs, non-specific lipid transfer proteins; GDPD5, Glycerophosphodiester phosphodiesterase 5; MSBP1, membrane steroid binding protein 1; MAPK, mitogen-activated protein kinases; PRPP, Ribose-phosphate pyrophosphokinase; MST1, monosaccharide transporter 1; PPDP, pyruvate phosphate dikinase proteins.

Based on the annotated biological functions and the relevant published literature on the key drought responsive/related proteins or genes identified in the current study, we have developed models for drought stress tolerance in maize as shown below (Figure 9).

## 4. Materials and Methods

### 4.1. Plant Materials and Drought Stress Treatment

Two maize (*Zea mays* L.) inbred lines (ILs) with contrasting drought sensitivity (tolerant YE8112 and sensitive MO17) were used in this experiment. Seeds of the two inbred lines were provided by the North China Key Laboratory for Crop Germplasm Resources of Education Ministry, Hebei Agricultural University, China. In selecting the two ILs, we employed our lab screening on seedling survival rates of dozens of maize inbred lines under drought stress treatment; this finding was supported with previous experiments [38,39]. Seeds were surface sterilized in 10% hydrogen peroxide for 5 min, followed by washing three-times with sterile water. Then, the seeds were germinated by laying them between two layers of damp filter paper at 28 °C for 24 h according to the procedures of Lei et al. [10]. Germinated seeds were placed in the same size PVC pots with uniform soil and grown under greenhouse controlled conditions (light/dark cycles: 14/10; 28/22 °C; 60 ± 5% relative humidity) at Hebei Agricultural University, Baoding, China. Maize seedlings were grown under normal conditions until the three leaves were fully expanded. Then, both the tolerant and sensitive inbred lines were exposed to drought conditions for a 7-day period. For both tolerant and sensitive lines, a half of the plants were subjected to drought by withholding irrigation to 50% soil moisture content (which was detected using a TZS-1 soil moisture meter, Zhejiang Top Cloud-Agri Technology Co., Ltd., Hanzhou; China) and the rest of the plants were grown under well-watered condition (control). Flag leaves from the control and drought stress treated plants collected after 1, 3, 5, and 7 days of treatment (for physiological analyses), and collected once at 7 days post treatment exposure (for proteomic analysis) were immediately frozen in liquid nitrogen and stored at −80 °C prior to respective analyses. Each treatment was replicated three times.

### 4.2. Phenotypic and Physiological Characterizations

Phenotypic and physiological characterizations were measured for the YE8112 and MO17 seedlings under well-watered and drought-stress conditions. Relative water content (RWC) was estimated according to Galmés et al. [129]. Trypan blue staining of the leaves of both inbred lines under water-deficit conditions was also conducted [130,131]. The leaf peroxidase (POD) activity was estimated by the guaiacol method [132]. The level of lipid peroxidation (MDA content) in the leaves was measured by thiobarbituric acid (TBA) method [133]. The osmolytes proline content was determined using ninhydrin as per the protocol of Bates et al. [134].

### 4.3. Protein Extraction

Total proteins were extracted from the non-stressed and stressed leaf tissues of two maize inbred lines with three biological replicates (each containing 500 mg maize leaves) using the cold acetone method as described in previous reports [30,135]. In brief, samples were ground to a powder in liquid nitrogen and lysed with 2 mL lysis buffer containing 8 M urea, 2% SDS, and 1× Protease Inhibitor Cocktail (Thermo Fisher Scientific, Shanghai, China). Then, the solution was kept on ice for 30 min prior to centrifugation at 11,500 rpm (18,000× *g*) for 15 min at 4 °C. The supernatant was then transferred into a new tube and precipitated with 10% TCA/90% acetone, followed by incubation at −20 °C overnight. Pellets were washed thrice with acetone. Finally, the precipitate was dissolved in 8 M urea under ultrasound irradiation. Total protein concentrations of the extracts were determined using a Coomassie Bradford Protein Assay Kit (23200, Thermo Fisher Scientific, Shanghai, China), with bovine serum albumin (BSA) as standard, according to the manufacturer’s instructions. The absorbance was determined at 562 nm using an xMark microplate absorbance spectrophotometer (Bio-Rad Laboratories Inc., Hercules, CA, USA), and protein extracts quality was examined with SDS-PAGE (tricine-sodium dodecyl sulfate polyacrylamide gel electrophoresis) [136].

### 4.4. Protein Digestion and iTRAQ (Isobaric Tags for Relative and Absolute Quantification) Labeling

For each sample, the solution was transferred to a new tube and adjusted to 100 μL using 8 M urea, mixed with 11 μL 1 M DTT, and incubated at 37 °C for 1 h followed by centrifugation at 4 °C at 14,000× *g* for 10 min. The supernatant was incubated in a dark room for 20 min after the addition of 120 μL 55 mM iodacetamide. This followed washing of the supernatant using 100 μL mM TEAB (triethylammonium bicarbonate) and centrifugation at 14,000× *g* for 10 min at 4 °C, followed by discarding the eluate. This washing step was repeated thrice before trypsin digestion. Total proteins were digested using trypsin (Promega, Madison, WI, USA) at a ratio of protein:trypsin = 30:1 at 37 °C overnight (16 h). The peptides were dried in a centrifugal vacuum concentrator and reconstituted in 0.5 M TEAB. Detailed protein digestion procedures are contained in a previous report [80].

Protein iTRAQ labeling was conducted by Applied Protein Technology Co., Limited (Shanghai, China) using an iTRAQ Reagents 8-plex kit (AB Sciex, Foster City, CA, USA) according to the manufacturer’s protocol. In brief, one unit of iTRAQ reagent (defined as the amount of reagent required to label 100 μg of protein) was thawed and reconstituted in 70 μL isopropanol. The control replicates were labeled with iTRAQ tag 115 for the drought-sensitive inbred line (MO17) and tag 117 for drought-tolerant inbred line (YE8112). The drought treated replicates were labeled with tags 114 and 116 for drought-sensitive and drought–tolerant lines, respectively. Three technical replicates were performed.

### 4.5. Strong Cation Exchange (SCX) and LC-MS/MS Analysis

Sample fractionation was conducted before LC-MS/MS (liquid chromatography-tandem mass spectrometry) analysis as described in previous report by Ross et al. [36] with some modifications. Briefly, the iTRAQ labeled peptide mixtures were separated by strong cation exchange (SCX) chromatography on an Agilent 1100 HPLC system (Agilent Technologies, Waldbronn, Germany) using a PolySulfoethyl A column (4.6 × 100 mm^2^, 5 μm, 300 Å; PolyLC, Columbia, MD, USA) as per the manufacturer’s guidelines. The sample was dissolved in 4 mL of SCX loading buffer (25% *v/v* ACN, 10 mΜ KH_2_PO_4_, pH 3, with phosphoric acid), loaded and washed isocratically for 20 min at 0.5 mL/min to remove excess reagent. The retained peptides were eluted with a linear gradient of 0–500 mM KCl (25% *v*/*v* ACN, 10 mΜ KH_2_PO_4_, pH 3) over 15 min at a flow rate of 1 mL/min, with fractions collected at 1 min intervals. The elution was monitored by measuring absorbance at 214 nm, and the eluted peptides were pooled into 10 fractions.

Each SCX fraction was subjected to reverse phase nanoflow HPLC separation and quadruple time-of-flight (QSTAR XL) mass spectrometry analysis. Protocols for the analysis of reverse phase nanoflow HPLC and tandem mass spectrometry have been explicitly described in previous reports [80,137]. In short, peptides were subjected to nano electrospray ionization followed by tandem mass spectrometry (MS/MS). The mass spectrometry was analyzed by Q-Exactive mass spectrometer (Thermo Fisher Scientific, Shanghai, China) after the sample had been analyzed by chromatography. The MS spectra with a mass range of 300–1800 *m*/*z* were acquired at a resolving power of 120 K, the primary mass spectrometry resolution of 70,000 at 200 *m*/*z*, AGC (automatic gain control) target of 1*e*6, maximum IT of 50 ms, and dynamic exclusion time (active exclusion) of 60.0 s The mass charge ratio of polypeptides and polypeptide fragments were set according to the following parameters: 20 fragments (MS2 scan) were collected after each scan (full scan), MS2 activation type was HCD, isolation window 2 *m*/*z*, two-grade mass spectrometry resolution of 17,500 at 200 *m*/*z*, the normalized collision energy of 30 eV, underfill of 0.1%. The electrospray voltage applied was 1.5 kV. Maximum ion injection times for the MS and MS/MS were 50 and 100 ms, respectively.

### 4.6. Protein Identification and Quantification

All of the mass spectrometry data from the LC-MS/MS raw files were obtained using Mascot software version 2.2 (Matrix Science, London, UK) and converted into MGF files using Proteome Discovery 1.4 (Thermo Fisher Scientific Inc., Waltham, MA, USA). For protein identification, MGF data files from the LC-MS/MS were searched against the Uniprot database (available online: https://www.uniprot.org; accessed on 12 January 2018; uniprot*_Zea mays*_132339_20180112.FASTA; 76,417 sequences) using Mascot search engine. The search parameters were set as follows: trypsin as the cleavage enzyme; two maximum missed cleavages allowed; fragment mass tolerance was set at ±0.1 Da; and peptide mass tolerance was set at ±20 ppm; monoisotopic as the mass values; iTRAQ 8 plex (Y) and Qxidation (M) as variable modifications; and Carbamidomethyl (C), iTRAQ 8 plex (N-term) and iTRAQ 8 plex (K) selected as fixed modifications. Only peptides with a false discovery rate (FDR) estimation ≤1% and a 95% confidence interval were counted as being successfully identified.

As described in a previous study [137], protein relative quantification was dependent on the reporter ions ratios, from which relative peptides abundance can be estimated. Only proteins that were present in all the samples were considered for quantification; shared peptides were omitted. Reporter ion ratios determination used the peak intensities of the reporter ions, with control-treated YE8112 sample serving as reference. Further normalization of the final protein quantification ratios was conducted using the median average of those ratios. The unique peptide ratios’ median represented the protein ratio. Student’s *t*-test was used to analyze the differentially abundant proteins (DAPs), with proteins exhibiting fold-changes >1.2 or <0.83 (*p* < 0.05) considered to be statistically significant DAPs [138].

### 4.7. DAPs Functional Classification, Pathway Enrichment, and Hierarchal Clustering Analysis

The successfully identified DAPs were used as queries to search the Interpro (https://www.ebi.ac.uk/interpro/) and Pfam (http://pfam.xfam.org/); Gene Ontology (GO) (http://www.geneontology.org/) and the KEGG (http://www.genome.jp/kegg/) databases. The corresponding gene sequences of the DAPs were obtained by searching the maize sequence database Gramene (http://ensemble.gramene.org/Zea_mays/). GO analysis [139] was used for functional annotation and classification of the DAPs identified to describe the biological processes, cellular component, and molecular functions involved in the response to drought stress. Additionally, GO (protein) terms were assigned to each DAP based on BLASTX similarity (E-value < 1.0 × 10^5^) and known GO annotations, using the Blast2GO tool (available online: https://www.blast2go.com; accessed on 6 February 2018) [140]. The DAPs were assigned to various biological pathways using the KEGG pathway analysis. Further, significant KEGG pathway enrichment analysis was performed using the hypergeometric test, with Q (Bonferroni-corrected *p*-value) less than 0.05 defined as statistically significant. A protein interaction network was constructed using the String program (version 10.5) (http://www.string-db.org/).

### 4.8. RNA Extraction, cDNA Synthesis, and RT-qPCR Analysis

Total RNA was isolated from non-stressed and stressed seedling leaves of the two inbred lines (YE8112 and MO17) and prepared for qRT-PCR analysis using the Omini Plant RNA Kit (DNase I) (CWBIO, Beijing, China) based on the manufacturer’s instructions. For cDNA synthesis, 1 μg of total RNA was reverse-transcribed in a total volume of 20 μL, using HiFiscript cDNA Synthesis Kit (CWBIO, Beijing, China) according to the manufacturer’s instructions. Thirty DAPs were selected and gene-specific primers (Table S7) designed for qRT-PCR using Primer Premier 5 Designer software. qRT-PCR was conducted with a C1000 (CFX96 Real-Time System) Thermal Cycler (Bio-Rad) using 2× Fast Super EvaGreen^®^ qPCR Mastermix (US Everbright Inc., Suzhou, China). Each qRT-PCR reaction mixture comprised 1 μL of template cDNA, 1 μl of forward primer (50 pmol), 1 μL of reverse primer (50 pmol), and 10 μL of 2×Fast Super EvaGreen^®^ qPCR Mastermix (US Everbright Inc., Suzhou, China) in a total reaction volume of 20 μL. The amplification program was set as follows: 95 °C for 2 min followed by 40 cycles of 95 °C for 10 s and 55 °C for 30 s [80]. A steady and constitutively expressed maize gene *GAPDH* (accession no. X07156) was used as the internal reference gene, together with the forward primer (GAPDH-F: 5′-ACTGTGGATGTCTCGGTTGTTG-3′) and reverse primer (GAPDH-R: 5′-CCTCGGAAGCAGCCTTAATAGC-3′). Each sample had three technical replicates [2]. The relative mRNA abundance was calculated according to the 2^−ΔΔCT^ method [141].

### 4.9. Statistical Data Analysis of Physiological Changes

Physiological data analysis was performed using the SPSS statistical software package (version 19.0; SPSS Institute Ltd., Armonk, NY, USA), and the significance of differences were tested by Fisher′s protected least significant differences (PLSD) test with a *p*-value ≤ 0.05 set as statistically significant.

## 5. Conclusions

In the present study, we conducted a comprehensive comparative analysis of two maize inbred lines contrasting in drought stress tolerance based on their physiological and proteomic responses. Our results have shown that divergent stress tolerance mechanisms exist between the two lines at the seedling stage. Both qualitative and quantitative differences, at physiological and proteomic levels, showed that YE8112 is comparatively more tolerant to drought stress than MO17 owing to its maintenance of higher RWC and proline contents, higher increase in POD enzyme activity, along with decreased level of lipid peroxidation under stressed conditions. Using an iTRAQ-based method, we obtained a total of 721 differentially abundant proteins (DAPs). Amongst these, we identified five essential sets of drought responsive DAPs, including 13 DAPs specific to YE8112, 107 DAPs specific to TD_SD comparison, three DAPs of YE8112 also regulated in TD_SD, 84 DAPs unique to MO17, and five overlapping DAPs between the two inbred lines. The most significantly enriched proteins in YE8112 were associated with the photosynthesis antenna proteins pathway, whilst those in MO17 were related to C5-branched dibasic acid metabolism and RNA transport pathways. The changes in protein abundance were consistent with the observed physiological characterizations of the two inbred lines. Further, our qRT-PCR analysis results confirmed the iTRAQ sequencing based findings. We have clarified the two maize inbred lines’ strategies to tolerate drought stress and elucidated the fundamental molecular networks associated. Based on our findings, and relevant literature cited herein this report, we have proposed molecular models of drought tolerance in the two inbred line seedling leaves as provided in Figure 9. The higher drought stress tolerance of YE8112 may be attributed to: (a) Activation of photosynthesis (PSII) proteins involved in balancing light capture and utilization, and improving non-photochemical quenching; (b) Enhancement of lipid-metabolism related proteins, contributing to increased stress signaling and water conservation in the cell. Furthermore, plants may have developed abiotic-biotic stress cross-tolerance mechanisms; (c) Stimulation of chaperons such as ASR1 protein in order to stabilize a number of other proteins against drought-induced denaturation; (d) Increased cell ROS detoxification capacity; (e) Reduced synthesis of redundant proteins to help the plant save energy to battle drought stress; and (f) Suppression of protein ubiqutination in order to protect proteins against unnecessary degradation under drought stress, and thus, reversing the fate of those proteins. These results provide more insights into the physiological and molecular mechanisms underpinning drought stress tolerance in maize seedlings.

## Figures and Tables

**Table 1:** Number of differentially abundant proteins (DAPs) identified in each comparison group.

Comparisons ^1^	Up-Regulated ^2^	Down-Regulated ^3^	Total ^4^
SD_SC	65	92	157
TD_TC	11	26	37
SD_TD	116	153	269
SC_TC	119	139	258

^1^ Comparisons, differential comparison groups; SD, sensitive inbred line (MO17) under drought treatment conditions; SC, sensitive inbred line under well-watered (control) conditions; TD, tolerant inbred line (YE8112) under drought conditions; TC, tolerant inbred line under control conditions; ^2^ up-regulated: increased differential abundant protein; ^3^ down-regulated: reduced differential abundant protein; ^4^ Total: total of all the differentially abundant proteins in a comparison group. An underscore between two line-treatment combinations implies comparison of those combinations.

**Table 2:** Drought-responsive maize seedling leaf proteins observed specifically in tolerant line YE8112.

No.	Protein ID ^1^	Gene Name/ID ^2^	Description ^3^	Coverage (%) ^4^	Peptide Fragments ^5^	Fold Change ^6^	*p* Value ^7^	Pathways ^8^
1	C0HJ06	541618	Uncharacterized protein ^9^	22.4	1	1.37	0.0109	MAPK signaling pathway/Plant hormone signaling
2	Q41746	Lhcb5-1	Chlorophyll a-b binding protein, chloroplastic	55.8	10	1.24	0.0131	X3
3	C0HGH7	100193714	Universal stress family protein	20.4	3	1.23	0.0430	
4	A0A1D6GAZ6	ZEAMMB73_Zm00001d012677	Glycerophosphodiester phosphodiesterase GDPD5	16.8	5	1.22	0.0136	Glycerophospholipid metabolism
5	C0P948	Zm00001d024886	Uncharacterized protein	55.9	20	1.21	0.0350	
6	A0A1D6PQ00	100286059	U2 snRNP auxiliary factor large subunit	9.4	2	0.83	0.0171	Spliceosome
7	A0A1D6IUI1	100383306	Ubiquitin carboxyl-terminal hydrolase 13	2.7	3	0.83	0.0217	
8	A0A1D6MJP2	ZEAMMB73_Zm00001d039613	Uncharacterized protein	19.9	4	0.82	0.0111	
9	B4FTP2	ZEAMMB73_Zm00001d021334	Thioredoxin-like protein CDSP32 chloroplastic	23.7	6	0.81	0.0246	
10	B4F845	100191245	Uncharacterized protein	3.0	1	0.81	0.0027	
11	H9BG22	101027254	Alpha-dioxygenase	4.4	3	0.80	0.0162	alpha-linolenic acid metabolism
12	Q5GJ59	TPS7	Terpene synthase 7	14.8	5	0.78	0.0179	
13	C0PHF6	100383595	AAA-ATPase ASD mitochondrial	10.6	5	0.55	0.0487	

^1^ Protein ID, unique protein identifying number in the UniProt database; ^2^ Gene name/ID; name or ID number of the corresponding gene of the identified differentially abundant protein as searched against the maize sequence database Gramene (http://ensemble.gramene.org/Zea mays); ^3^ Description, annotated biological functions based on Gene Ontology (GO) analysis; ^4^ Coverage (%), sequence coverage is calculated as the number of amino acids in the peptide fragments observed divided by the protein amino acid length; ^5^ Peptides fragments, refer to the number of matched peptide fragments generated by trypsin digestion; ^6^ Fold change, is expressed as the ratio of intensities of up-regulated or down-regulated proteins between drought stress treatments and control (well-watered conditions); All the fold change figures below 1 represents that the proteins were down-regulated. All the figures above 1 means the proteins were up-regulated; ^7^ *p* value, statistical level (using Student’s *t*-test) below <0.05, at which protein differential expression was accepted as significant; ^8^ Pathways, metabolic Kyoto Encyclopedia of Genes and Genomes (KEGG) pathways in which the identified protein was found to be significantly enriched; ^9^ uncharacterized protein, a protein without any functional annotations ascribed to it at the present.

**Table 3:** Drought-responsive maize seedling leaf proteins observed specifically in sensitive line MO17.

No.	Protein ID ^1^	Gene Name/ID ^2^	Description ^3^	Coverage (%) ^4^	Peptide Fragments ^5^	Fold Change ^6^	*p* Value ^7^	Pathways ^8^
1	B4FV94	Zm00001d032197	Chlorophyll a-b binding protein, chloroplastic	49.8	7	1.66	0.0326	Photosynthesis-antenna proteins
2	B4FCG6	Zm00001d004386	^9^ Uncharacterized protein	9.0	1	1.48	0.0036	
3	B4FTN5	100273215	Metal-dependent protein hydrolase	5.7	1	1.45	0.0459	
4	B8A3B7	Zm00001d043059	Uncharacterized protein	20.8	3	1.33	0.0278	
5	C0P6L9	Zm00001d053377	Uncharacterized protein	40.2	7	1.33	0.0011	Ribosome
6	B4FLE3	100282216	HSP20-like chaperones superfamily protein	33.0	4	1.32	0.0484	
7	B6U3Z0	Zm00001d053377	50S ribosomal protein L21	42.5	7	1.31	0.0149	Ribosome
8	K7TP80	Zm00001d024014	Zinc finger (C3HC4-type RING finger) family protein	36.0	14	1.31	0.0028	
9	A0A1D6JW44	Zm00001d028428	Calcium-binding EF-hand family protein	9.0	1	1.30	0.0014	
10	A0A097PND9	Zm00001d015195	AT5G11810-like protein (Fragment)	6.9	1	1.29	0.0358	
11	B4FE30	100193174	10 kDa chaperonin	45.9	5	1.29	0.0024	
12	B4FZU8	100274264	Malate dehydrogenase	56.8	12	1.28	0.0150	Carbon metabolism, Pyruvate metabolism, Cysteine and methionine metabolism
13	Q4A1J8	cc3	Cysteine proteinase inhibitor	11.3	1	1.28	0.0293	
14	A0A1X7YHJ3	Zm00001d000282	Photosystem II CP47 reaction center protein	46.9	16	1.28	0.0062	Photosynthesis
15	B4FWP6	Zm00001d039452	Uncharacterized protein	9.9	4	1.27	0.0374	Spliceosome
16	B4FTL2	Zm00001d044931	Protein TIC 22 chloroplastic	9.3	2	1.27	0.0001	
17	C0P8X5	100284068	Electron transfer flavoprotein subunit beta mitochondrial	14.9	1	1.25	0.0020	
18	A0A1D6HE45	ZEAMMB73_Zm00001d017330	ATP-dependent Clp protease proteolytic subunit	33.7	5	1.25	0.0218	
19	Q2XX37	plt2	Non-specific lipid-transfer protein	46.2	4	1.25	0.0435	
20	A0A1D6JYF7	103634473	Kinesin-like protein	3.1	1	1.24	0.0409	
21	A0A1D6E501	ZEAMMB73_Zm00001d002880	3-isopropylmalate dehydrogenase	50.1	12	1.24	0.0449	Oxocarboxylic acid metabolism, C5-Branched dibasic acid metabolism, Biosynthesis of amino acids
22	A0A1D6L0Y0	ZEAMMB73_Zm00001d033634	Uncharacterized protein	7.6	1	1.24	0.0111	
23	A0A096PRE6	100282938	Fibrillin1	31.4	9	1.23	0.0421	
24	K7UWX4	ZEAMMB73_Zm00001d051062	GrpE protein homolog	44.2	11	1.23	0.0083	
25	B4FMA5	100217267	Chaperone DnaJ-domain superfamily protein	14.6	2	1.23	0.0378	
26	B7ZZT1	Zm00001d027326	Uncharacterized protein	6.5	1	1.22	0.0039	
27	B8A045	100279815	Phospholipase D	2.9	2	1.22	0.0211	Endocytosis, Ether lipid metabolism, Glycero phospholipid metabolism
28	B6TGF1	Zm00001d009640	Malate dehydrogenase 2 mitochond.	72.4	14	1.22	0.0092	Carbon metabolism, Pyruvate metabolism, Cysteine and methionine metabolism, Carbon fixation in photosynthetic organisms
29	A0A1D6FI49	ZEAMMB73_Zm00001d009189	TPR repeat	6.0	1	1.22	0.0283	
30	B6UHD9	Zm00001d021715	Peptide chain release factor 2	8.0	2	1.22	0.0374	
31	B6TDF7	100282980	Plastid-specific 30S ribosomal protein 2	45.4	9	1.21	0.0014	RNA transport, RNA degradation, mRNA surveillance pathway
32	Q1KKB7	nad1	NADH-ubiquinone oxidoreductase chain 1	5.9	1	1.21	0.0355	Oxidative phosphorylation
33	A0A059Q7D4	psbD	Photosystem II D2 protein	25.2	7	1.20	0.0058	Photosynthesis
34	C4J3Q4	100277436	YCF37-like protein	17.7	2	1.20	0.0017	
35	B4FTK9	100282281	Alpha/beta-Hydrolases superfamily protein	33.6	6	1.20	0.0421	
36	B6TBW4	100282838	ERBB-3 BINDING PROTEIN 1	30.5	10	0.83	0.0174	
37	A0A1D6DVJ8	ZEAMMB73_Zm00001d002006	H(+)-ATPase 5	34.6	18	0.83	0.0322	Oxidative phosphorylation
38	A0A1D6DYT2	100383868	Signal recognition particle 14 kDa protein	11.3	1	0.83	0.0172	Protein export
39	B6T346	100279524	THO complex subunit 4	14.2	3	0.83	0.0390	mRNA surveillance pathway, RNA transport
40	A0A1D6GKY6	100192032	Uncharacterized protein	4.9	1	0.83	0.0411	
41	B6SJ21	100280585	Guanine nucleotide-binding protein beta subunit-like protein	59.3	13	0.83	0.0232	
42	C0PI72	Zm00001d017459	Uncharacterized protein	8.3	1	0.82	0.0201	Valine, leucine and isoleucine degradation
43	C0HI59	100381692	Uncharacterized protein	13.3	5	0.82	0.0181	
44	A0A1D6M4E1	ZEAMMB73_Zm00001d038192	Glutathione transferase41	8.6	1	0.82	0.0025	Glutathione metabolism
45	A0A1D6GES6	103625778	DNA gyrase subunit A chloroplastic/mitochondrial	1.9	1	0.81	0.0372	
46	B6TIL4	Zm00001d048954	GDP-mannose 3,5-epimerase 2	20.5	6	0.81	0.0265	Amino sugar and nucleotide sugar metabolism, Ascorbate and aldarate metabolism
47	B6T3J2	100282096	Eukaryotic translation initiation factor 2 beta subunit	12.9	3	0.81	0.0206	RNA transport
48	A0A1D6F8L4	100194138	Coatomer subunit gamma	7.2	4	0.81	0.0316	
49	C0PI69	Zm00001d040286	Uncharacterized protein	18.5	2	0.81	0.0092	
50	A0A0B4J3C2	ZEAMMB73_Zm00001d037873	Elongation factor 1-alpha	42.1	15	0.81	0.0463	RNA transport
51	B4FEV5	Zm00001d031689	Uncharacterized protein	13.8	1	0.81	0.0400	Plant-pathogen interaction
52	P26566	rpl20	50S ribosomal protein L20, chloroplastic	20.2	3	0.81	0.0476	Ribosome
53	A0A1D6KBW7	ZEAMMB73_Zm00001d030317	Hsp20/alpha crystallin family protein	17.8	2	0.81	0.0098	
54	A0A1D6ICZ3	542526	Calcium dependent protein kinase8	7.0	3	0.80	0.0465	Plant-pathogen interaction
55	B4FAJ4	Zm00001d008739	Uncharacterized protein	2.8	1	0.80	0.0260	Peroxisome
56	B6T9T5	N/A	Uncharacterized protein	4.3	1	0.80	0.0002	
57	Q9M7E2	Zm00001d036904	Elongation factor 1-alpha	30.7	10	0.80	0.0134	RNA transport
58	B7ZZ42	103650526	Heat shock 70 kDa protein 3	58.6	30	0.80	0.0076	Spliceosome, Endocytosis, Protein processing in endoplasmic reticulum
59	A0A1D6N9X4	103651144	Insulin-degrading enzyme-like 1 peroxisomal	3.5	3	0.79	0.0149	
60	A0A1D6IHP2	103633334	ARM repeat superfamily protein	6.5	5	0.79	0.0161	
61	B4FLV6	100286322	Protein translation factor SUI1	20.0	3	0.79	0.0269	RNA transport
62	B4FQM2	100282190	Pyrophosphate--fructose 6-phosphate 1-phosphotransferase subunit beta	6.7	2	0.79	0.0123	Fructose and mannose metabolism, Pentose phosphate pathway, Glycolysis/Gluconeogenesis
63	B6TP02	Zm00001d017866	Aspartic proteinase nepenthesin-1	5.6	2	0.78	0.0276	
64	A0A1D6PW61	100191474	DNA topoisomerase 1 beta	3.1	1	0.78	0.0189	
65	B6SR37	Zm00001d011799	Uncharacterized protein	17.3	2	0.78	0.0070	
66	A0A1D6JQY8	100192907	Uroporphyrinogen-III synthase	2.8	1	0.78	0.0294	Porphyrin and chlorophyll metabolism
67	A0A1D6IIC2	ZEAMMB73_Zm00001d021999	Nuclear transport factor 2 (NTF2) family protein	5.6	1	0.77	0.0092	
68	B6U4J6	Zm00001d045774	Embryogenesis transmembrane protein	4.5	1	0.77	0.0258	
69	C0P626	Zm00001d011454	Carbonic anhydrase	74.3	13	0.77	0.0272	Nitrogen metabolism
70	Q9M7E3	Zm00001d009868	Elongation factor 1-alpha	37.8	13	0.76	0.0045	RNA transport
71	B6SI29	100501869	Histone H2A	29.3	4	0.76	0.0326	
72	B4FIA6	100194327	Histone H2A	28.9	3	0.76	0.0406	
73	A0A1D6JVL9	ZEAMMB73_Zm00001d028377	Small nuclear ribonucleoprotein Sm D3	21.7	2	0.75	0.0384	Spliceosome
74	B6SLI1	100282946	40S ribosomal protein S30	16.1	1	0.74	0.0110	Ribosome
75	A0A1D6LBT4	100279572	Protein prenyltransferase superfamily protein	7.0	1	0.72	0.0475	
76	A0A1D6P0E7	ZEAMMB73_Zm00001d046001	Triose phosphate/phosphate translocator TPT chloroplastic	22.1	2	0.72	0.0111	
77	B4FFS7	Zm00001d036233	Uncharacterized protein	7.8	1	0.71	0.0347	
78	A0A1D6FPL0	100382596	Fructose-16-bisphosphatase cytosolic	21.2	8	0.70	0.0179	Fructose and mannose metabolism, Pentose phosphate pathway
79	Q8LLS4	Pgk-1	Phosphoglycerate kinase (Fragment)	32.2	9	0.69	0.0440	Carbon metabolism, Glycolysis/Gluconeogenesis.
80	A0A1D6K8W1	ZEAMMB73_Zm00001d030005	Dynamin-related protein 1E	2.7	1	0.68	0.0411	
81	A0A1D6QSH1	100383873	Cullin-associated NEDD8-dissociated protein 1	3.6	3	0.65	0.0139	
82	B6TNP4	Zm00001d034479	Histone H1	41.0	11	0.65	0.0485	
83	A0A1D6MEZ2	ZEAMMB73_Zm00001d039282	Serine/threonine-protein kinase AGC1-5	1.4	1	0.55	0.0120	
84	E7DDW6	Zm00001d026630	Clathrin light chain 2	23.0	4	0.52	0.0203	

^1^ Protein ID, unique protein identifying number in the UniProt database; ^2^ Gene name/ID; name or ID number of the corresponding gene of the identified differentially abundant protein as searched against the maize sequence database Gramene (http://ensemble.gramene.org/Zea mays); ^3^ Description, annotated biological functions based on Gene Ontology (GO) analysis; ^4^ Coverage (%), sequence coverage is calculated as the number of amino acids in the peptide fragments observed divided by the protein amino acid length; ^5^ Peptides fragments, refer to the number of matched peptide fragments generated by trypsin digestion; ^6^ Fold change, is expressed as the ratio of intensities of up-regulated or down-regulated proteins between drought stress treatments and control (well-watered conditions); All the fold change figures below 1 represents that the proteins were down-regulated. All the figures above 1 means the proteins were up-regulated; ^7^ *p* value, statistical level (using Student’s *t*-test) below <0.05, at which protein differential expression was accepted as significant; ^8^ Pathways, metabolic pathways in which the identified protein was found to be significantly enriched; ^9^ uncharacterized protein, a protein without any functional annotations ascribed to it at the present.

**Table 4:** Drought responsive DAPs of the tolerant line that were also differentially expressed between the tolerant and sensitive lines after drought treatment.

No.	Protein ID ^1^	Gene Name/ID ^2^	Description ^3^	Coverage (%) ^4^	Peptide Fragments ^5^	YE8112 Fold Change ^6^	*p* Value ^7^	SD_TD Fold Change ^8^	*p* Value ^7^	Pathways ^9^
1	B6SQW8	Zm00001d024893	Uncharacterized protein	27.2	3	1.59	0.0155	0.53	0.0093	No significant enrichment
2	B4FKG5	542304	Abscisic acid stress ripening 1	47.1	4	1.34	0.0096	0.60	0.0325	No significant enrichment
3	A0A1D6HWS1	100282063	Dirigent protein	34.3	4	1.29	0.0207	0.67	0.0118	Not significant enrichment

^1^ Protein ID, unique protein identifying number in the UniProt database; ^2^ Gene name/ID; name or ID number of the corresponding gene of the identified differentially abundant protein as searched against the maize sequence database Gramene (http://ensemble.gramene.org/Zea mays); ^3^ Description, annotated biological functions based on Gene Ontology (GO) analysis; ^4^ Coverage (%), sequence coverage is calculated as the number of amino acids in the peptide fragments observed divided by the protein amino acid length; ^5^ Peptides fragments, refer to the number of matched peptide fragments generated by trypsin digestion; ^6^ YE8112 fold change, is expressed as the ratio of intensities of up-regulated or down-regulated proteins between drought stress and control (well-watered) conditions; ^7^ *p* value, statistical level (using Student’s *t*-test) below <0.05, at which protein differential expression was accepted as significant; ^8^ SD_TD fold change, is the ratio of intensities of up-regulated or down-regulated proteins between drought stressed sensitive line and drought stressed tolerant line; All the fold change figures below 1 represents that the proteins were down-regulated. All the figures above 1 means the proteins were up-regulated; ^9^ Pathways, metabolic pathways in which the identified protein was found to be significantly enriched.

**Table 5:** Common (overlapping) drought-responsive seedling leaf DAPs between MO17 and YE8112.

No.	Protein ID	Gene Name/ID	Description	Coverage (%)	Peptide Fragments	YE8112		MO17		Pathways
						Fold Change	*p* Value	Fold Change	*p* Value	
**1**	B6TD62	100282951	Membrane steroid-binding protein 1	35.8	5	0.81	0.0223	1.50	0.0142	
**2**	A0A1D6GZE2	100272744	Ribose-phosphate pyrophosphokinase	5.4	1	0.82	0.0078	0.82	0.0068	Purine metabolism/Carbon metabolism/Pentose phosphate pathway
**3**	C4J0F8	Zm00001d038865	Uncharacterized protein	32.5	4	0.80	0.0090	0.81	0.0465	Ribosome
**4**	C0PHL2	Zm00001d018627	Monosaccharide transporter1	3.8	1	0.79	0.0051	1.69	0.0495	
**5**	C0HDZ4	Zm00001d009084	SAM-dependent methyltransferase superfamily protein	14.1	2	0.73	0.0218	1.52	0.0245	

For full description of the column items, please refer to Table 2, Table 3 and Table 4 captions above.

## Supplementary Materials

Supplementary materials can be found at http://www.mdpi.com/1422-0067/19/10/3225/s1.

## Abbreviations

ASR1	Abscisic acid stress ripening 1
CDPKs	Calcium dependent protein kinases
DAPs	Differentially abundant proteins
GDPD	Glycerophosphodiester phosphodiesterase
GO	Gene ontology
GST	Glutathione-S-transferase
HSPs	Heat shock proteins
iTRAQ	Isobaric tags for relative and absolute quantification
KEGG	Kyoto Encyclopedia of Genes and Genomes
LC-MS/MS	Liquid chromatography-tandem mass spectrometry
MAPK	Mitogen-activated protein kinases
MDA	Malondialdehyde
MSBP1	Membrane steroid binding protein 1
MSTs	Monosaccharide transporters
nsLTPs	Non-specific lipid transfer proteins
PPI	Protein-protein interaction
POD	Peroxidases
PRPP	Ribose-phosphate pyrophosphokinase
qRT-PCR	Quantitative real-time polymerase chain reaction
RBPs	RNA binding proteins
ROS	Reactive oxygen species
SAM	S-adenosyl-methionine
SAM-d-Mtases	SAM-dependent methyltransferases
SOD	Superoxide dismutase
TRX	Thioredoxin
