# Supplementary material for: Comparative Proteomic and Physiological Analyses of Two Divergent Maize Inbred Lines Provide More Insights into Drought-Stress Tolerance Mechanisms
Source: Int J Mol Sci. 2018 Oct 18;19(10):3225. doi: 10.3390/ijms19103225 (PMC6213998; doi:10.3390/ijms19103225)
Supplement: Supplementary file 1 [file ijms-19-03225-s001.zip › Supplementary Material/Supplementary Figures.docx]

**SUPPLEMENTARY FIGURES**


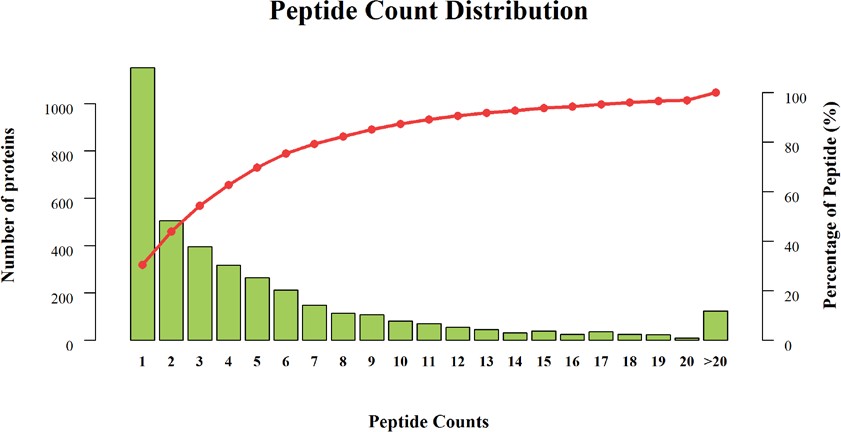

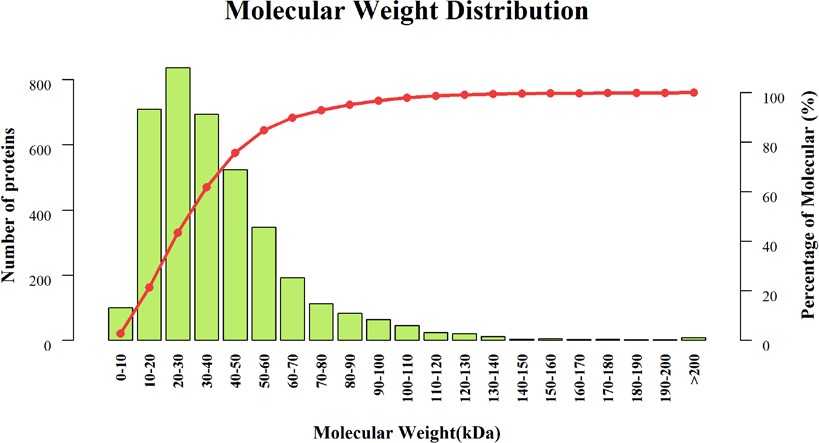


**B**

**A**


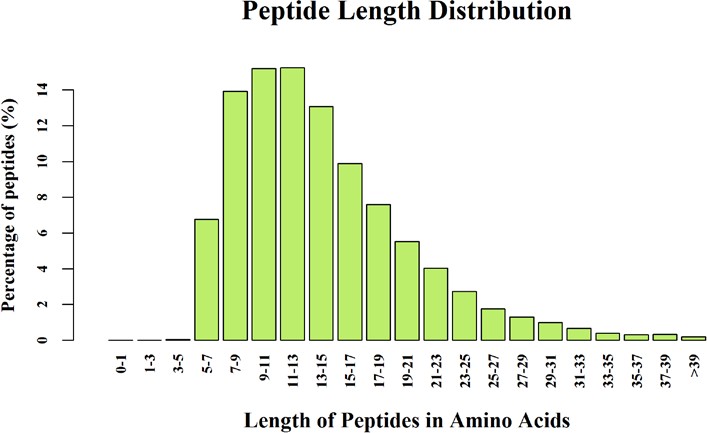

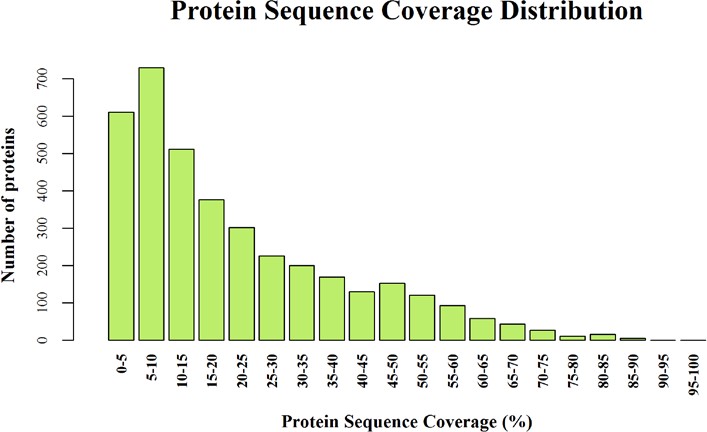


**D**

**C**

**Supplementary Figure 1** Basic iTRAQ output details. **(A)** Mass distribution of the identified proteins; **(B)** numbers of peptides that were matched to proteins; **(C)** distribution of protein`s sequences coverage; and **(D)** peptide length distribution.


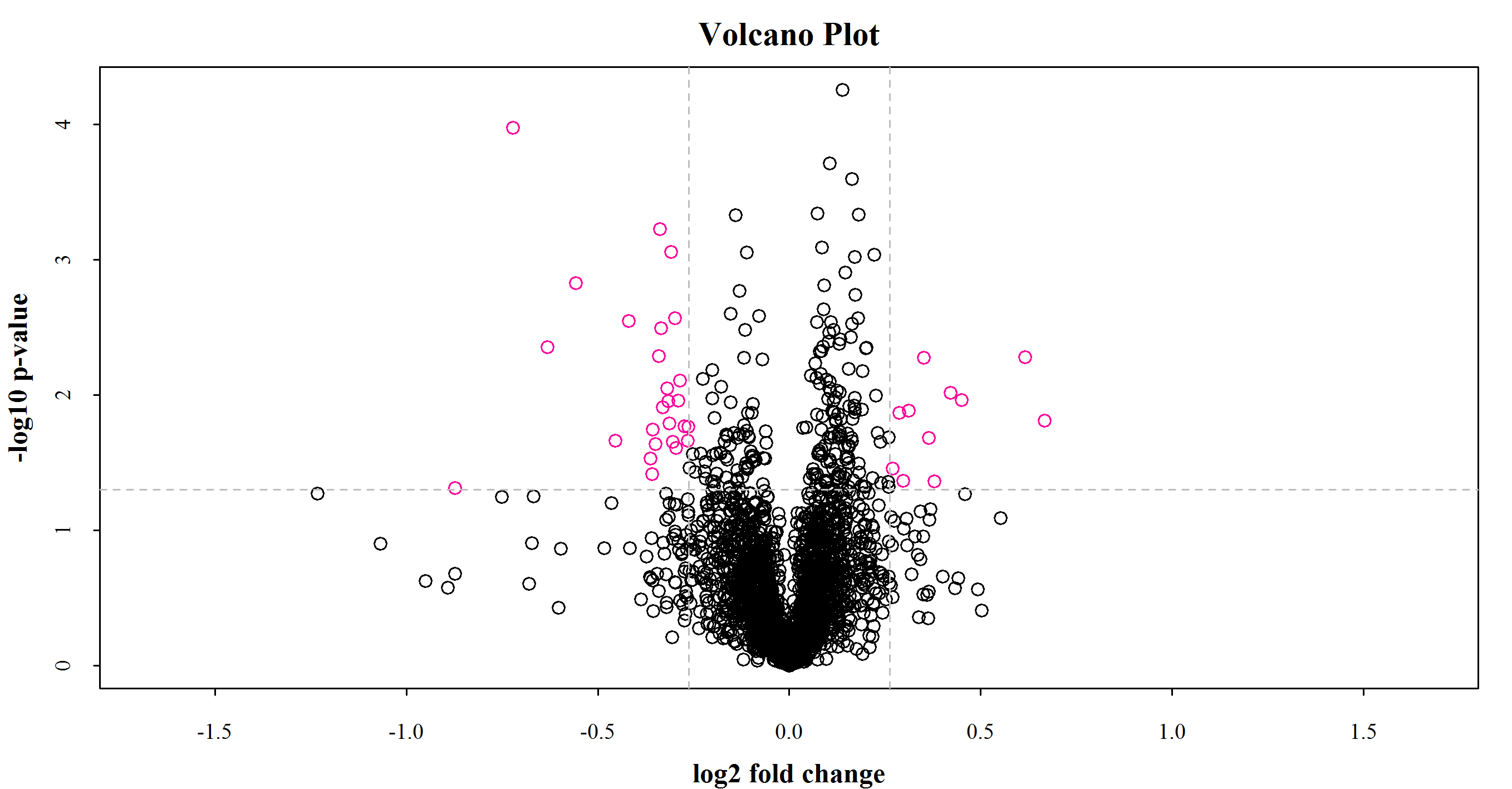


**Above** - Supplementary Figure 2A; **Below**- Supplementary Figure 2B


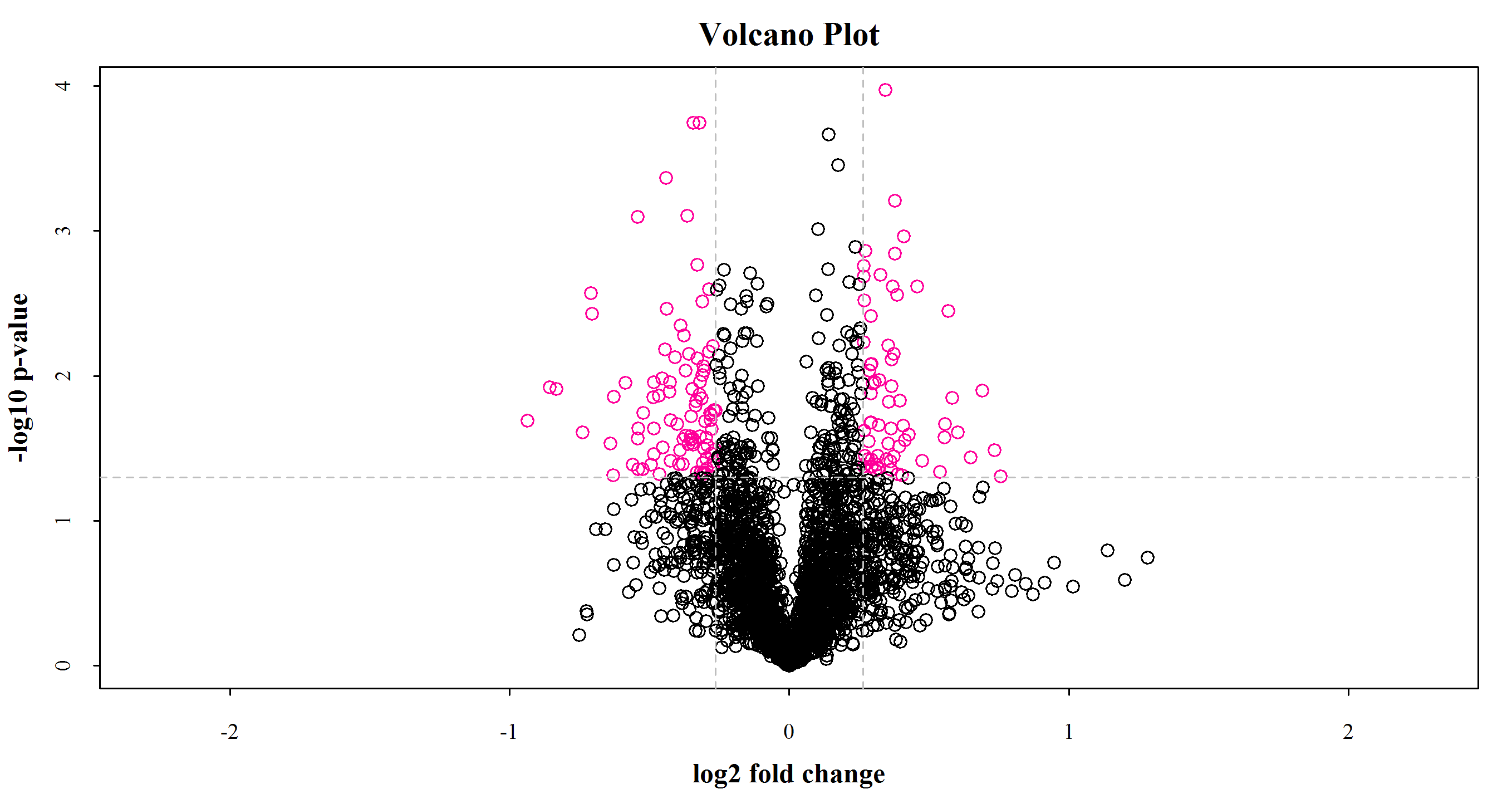


**Supplementary Figure** **2:** Volcano plot showing quantitative and differential analysis of DAPs in tolerant line YE8112 (**A**), and sensitive line MO17 (**B**) under drought stress. The horizontal axis shows the log 2 fold change expression; the vertical axis shows the significant different p-value (log 10 transformation). The red dots are significant differentially abundant proteins (multiples vary more than 1.2 times and P value <0.05), and black dot are no differential changes in the proteins.

**A**

**B**


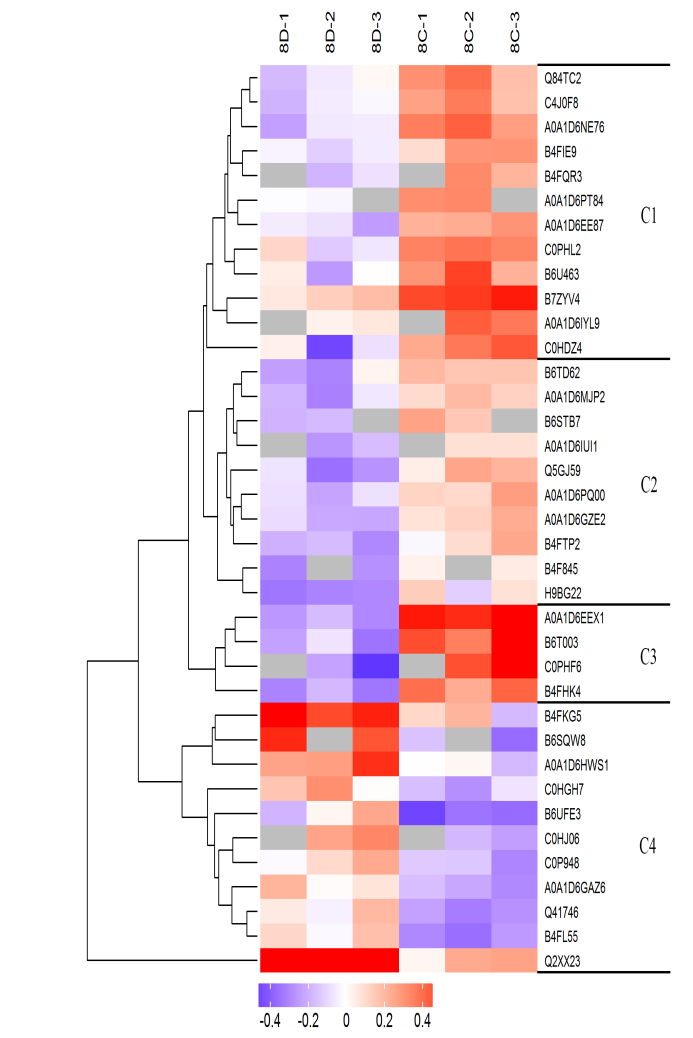

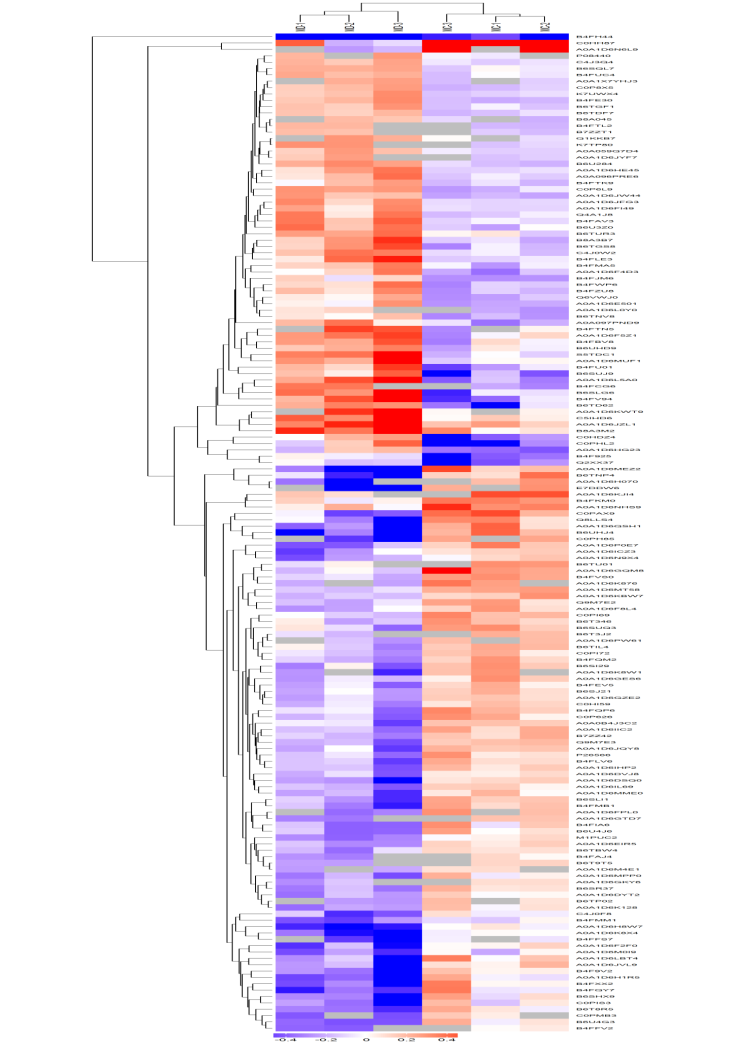


**Supplementary Figure 3**. Clustering analysis of significant DAPs in **(A)** YE8112 before and after drought treatment (TD_TC); **(B)** MO17 before and after drought treatment (SD_SC). Each row represents a protein significantly abundantly expressed. Columns represent technical sample replicates for drought treatment, (8D1-3) and (MD1-3) for YE8112 and MO17, respectively; 8C1-3 and MC1-3 showing sample replicates under water sufficient (control) conditions for YE8112 and MO17, respectively. The scale bar on the X-axis indicates the logarithmic value (log 2 expression) of the expression of significant DAPs in different samples, up-regulated (red) and down-regulated (blue). Basically, more DAPs showed higher expression (and up-regulation) in MO17 than YE8112.


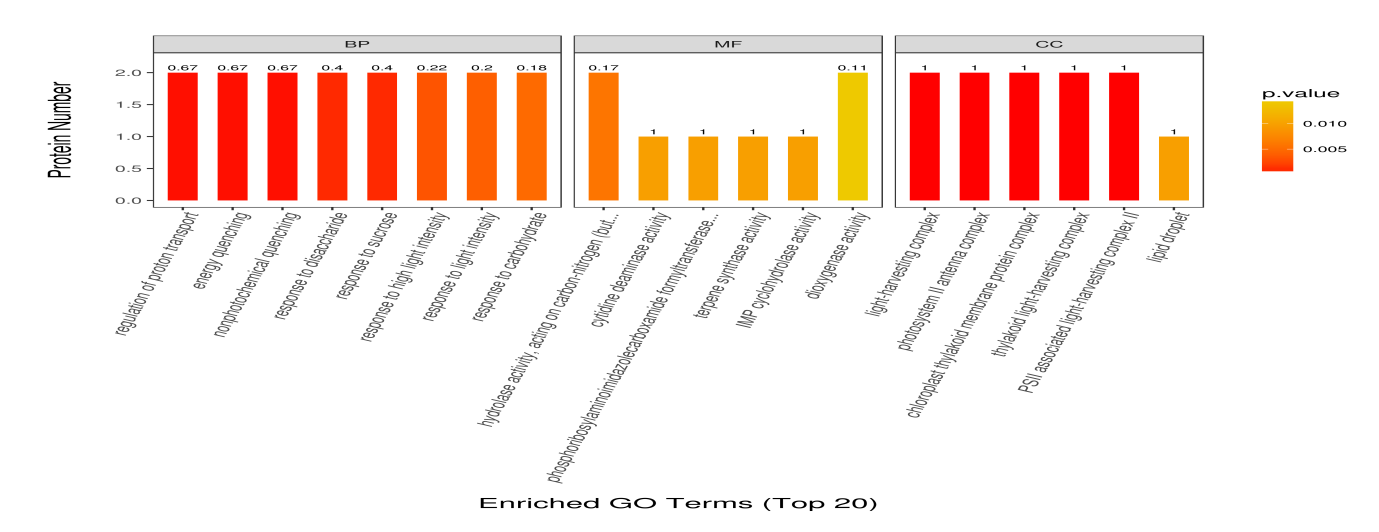


**A**


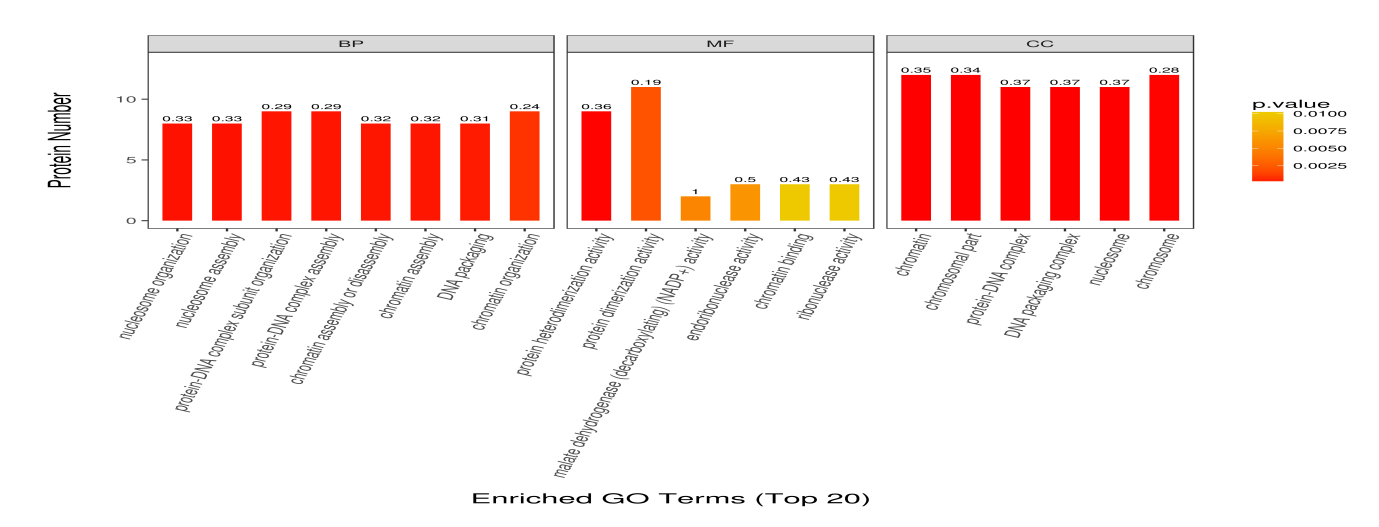


**B**


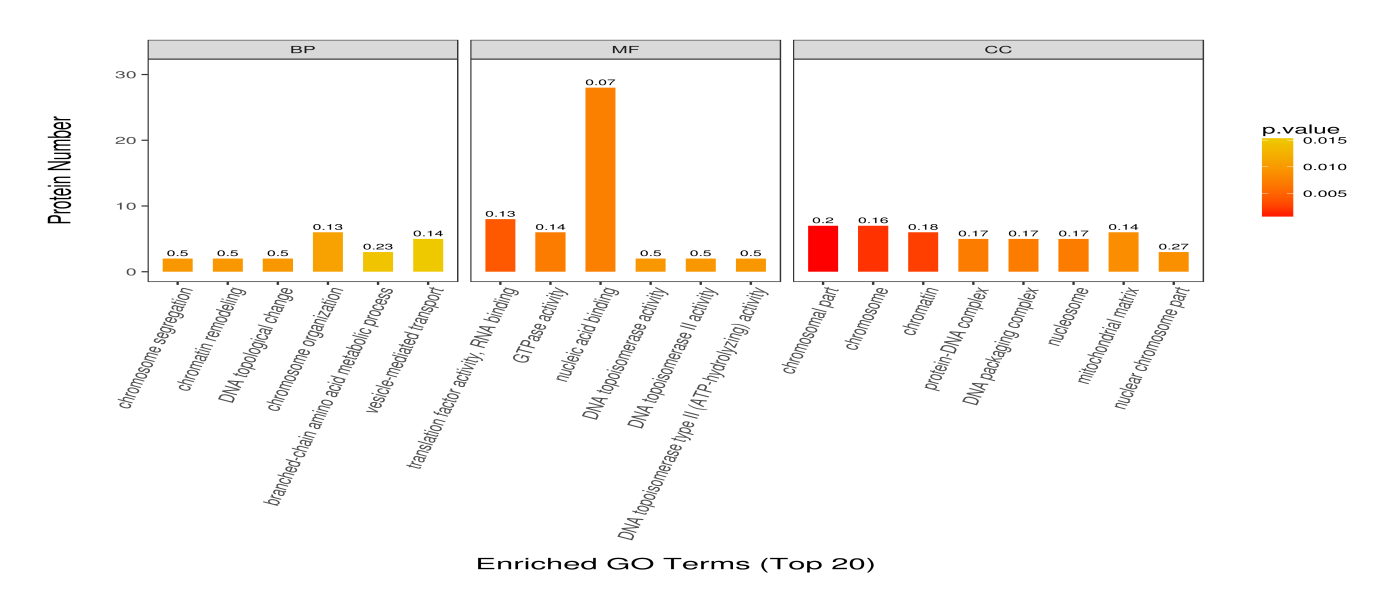


**C**

**Supplementary Figure 4**. Significantly enriched GO terms (Top 20) in the biological functions categories in (A) YE8112-specif DAPs; (B) SD_TD DAPs; and (C) MO17-specific DAPs. The significance of the enrichment of the GO term is based on the Student`s t-test, *p* < 0.05. The color gradient represents the size of the *P* value; the color is from yellow to red, and the nearer red represents the smaller the *P* value, the higher the significant level of enrichment of the corresponding GO term. The number above the bar graph shows the enrichment factor (rich factor ≤ 1).

**Supplementary Figure 5** Validation of iTRAQ-seq expression data through qRT-PCR analysis. Validation was performed using genes derived from four main groups of drought responsive DAPs identified in the study (See Tables 2-5 and Areas I-IV of Figure 3 in the manuscript). The plots demonstrate the expression ratio in Log scale with base of two. The X-axis indicates qRT-PCR Log scale; the Y-axis indicates RNA-seq Log scale.


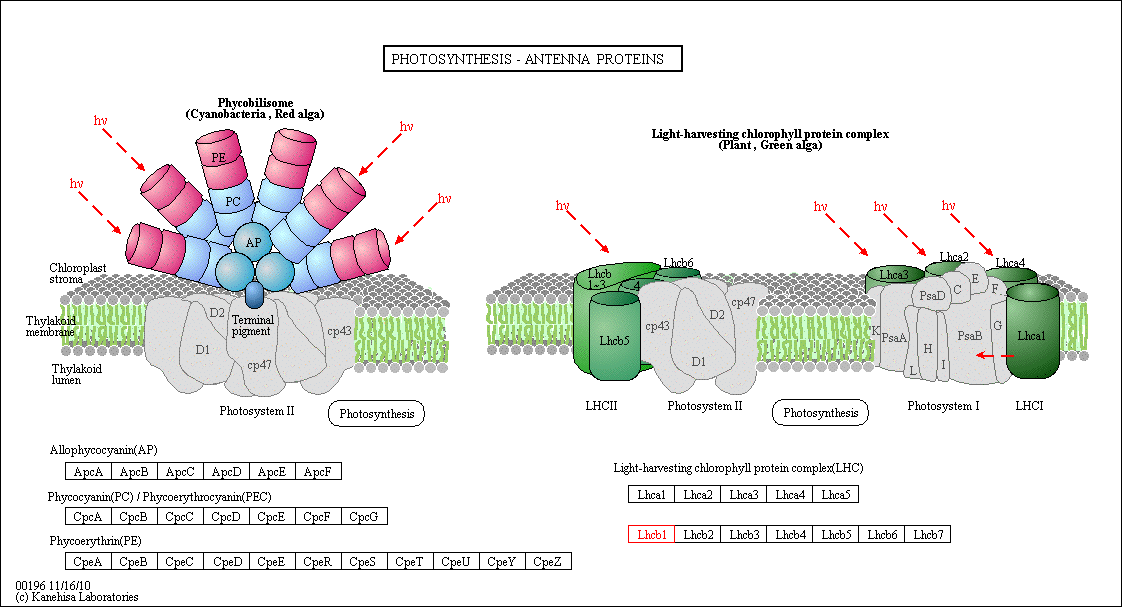


**A**


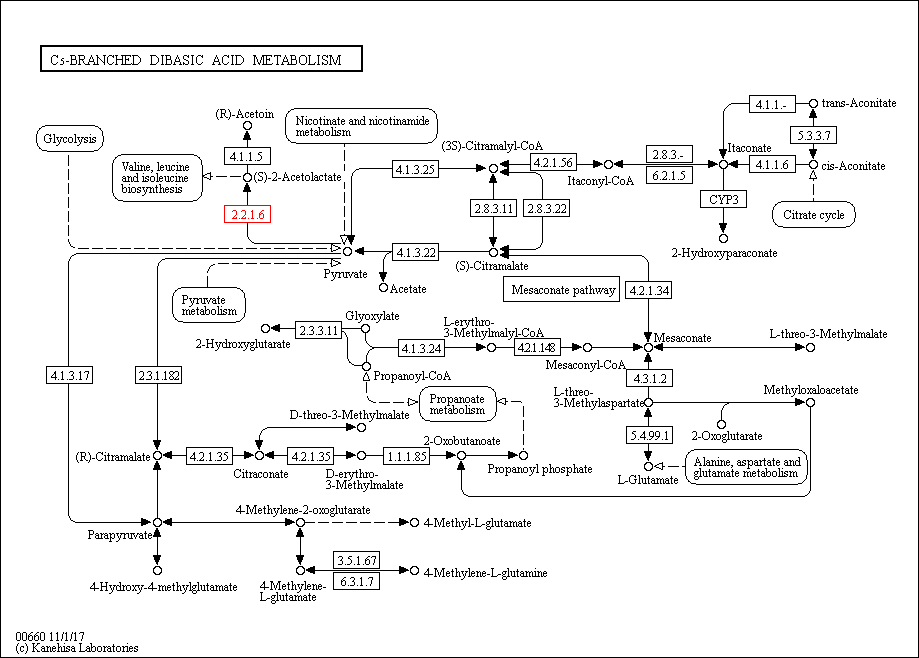


**B**

**Supplementary Figure 6** Most significantly enriched metabolic pathways in maize seedling leaves under drought stress. **A** – Photosynthesis antenna proteins pathway enriched in tolerant inbred line YE8112. **B** – C5-Branched dibasic acid metabolism pathway in enriched in sensitive inbred line MO17.
