# Supplementary material for: Comparative Proteomic and Physiological Analyses of Two Divergent Maize Inbred Lines Provide More Insights into Drought-Stress Tolerance Mechanisms
Source: Int J Mol Sci. 2018 Oct 18;19(10):3225. doi: 10.3390/ijms19103225 (PMC6213998; doi:10.3390/ijms19103225)
Supplement: Supplementary file 1 [file ijms-19-03225-s001.zip › Supplementary Material/SUPPLEMENTARY TABLES/Supplementary Table 2. DAPs observed in YE8112 before and after drought treatment (TC_TD).docx]

**Supplementary Table 2.** DAPs observed in tolerant line YE8112 before and after drought treatment (TD_TC)

| No. | Protein ID ^1^ | Gene name/ID ^2^ | Description ^3^ | Coverage (%)^4^ | Peptide  Fragments^5^ | Fold change ^6^ | p value ^7^ | Pathways ^8^ |
| --- | --- | --- | --- | --- | --- | --- | --- | --- |
| 1 | C0HJ06 |  | Uncharacterized protein | 22.4 | 1 | 1.37 | 0.0109 | MAPK signaling pathway/Plant hormone signaling |
| 2 | Q41746 | Lhcb5-1 | Chlorophyll a-b binding protein, chloroplastic | 55.8 | 10 | 1.24 | 0.0131 | Photosynthesis - antenna proteins |
| 3 | C0HGH7 | 100193714 | Universal stress family protein | 20.4 | 3 | 1.23 | 0.0430 |  |
| 4 | A0A1D6GAZ6 | ZEAMMB73_Zm00001d012677 | Glycerophosphodiester phosphodiesterase GDPD5 | 16.8 | 5 | 1.22 | 0.0136 | Glycerophospholipid metabolism |
| 5 | C0P948 |  | Uncharacterized protein | 55.9 | 20 | 1.21 | 0.0350 |  |
| 6 | A0A1D6PQ00 | 100286059 | U2 snRNP auxiliary factor large subunit | 9.4 | 2 | 0.83 | 0.0171 | Spliceosome |
| 7 | A0A1D6IUI1 | 100383306 | Ubiquitin carboxyl-terminal hydrolase 13 | 2.7 | 3 | 0.83 | 0.0217 |  |
| 8 | A0A1D6MJP2 | ZEAMMB73_Zm00001d039613 | Uncharacterized protein | 19.9 | 4 | 0.82 | 0.0111 |  |
| 9 | B4FTP2 | ZEAMMB73_Zm00001d021334 | Thioredoxin-like protein CDSP32 chloroplastic | 23.7 | 6 | 0.81 | 0.0246 |  |
| 10 | B4F845 | 100191245 | Uncharacterized protein | 3.0 | 1 | 0.81 | 0.0027 |  |
| 11 | H9BG22 | 101027254 | Alpha-dioxygenase | 4.4 | 3 | 0.80 | 0.0162 | alpha-linolenic acid metabolism |
| 12 | Q5GJ59 | TPS7 | Terpene synthase 7 | 14.8 | 5 | 0.78 | 0.0179 |  |
| 13 | C0PHF6 | 100383595 | AAA-ATPase ASD mitochondrial | 10.6 | 5 | 0.55 | 0.0487 |  |
| 14 | B6SQW8 |  | ^9^ Uncharacterized protein | 27.2 | 3 | 1.59 | 0.0155 |  |
| 15 | Q2XX23 | plt1 | Non-specific lipid-transfer protein | 53.6 | 4 | 1.53 | 0.0053 |  |
| 16 | B4FKG5 | 542304 | Abscisic acid stress ripening 1 | 47.1 | 4 | 1.34 | 0.0096 |  |
| 17 | B6UFE3 |  | Uncharacterized protein | 10.0 | 1 | 1.30 | 0.0436 |  |
| 18 | A0A1D6HWS1 | 100282063 | Dirigent protein | 34.3 | 4 | 1.29 | 0.0207 |  |
| 19 | B4FL55 | 542320 / Lhcb5-2 | Chlorophyll a-b binding protein, chloroplastic | 55.8 | 10 | 1.28 | 0.0053 | Photosynthesis - antenna proteins |
| 20 | B4FIE9 | 100194360 | S-adenosylmethionine synthase | 52.3 | 14 | 0.83 | 0.0171 | Biosynthesis of amino acids/ Cysteine and methionine metabolism |
| 21 | A0A1D6GZE2 | 100272744 | Ribose-phosphate pyrophosphokinase | 5.4 | 1 | 0.82 | 0.0078 | Purine metabolism/ Carbon metabolism/ Biosynthesis of amino acids / Pentose phosphate pathway |
| 22 | B6TD62 | 100282951 | Membrane steroid-binding protein | 35.8 | 5 | 0.81 | 0.0223 |  |
| 23 | A0A1D6PT84 | 100382471 | Protein transport protein Sec24-like CEF | 3.5 | 2 | 0.81 | 0.0009 | Protein processing in endoplasmic reticulum |
| 24 | A0A1D6IYL9 | 103641146 | AICARFT/IMPCHase bienzyme family protein | 7.1 | 2 | 0.80 | 0.0111 | Purine metabolism |
| 25 | C4J0F8 |  | Uncharacterized protein | 32.5 | 4 | 0.80 | 0.0090 | Ribosome |
| 26 | Q84TC2 | BX6 | DIBOA-glucoside dioxygenase BX6 | 8.0 | 3 | 0.79 | 0.0123 | Benzoxazinoid biosynthesis |
| 27 | A0A1D6EE87 | ZEAMMB73_Zm00001d004154 | Cleavage and polyadenylation specificity factor subunit 1 | 2.3 | 1 | 0.79 | 0.0032 | mRNA surveillance |
| 28 | B7ZYV4 | 100193626 | Arginine decarboxylase | 59.5 | 29 | 0.79 | 0.0006 | Arginine and proline metabolism |
| 29 | C0PHL2 | ZEAMMB73_Zm00001d018627 | Monosaccharide transporter1 | 3.8 | 1 | 0.79 | 0.0051 |  |
| 30 | B6STB7 |  | Ferredoxin-6 | 6.4 | 1 | 0.78 | 0.0230 |  |
| 31 | B4FQR3 | AR4 | Aldose reductase | 8.5 | 2 | 0.78 | 0.0382 |  |
| 32 | B6U463 | ZEAMMB73_Zm00001d011079 | Subtilisin-chymotrypsin inhibitor-2A | 78.1 | 3 | 0.78 | 0.0294 |  |
| 33 | A0A1D6NE76 | ZEAMMB73_Zm00001d043674 | Cytidine deaminase | 13.3 | 2 | 0.75 | 0.0028 | Pyrimidine metabolism |
| 34 | C0HDZ4 | ZEAMMB73_Zm00001d009084 | SAM-dependent methyltransferase superfamily protein | 14.1 | 2 | 0.73 | 0.0218 |  |
| 35 | B4FHK4 | 100194135 | Natterin-4 | 47.9 | 16 | 0.68 | 0.0015 |  |
| 36 | B6T003 |  | Stem 28 kDa glycoprotein | 49.6 | 10 | 0.65 | 0.0044 |  |
| 37 | A0A1D6EEX1 | 103646784 | Natterin-4 | 40.3 | 11 | 0.61 | 0.0001 |  |

^1^Protein ID, unique protein identifying number in the UniProt database; ^2^Gene name; name or ID number of the corresponding gene of the identified differentially abundant protein as searched against the maize sequence database Gramene ([http://ensemble.gramene.org/Zea mays](http://ensemble.gramene.org/Zea%20mays)); ^3^Description, annotated biological functions based on Gene Ontology (GO) analysis; ^4^ Coverage (%), sequence coverage is calculated as the number of amino acids in the peptide fragments observed divided by the protein amino acid length; ^5^ Peptides fragments, refer to the number of matched peptide fragments generated by trypsin digestion; ^6^ Fold change, is expressed as the ratio of intensities of up-regulated or down-regulated proteins between drought stress treatments and control (well-watered conditions); All the fold change figures below 1 represents that the proteins were down-regulated. All the figures above 1 means the proteins were up-regulated; ^7^ *p* value, statistical level (using Student`s *t*-test) below < 0.05, at which protein differential expression was accepted as significant; ^8^Pathways, metabolic KEGG pathways in which the identified protein was found to be significantly enriched; ^9^ uncharacterized protein, a protein without any functional annotations ascribed to it at the present.
