# Supplementary material for: Comparative Proteomic and Physiological Analyses of Two Divergent Maize Inbred Lines Provide More Insights into Drought-Stress Tolerance Mechanisms
Source: Int J Mol Sci. 2018 Oct 18;19(10):3225. doi: 10.3390/ijms19103225 (PMC6213998; doi:10.3390/ijms19103225)
Supplement: Supplementary file 1 [file ijms-19-03225-s001.zip › Supplementary Material/SUPPLEMENTARY TABLES/Supplementary Table 3 DAPs observed in sensitive line MO17 before and after drought treatment (SC_SD).docx]

**Supplementary Table 3.** DAPs observed in sensitive line MO17 before and after drought treatment (SD_SC)

| No. | Accession | Description | Coverage (%) | Peptide  fragments | | Fold change | p value |
| --- | --- | --- | --- | --- | --- | --- | --- |
| 1 | C0PHL2 | Monosaccharide transporter1 | 3.8 | 1 | 1.69 | | 0.0495 |
| 2 | B4FV94 | Chlorophyll a-b binding protein, chloroplastic | 49.8 | 7 | 1.66 | | 0.0326 |
| 3 | A0A1D6L5A0 | Alpha-1,4 glucan phosphorylase | 6.5 | 3 | 1.61 | | 0.0127 |
| 4 | B6SLG6 | Lil3 protein | 9.6 | 2 | 1.57 | | 0.0364 |
| 5 | C0HDZ4 | S-adenosyl-L-methionine-dependent methyltransferase superfamily protein | 14.1 | 2 | 1.52 | | 0.0245 |
| 6 | B6TD62 | Membrane steroid-binding protein 1 | 35.8 | 5 | 1.50 | | 0.0142 |
| 7 | B4FCG6 | Uncharacterized protein | 9.0 | 1 | 1.48 | | 0.0036 |
| 8 | S5TDC1 | Chlorophyll a-b binding protein, chloroplastic (Fragment) | 32.9 | 2 | 1.47 | | 0.0214 |
| 9 | B6SUJ9 | Oxygen evolving enhancer protein 3 | 25.1 | 4 | 1.47 | | 0.0267 |
| 10 | B4FTN5 | Metal-dependent protein hydrolase | 5.7 | 1 | 1.45 | | 0.0459 |
| 11 | B4FU01 | Cystathionine beta-lyase chloroplastic | 11.3 | 4 | 1.39 | | 0.0386 |
| 12 | A0A1D6KWT9 | Glycerophosphoryl diester phosphodiesterase | 7.6 | 2 | 1.37 | | 0.0024 |
| 13 | B6TGS8 | 6,7-dimethyl-8-ribityllumazine synthase | 26.1 | 3 | 1.35 | | 0.0253 |
| 14 | B8A3B7 | Uncharacterized protein | 20.8 | 3 | 1.33 | | 0.0278 |
| 15 | C0P6L9 | Uncharacterized protein | 40.2 | 7 | 1.33 | | 0.0011 |
| 16 | A0A1D6F5Z1 | Succinate dehydrogenase assembly factor 2 mitochondrial | 10.1 | 1 | 1.33 | | 0.0220 |
| 17 | B4FLE3 | HSP20-like chaperones superfamily protein | 33.0 | 4 | 1.32 | | 0.0484 |
| 18 | B6U3Z0 | 50S ribosomal protein L21 | 42.5 | 7 | 1.31 | | 0.0149 |
| 19 | A0A1D6HG23 | Uncharacterized protein | 5.9 | 1 | 1.31 | | 0.0306 |
| 20 | A0A1D6MUF1 | Heat shock 70 kDa protein 6 chloroplastic | 55.1 | 29 | 1.31 | | 0.0476 |
| 21 | K7TP80 | Zinc finger (C3HC4-type RING finger) family protein | 36.0 | 14 | 1.31 | | 0.0028 |
| 22 | A0A1D6JW44 | Calcium-binding EF-hand family protein | 9.0 | 1 | 1.30 | | 0.0014 |
| 23 | B6U284 | 14-3-3-like protein | 64.1 | 14 | 1.30 | | 0.0006 |
| 24 | B4F925 | Superoxide dismutase | 46.0 | 9 | 1.30 | | 0.0071 |
| 25 | A0A097PND9 | AT5G11810-like protein (Fragment) ] | 6.9 | 1 | 1.29 | | 0.0358 |
| 26 | B4FE30 | 10 kDa chaperonin | 45.9 | 5 | 1.29 | | 0.0024 |
| 27 | B4FAV3 | 40S ribosomal protein S3a | 49.2 | 11 | 1.29 | | 0.0117 |
| 28 | C4J0W2 | Uncharacterized protein | 17.9 | 3 | 1.29 | | 0.0077 |
| 29 | A0A1D6F4D3 | Uncharacterized protein | 23.5 | 1 | 1.29 | | 0.0443 |
| 30 | C5IHD6 | Pyruvate, phosphate dikinase | 66.2 | 44 | 1.29 | | 0.0231 |
| 31 | B4FBV8 | Eukaryotic translation initiation factor 3 subunit G | 10.1 | 2 | 1.29 | | 0.0390 |
| 32 | B4FZU8 | Malate dehydrogenase | 56.8 | 12 | 1.28 | | 0.0150 |
| 33 | Q4A1J8 | Cysteine proteinase inhibitor (Fragment) | 11.3 | 1 | 1.28 | | 0.0293 |
| 34 | A0A1X7YHJ3 | Photosystem II CP47 reaction center protein | 46.9 | 16 | 1.28 | | 0.0062 |
| 35 | B4FWP6 | Uncharacterized protein | 9.9 | 4 | 1.27 | | 0.0374 |
| 36 | B4FTL2 | Protein TIC 22 chloroplastic | 9.3 | 2 | 1.27 | | 0.0001 |
| 37 | C0P8X5 | Electron transfer flavoprotein subunit beta mitochondrial | 14.9 | 1 | 1.25 | | 0.0020 |
| 38 | A0A1D6JFG3 | Beta-D-xylosidase 4 | 16.6 | 5 | 1.25 | | 0.0107 |
| 39 | A0A1D6HE45 | ATP-dependent Clp protease proteolytic subunit | 33.7 | 5 | 1.25 | | 0.0218 |
| 40 | Q2XX37 | Non-specific lipid-transfer protein | 46.2 | 4 | 1.25 | | 0.0435 |
| 41 | B8A3M2 | Uncharacterized protein | 18.2 | 1 | 1.24 | | 0.0357 |
| 42 | A0A1D6JYF7 | Kinesin-like protein | 3.1 | 1 | 1.24 | | 0.0409 |
| 43 | A0A1D6E501 | 3-isopropylmalate dehydrogenase | 50.1 | 12 | 1.24 | | 0.0449 |
| 44 | A0A1D6L0Y0 | Uncharacterized protein | 7.6 | 1 | 1.24 | | 0.0111 |
| 45 | B4FJM6 | Lactoylglutathione lyase / glyoxalase I family protein | 66.9 | 6 | 1.23 | | 0.0113 |
| 46 | A0A096PRE6 | Fibrillin1 | 31.4 | 9 | 1.23 | | 0.0421 |
| 47 | K7UWX4 | GrpE protein homolog | 44.2 | 11 | 1.23 | | 0.0083 |
| 48 | A0A1D6JZL1 | Thioredoxin superfamily protein | 17.0 | 1 | 1.23 | | 0.0425 |
| 49 | B4FMA5 | Chaperone DnaJ-domain superfamily protein | 14.6 | 2 | 1.23 | | 0.0378 |
| 50 | B6TNV8 | Shikimate kinase family protein | 34.8 | 6 | 1.22 | | 0.0084 |
| 51 | B6TUR3 | Uncharacterized protein | 26.3 | 1 | 1.22 | | 0.0132 |
| 52 | B7ZZT1 | Uncharacterized protein | 6.5 | 1 | 1.22 | | 0.0039 |
| 53 | Q6VWJ0 | Caffeoyl-CoA 3-O-methyltransferase 1 | 12.4 | 2 | 1.22 | | 0.0210 |
| 54 | B8A045 | Phospholipase D | 2.9 | 2 | 1.22 | | 0.0211 |
| 55 | B6TGF1 | Malate dehydrogenase | 72.4 | 14 | 1.22 | | 0.0092 |
| 56 | A0A1D6FI49 | TPR repeat | 6.0 | 1 | 1.22 | | 0.0283 |
| 57 | B6UHD9 | Peptide chain release factor 2 | 8.0 | 2 | 1.22 | | 0.0374 |
| 58 | B6TDF7 | Plastid-specific 30S ribosomal protein 2 | 45.4 | 9 | 1.21 | | 0.0014 |
| 59 | Q1KKB7 | NADH-ubiquinone oxidoreductase chain 1 | 5.9 | 1 | 1.21 | | 0.0355 |
| 60 | P08440 | Fructose-bisphosphate aldolase, cytoplasmic isozyme | 54.7 | 17 | 1.20 | | 0.0240 |
| 61 | B4FUC4 | Plasma membrane-associated cation-binding protein 1 | 24.7 | 3 | 1.20 | | 0.0030 |
| 62 | B6SQL7 | Uncharacterized protein | 11.4 | 1 | 1.20 | | 0.0021 |
| 63 | A0A059Q7D4 | Photosystem II D2 protein | 25.2 | 7 | 1.20 | | 0.0058 |
| 64 | C4J3Q4 | YCF37-like protein | 17.7 | 2 | 1.20 | | 0.0017 |
| 65 | B4FTK9 | Alpha/beta-Hydrolases superfamily protein | 33.6 | 6 | 1.20 | | 0.0421 |
| 66 | B6TBW4 | ERBB-3 BINDING PROTEIN 1 | 30.5 | 10 | 0.83 | | 0.0174 |
| 67 | A0A1D6DVJ8 | H(+)-ATPase 5 | 34.6 | 18 | 0.83 | | 0.0322 |
| 68 | A0A1D6DYT2 | Signal recognition particle 14 kDa protein | 11.3 | 1 | 0.83 | | 0.0172 |
| 69 | B6T346 | THO complex subunit 4 | 14.2 | 3 | 0.83 | | 0.0390 |
| 70 | A0A1D6GKY6 | Uncharacterized protein | 4.9 | 1 | 0.83 | | 0.0411 |
| 71 | B4FKM0 | Uncharacterized protein | 53.7 | 17 | 0.83 | | 0.0062 |
| 72 | B6SJ21 | Guanine nucleotide-binding protein beta subunit-like protein | 59.3 | 13 | 0.83 | | 0.0232 |
| 73 | B4FMM1 | Putative carboxylesterase 2 | 7.9 | 2 | 0.82 | | 0.0186 |
| 74 | A0A1D6NHS9 | Glycosyltransferase | 4.4 | 2 | 0.82 | | 0.0342 |
| 75 | C0PI72 | Uncharacterized protein | 8.3 | 1 | 0.82 | | 0.0201 |
| 76 | C0HI59 | Uncharacterized protein | 13.3 | 5 | 0.82 | | 0.0181 |
| 77 | A0A1D6M4E1 | Glutathione transferase41 | 8.6 | 1 | 0.82 | | 0.0025 |
| 78 | A0A1D6GZE2 | Ribose-phosphate pyrophosphokinase | 5.4 | 1 | 0.82 | | 0.0068 |
| 79 | B6TU01 | Uncharacterized protein | 5.1 | 1 | 0.82 | | 0.0302 |
| 80 | A0A1D6M0I9 | Carboxypeptidase | 18.5 | 7 | 0.82 | | 0.0435 |
| 81 | A0A1D6GES6 | DNA gyrase subunit A chloroplastic/mitochondrial | 1.9 | 1 | 0.81 | | 0.0372 |
| 82 | B6TIL4 | GDP-mannose 3,5-epimerase 2 | 20.5 | 6 | 0.81 | | 0.0265 |
| 83 | B6T3J2 | Eukaryotic translation initiation factor 2 beta subunit | 12.9 | 3 | 0.81 | | 0.0206 |
| 84 | C4J0F8 | Uncharacterized protein | 32.5 | 4 | 0.81 | | 0.0465 |
| 85 | A0A1D6F8L4 | Coatomer subunit gamma | 7.2 | 4 | 0.81 | | 0.0316 |
| 86 | C0PI69 | Uncharacterized protein | 18.5 | 2 | 0.81 | | 0.0092 |
| 87 | A0A1D6KJI4 | Pyridoxamine 5'-phosphate oxidase family protein | 6.6 | 1 | 0.81 | | 0.0086 |
| 88 | A0A0B4J3C2 | Elongation factor 1-alpha | 42.1 | 15 | 0.81 | | 0.0463 |
| 89 | B4FEV5 | Uncharacterized protein | 13.8 | 1 | 0.81 | | 0.0400 |
| 90 | P26566 | 50S ribosomal protein L20, chloroplastic | 20.2 | 3 | 0.81 | | 0.0476 |
| 91 | A0A1D6KBW7 | Hsp20/alpha crystallin family protein | 17.8 | 2 | 0.81 | | 0.0098 |
| 92 | A0A1D6EIR5 | Acyl-CoA synthetase long-chain family member 3 | 2.6 | 2 | 0.81 | | 0.0031 |
| 93 | A0A1D6IL69 | Peptide deformylase | 20.4 | 2 | 0.80 | | 0.0142 |
| 94 | A0A1D6ICZ3 | Calcium dependent protein kinase8 | 7.0 | 3 | 0.80 | | 0.0465 |
| 95 | A0A1D6K128 | Alpha-L-arabinofuranosidase 1 | 7.5 | 4 | 0.80 | | 0.0110 |
| 96 | B4FAJ4 | Uncharacterized protein | 2.8 | 1 | 0.80 | | 0.0260 |
| 97 | B6T9T5 | Uncharacterized protein | 4.3 | 1 | 0.80 | | 0.0002 |
| 98 | Q9M7E2 | Elongation factor 1-alpha | 30.7 | 10 | 0.80 | | 0.0134 |
| 99 | B7ZZ42 | Heat shock 70 kDa protein 3 | 58.6 | 30 | 0.80 | | 0.0076 |
| 100 | M1PUC2 | Gamma-tocopherol methyltransferase (Fragment) | 17.2 | 2 | 0.80 | | 0.0017 |
| 101 | B6SUQ3 | Uncharacterized protein | 20.2 | 4 | 0.79 | | 0.0463 |
| 102 | A0A1D6N9X4 | Insulin-degrading enzyme-like 1 peroxisomal | 3.5 | 3 | 0.79 | | 0.0149 |
| 103 | A0A1D6IHP2 | ARM repeat superfamily protein | 6.5 | 5 | 0.79 | | 0.0161 |
| 104 | A0A1D6MT58 | NAD(P)-binding Rossmann-fold superfamily protein | 13.4 | 2 | 0.79 | | 0.0002 |
| 105 | A0A1D6MPP0 | Importin subunit alpha | 18.5 | 5 | 0.79 | | 0.0298 |
| 106 | B4FLV6 | Protein translation factor SUI1 | 20.0 | 3 | 0.79 | | 0.0269 |
| 107 | B4FQM2 | Pyrophosphate--fructose 6-phosphate 1-phosphotransferase subunit beta | 6.7 | 2 | 0.79 | | 0.0123 |
| 108 | B4FQP6 | Mannose-1-phosphate guanylyltransferase 1 | 8.0 | 3 | 0.79 | | 0.0291 |
| 109 | B6TP02 | Aspartic proteinase nepenthesin-1 | 5.6 | 2 | 0.78 | | 0.0276 |
| 110 | A0A1D6PW61 | DNA topoisomerase 1 beta | 3.1 | 1 | 0.78 | | 0.0189 |
| 111 | A0A1D6MME0 | Actin-interacting protein 1-2 | 10.1 | 3 | 0.78 | | 0.0260 |
| 112 | B6SR37 | Uncharacterized protein | 17.3 | 2 | 0.78 | | 0.0070 |
| 113 | A0A1D6JQY8 | Uroporphyrinogen-III synthase chloroplastic | 2.8 | 1 | 0.78 | | 0.0294 |
| 114 | B4FVS0 | Serine/threonine-protein phosphatase | 9.2 | 3 | 0.78 | | 0.0008 |
| 115 | A0A1D6IIC2 | Nuclear transport factor 2 (NTF2) family protein with RNA binding (RRM-RBD-RNP motifs) domain | 5.6 | 1 | 0.77 | | 0.0092 |
| 116 | B6U4J6 | Embryogenesis transmembrane protein | 4.5 | 1 | 0.77 | | 0.0258 |
| 117 | B4FFV2 | SWIB/MDM2 domain superfamily protein | 5.2 | 1 | 0.77 | | 0.0053 |
| 118 | C0P626 | Carbonic anhydrase | 74.3 | 13 | 0.77 | | 0.0272 |
| 119 | C0PIS3 | Uncharacterized protein | 30.6 | 4 | 0.77 | | 0.0408 |
| 120 | Q9M7E3 | Elongation factor 1-alpha | 37.8 | 13 | 0.76 | | 0.0045 |
| 121 | B6SI29 | Histone H2A | 29.3 | 4 | 0.76 | | 0.0326 |
| 122 | B4FIA6 | Histone H2A | 28.9 | 3 | 0.76 | | 0.0406 |
| 123 | A0A1D6F2F0 | Synaptotagmin-5 | 4.6 | 1 | 0.76 | | 0.0214 |
| 124 | A0A1D6DSQ0 | CASP-like protein 1 | 3.1 | 1 | 0.75 | | 0.0074 |
| 125 | A0A1D6JVL9 | Small nuclear ribonucleoprotein Sm D3 | 21.7 | 2 | 0.75 | | 0.0384 |
| 126 | A0A1D6GQM8 | Acylamino-acid-releasing enzyme | 5.5 | 1 | 0.74 | | 0.0203 |
| 127 | B6SLI1 | 40S ribosomal protein S30 | 16.1 | 1 | 0.74 | | 0.0110 |
| 128 | B6T8R5 | 60S ribosomal protein L34 | 41.2 | 5 | 0.74 | | 0.0129 |
| 129 | A0A1D6K8X4 | Haloacid dehalogenase-like hydrolase domain-containing protein Sgpp | 19.3 | 3 | 0.74 | | 0.0035 |
| 130 | A0A1D6GTD7 | 26S proteasome non-ATPase regulatory subunit 13 homolog A | 6.8 | 2 | 0.74 | | 0.0004 |
| 131 | B6U4G3 | Acetolactate synthase | 1.8 | 1 | 0.73 | | 0.0066 |
| 132 | B6SHX9 | Histone H2A | 29.5 | 4 | 0.73 | | 0.0311 |
| 133 | B4FMB1 | DUF538 family protein | 32.9 | 3 | 0.73 | | 0.0104 |
| 134 | A0A1D6LBT4 | Protein prenylyltransferase superfamily protein | 7.0 | 1 | 0.72 | | 0.0475 |
| 135 | C0PMB3 | Uncharacterized protein | 2.1 | 1 | 0.72 | | 0.0136 |
| 136 | A0A1D6P0E7 | Triose phosphate/phosphate translocator TPT chloroplastic | 22.1 | 2 | 0.72 | | 0.0111 |
| 137 | B4F9V2 | VAP27 | 6.2 | 1 | 0.71 | | 0.0230 |
| 138 | B4FFS7 | Uncharacterized protein | 7.8 | 1 | 0.71 | | 0.0347 |
| 139 | A0A1D6K876 | 3-hydroxyisobutyryl-CoA hydrolase-like protein 2 mitochondrial | 12.8 | 1 | 0.71 | | 0.0141 |
| 140 | B4FQY7 | Uncharacterized protein | 3.9 | 1 | 0.71 | | 0.0409 |
| 141 | A0A1D6FPL0 | Fructose-16-bisphosphatase cytosolic | 21.2 | 8 | 0.70 | | 0.0179 |
| 142 | B4FH44 | Thioredoxin | 38.5 | 4 | 0.70 | | 0.0440 |
| 143 | Q8LLS4 | Phosphoglycerate kinase (Fragment) | 32.2 | 9 | 0.69 | | 0.0440 |
| 144 | B4FXX2 | 60S ribosomal protein L34 | 37.0 | 4 | 0.69 | | 0.0230 |
| 145 | A0A1D6H8W7 | Ubiquitin-activating enzyme E1 domain-containing protein 1 | 6.4 | 1 | 0.69 | | 0.0008 |
| 146 | A0A1D6H1R5 | V-type proton ATPase catalytic subunit A | 72.0 | 24 | 0.69 | | 0.0272 |
| 147 | A0A1D6K8W1 | Dynamin-related protein 1E | 2.7 | 1 | 0.68 | | 0.0411 |
| 148 | C0PAX9 | Uncharacterized protein | 12.5 | 1 | 0.67 | | 0.0112 |
| 149 | A0A1D6QSH1 | Cullin-associated NEDD8-dissociated protein 1 | 3.6 | 3 | 0.65 | | 0.0139 |
| 150 | B6TNP4 | Histone H1 | 41.0 | 11 | 0.65 | | 0.0485 |
| 151 | A0A1D6H070 | Leucine-rich repeat/extensin 2 | 2.4 | 1 | 0.64 | | 0.0293 |
| 152 | B6UHJ4 | Elongation factor 1-alpha | 42.1 | 15 | 0.61 | | 0.0037 |
| 153 | C0PH85 | Tubulin beta chain | 15.8 | 6 | 0.61 | | 0.0027 |
| 154 | C0HH87 | Uncharacterized protein | 23.2 | 6 | 0.60 | | 0.0245 |
| 155 | A0A1D6N6L9 | Hevamine-A | 4.0 | 1 | 0.56 | | 0.0123 |
| 156 | A0A1D6MEZ2 | Serine/threonine-protein kinase AGC1-5 | 1.4 | 1 | 0.55 | | 0.0120 |
| 157 | E7DDW6 | Clathrin light chain | 23.0 | 4 | 0.52 | | 0.0203 |

**Note;** Accession, the unique identifying number in NCBI database; Coverage (%), sequence coverage is calculated as the number of amino acids in the peptide fragments observed divided by the protein amino acid length; Peptides fragments, refer to the number of matched peptide fragments generated by trypsin digestion; Fold change, is expressed as the ratio of intensities of up-regulated or down-regulated proteins between drought stress treatments and control (well-watered conditions); All the fold change figures below 1 represents that the proteins were down-regulated. All the figures above 1 means the proteins were up-regulated; uncharacterized protein, a protein without any functional annotations ascribed to it at the present; *p* value, statistical level (using Student`s *t*-test) below < 0.05, at which protein differential expression was accepted as significant.
