# Supplementary material for: Comparative Proteomic and Physiological Analyses of Two Divergent Maize Inbred Lines Provide More Insights into Drought-Stress Tolerance Mechanisms
Source: Int J Mol Sci. 2018 Oct 18;19(10):3225. doi: 10.3390/ijms19103225 (PMC6213998; doi:10.3390/ijms19103225)
Supplement: Supplementary file 1 [file ijms-19-03225-s001.zip › Supplementary Material/SUPPLEMENTARY TABLES/Supplementary Table 4 SD_TD specific DAPs.docx]

**Supplementary Table 4** Specific DAPs shared between the drought sensitive and drought tolerant lines after drought treatment (Specific DAPs of SD_TD)

| No. | Protein ID^1^ | Gene name/ID^2^ | Description ^3^ | Coverage (%)^4^ | Peptide Fragments ^5^ | Ratio (SD/TD) ^6^ | p value^7^ | Pathway^8^ |
| --- | --- | --- | --- | --- | --- | --- | --- | --- |
| 1 | B6TQA4 | 100277165 | Uncharacterized protein ^9^ | 15.7 | 1 | 3.68 | 0.0082 |  |
| 2 | B6UHR7 | 100286323 | Acetyltransferase, GNAT family protein | 8.6 | 2 | 1.82 | 0.0373 |  |
| 3 | B4FSZ7 | 100856885 | Putative mitochondrial-processing peptidase subunit alpha-2 chloroplastic | 34.5 | 11 | 1.55 | 0.0222 |  |
| 4 | A0A1D6NKY3 | 100283248 | Cytokinin riboside 5'-monophosphate phosphoribohydrolase | 7.4 | 1 | 1.55 | 0.0442 |  |
| 5 | B4FUG3 | MSRB3 | Methionine sulfoxide reductase | 36.2 | 5 | 1.49 | 0.0239 |  |
| 6 | B6T834 |  | 40S ribosomal protein S3a | 49.2 | 11 | 1.42 | 0.0446 | Ribosome |
| 7 | A0A1D6JN63 | ZEAMMB73_Zm00001d027578 | Outer mitochondrial membrane porin1 | 3.9 | 1 | 1.37 | 0.0233 |  |
| 8 | A0A1D6IP54 | 100501403 | Chlorophyll a-b binding protein, chloroplastic | 32.9 | 2 | 1.36 | 0.0379 | Proteasome |
| 9 | K7U2X9 | 103653897 | Bowman-Birk type trypsin inhibitor | 15.7 | 1 | 1.35 | 0.0175 | Terpenoid backbone biosynthesis |
| 10 | K7TT73 | 100274348 | Heterodimeric geranylgeranyl pyrophosphate synthase small subunit chloroplastic | 25.7 | 6 | 1.35 | 0.0449 | Ribosome |
| 11 | B6TW28 |  | 40S ribosomal protein S3-1 | 46.5 | 8 | 1.35 | 0.0499 |  |
| 12 | A0A1D6JKM0 | 100216768 | Pyridoxamine 5'-phosphate oxidase family protein | 6.6 | 1 | 1.34 | 0.0131 |  |
| 13 | C0P472 | 100382079 | Ferredoxin-thioredoxin reductase, variable chain | 39.2 | 5 | 1.34 | 0.0300 |  |
| 14 | K7UU30 | 103635449 | Membrane steroid-binding protein 1 | 35.8 | 5 | 1.33 | 0.0094 | Protein processing in endoplasmic reticulum |
| 15 | B6TNN9 |  | Adenylosuccinate lyase | 4.4 | 2 | 1.31 | 0.0011 |  |
| 16 | B8A2G7 | 100280198 | DnaJ protein ERDJ3B | 6.6 | 2 | 1.31 | 0.0166 |  |
| 17 | B6SXJ2 |  | Cystathionine beta-lyase chloroplastic | 11.3 | 4 | 1.31 | 0.0448 |  |
| 18 | B6SJJ1 | 100286120 | Cyclase | 24.0 | 3 | 1.31 | 0.0011 | Ribosome |
| 19 | B6TZU0 |  | 60S ribosomal protein L31 | 50.4 | 6 | 1.29 | 0.0434 |  |
| 20 | C3UZ63 | HSP90-2 | Uncharacterized protein | 25.0 | 2 | 1.28 | 0.0450 | Plant pathogen interaction / Protein processing in endoplasmic reticulum |
| 21 | B4G1V3 |  | Glutathione S-transferase GSTU6 | 13.4 | 2 | 1.27 | 0.0195 |  |
| 22 | A0A1D6EI90 | ZEAMMB73_Zm00001d004942 | Chloroplastic quinone-oxidoreductase | 16.2 | 4 | 1.27 | 0.0280 |  |
| 23 | A0A1D6K5D2 | ZEAMMB73_Zm00001d029457 | Thioredoxin-like 4 | 14.7 | 2 | 1.27 | 0.0169 |  |
| 24 | A0A1D6HFD3 | 103627523 | Chaperone protein ClpB3 chloroplastic | 25.2 | 18 | 1.26 | 0.0009 |  |
| 25 | B4FVB8 |  | HSP protein | 49.2 | 32 | 1.26 | 0.0087 |  |
| 26 | B6T6N9 |  | Alpha/beta-Hydrolases superfamily protein | 8.6 | 2 | 1.26 | 0.0105 |  |
| 27 | A0A1D6IKH4 | 107305678 | Nucleoredoxin1 | 18.1 | 7 | 1.23 | 0.0143 | Carbon metabolism/ Citrate cycle (TCA cycle)/ Cysteine and methionine metabolism/ Pyruvate metabolism |
| 28 | A0A1D6F9C0 | ZEAMMB73_Zm00001d007854 | Tripeptidyl-peptidase 2 | 20.1 | 17 | 1.23 | 0.0447 |  |
| 29 | A0A1D6JSN5 | 100285961 | Uncharacterized protein | 20.3 | 8 | 1.23 | 0.0277 |  |
| 30 | B6TMN2 |  | CREG1 protein | 7.1 | 1 | 1.23 | 0.0010 |  |
| 31 | B4FTY9 |  | Putative mitochondrial-processing peptidase subunit beta mitochondrial | 27.1 | 4 | 1.23 | 0.0225 |  |
| 32 | B6SR25 |  | Uncharacterized protein | 7.1 | 1 | 1.23 | 0.0492 |  |
| 33 | B4FZ14 | 100274094 | T-complex protein 1 subunit gamma | 6.3 | 2 | 1.23 | 0.0281 |  |
| 34 | B6SI11 | 100280540 | Multisynthetase complex auxiliary component p43 | 31.1 | 6 | 1.23 | 0.0347 |  |
| 35 | B6T2Y1 |  | Beta-D-xylosidase 4 | 16.6 | 5 | 1.23 | 0.0244 |  |
| 36 | C0PP27 |  | Transcription regulator | 11.0 | 1 | 1.22 | 0.0429 | Phosphatidylinositol signaling system / Inositol phosphate metabolism |
| 37 | A0A1D6G0C8 | ZEAMMB73_Zm00001d011448 | Uncharacterized protein | 17.0 | 2 | 1.22 | 0.0143 |  |
| 38 | A0A1D6KFE6 | ZEAMMB73_Zm00001d030968 | Peroxiredoxin-5 | 67.0 | 11 | 1.22 | 0.0046 |  |
| 39 | B6U471 | 100285364 | Outer mitochondrial membrane protein porin | 42.9 | 9 | 1.22 | 0.0302 |  |
| 40 | B6U151 | GATA | Uncharacterized protein | 9.9 | 3 | 1.21 | 0.0399 | Aminoacyl-tRNA biosynthesis |
| 41 | B6TXZ8 | 100284842 | Inositol-1-monophosphatase | 15.3 | 3 | 1.21 | 0.0318 | Carbon fixation in photosynthetic organisms/ Carbon metabolism/ Amino acids biosynthesis |
| 42 | A0A1D6JYD5 | 100383335 | Polygalacturonase inhibitor 1 | 12.3 | 2 | 1.21 | 0.0189 | Arginine biosynthesis/ 2-Oxocarboxylic acid metabolism/ Biosynthesis of amino acids |
| 43 | B4FYZ0 | 100274083 | 14-3-3-like protein | 64.1 | 14 | 1.21 | 0.0020 |  |
| 44 | C0PFY0 | ZEAMMB73_Zm00001d002969 | Fructose-bisphosphate aldolase, cytoplasmic isozyme | 54.7 | 17 | 1.21 | 0.0346 |  |
| 45 | C0PBS1 | 100501088 | Ribonucleoprotein A | 32.1 | 4 | 1.20 | 0.0190 | Indole alkaloid biosynthesis |
| 46 | B4FHT1 | Ann6 | Glutamyl-tRNA(Gln) amidotransferase subunit A, chloroplastic/mitochondrial | 21.0 | 8 | 1.20 | 0.0221 |  |
| 47 | B6SUD1 | ZEAMMB73_Zm00001d049059 | Arginine biosynthesis bifunctional protein ArgJ, chloroplastic | 11.8 | 4 | 0.83 | 0.0496 | Fatty acid degradation/ Tyrosine metabolism/ Glycolysis / alpha-Linolenic acid metabolism |
| 48 | B6TG61 | 100283242 | Beta-D-glucosidase | 48.3 | 22 | 0.83 | 0.0023 | Ribosome |
| 49 | B6SHW0 | 103653691 | Histone H2B | 59.5 | 11 | 0.83 | 0.0125 | Ribosome |
| 50 | C0HFI7 |  | 26S proteasome non-ATPase regulatory subunit 13 homolog A | 6.8 | 2 | 0.83 | 0.0221 |  |
| 51 | A0A1D6L787 | 103644157 | Annexin | 26.8 | 7 | 0.83 | 0.0340 | Tryptophan metabolism |
| 52 | C0PAR7 |  | Importin subunit alpha | 18.5 | 5 | 0.83 | 0.0441 | Oxidative phosphorylation |
| 53 | B4FTT2 | 100273233 | Alcohol dehydrogenase 2 | 28.0 | 8 | 0.83 | 0.0364 |  |
| 54 | A0A1D6ICV7 | ZEAMMB73_Zm00001d021615 | 60S ribosomal protein L6 | 50.2 | 12 | 0.83 | 0.0065 |  |
| 55 | B4FBD6 | 100192110 | Uncharacterized protein | 7.2 | 2 | 0.82 | 0.0054 |  |
| 56 | B6TJ90 |  | Peptide deformylase | 20.4 | 2 | 0.82 | 0.0172 |  |
| 57 | B6SZ64 |  | Acyl-CoA synthetase long-chain family member 3 | 2.6 | 2 | 0.82 | 0.0047 | Ribosome |
| 58 | B6TKC8 | 100283652 | Alpha-L-arabinofuranosidase 1 | 7.5 | 4 | 0.82 | 0.0105 |  |
| 59 | A0A1D6K8Y4 | 100284460 | CASP-like protein 1 | 3.1 | 1 | 0.81 | 0.0383 |  |
| 60 | B6TBN6 |  | DUF538 family protein | 32.9 | 3 | 0.81 | 0.0406 | Pyruvate metabolism/ Carbon metabolism/ Purine metabolism/ Amino acids Biosynthesis |
| 61 | A0A0D5BY62 | MSRB1 | Apyrase 1 | 17.1 | 6 | 0.81 | 0.0114 |  |
| 62 | A0A1D6GWE1 | 100279926 | 60S ribosomal protein L34 | 41.2 | 5 | 0.81 | 0.0236 | Plant-pathogen interaction/ Protein processing in endoplasmic reticulum |
| 63 | C4J3C3 |  | 60S ribosomal protein L35 | 26.8 | 4 | 0.81 | 0.0369 |  |
| 64 | B4FKD4 | 100283662 | Actin-interacting protein 1-2 | 10.1 | 3 | 0.81 | 0.0223 | Glycerophospholipid metabolism |
| 65 | B4FGJ4 | 732791 | Methionine sulfoxide reductase | 26.3 | 4 | 0.81 | 0.0349 | Pyruvate metabolism/ Glycolysis/ Citrate cycle (TCA cycle)/ Carbon metabolism |
| 66 | B6TRA8 |  | Shepherd-like1 | 38.7 | 28 | 0.81 | 0.0229 |  |
| 67 | B4FAB2 | 100272787 | Uncharacterized protein | 8.5 | 2 | 0.81 | 0.0292 |  |
| 68 | A0A1D6MDL2 | 100281932 | Acyl-protein thioesterase 2 | 5.9 | 2 | 0.81 | 0.0168 | Ribosome |
| 69 | B6TFA0 |  | 2-hydroxy-3-oxopropionate reductase | 58.0 | 10 | 0.80 | 0.0273 |  |
| 70 | A0A1D6KFD0 | 100272440 | Protein transport protein Sec24-like CEF | 3.5 | 2 | 0.80 | 0.0019 |  |
| 71 | B4FCE3 | 100192719 | AAA-ATPase ASD mitochondrial | 29.5 | 11 | 0.80 | 0.0449 | RNA degradation/Spliceosome |
| 72 | A0A1D6I602 | ZEAMMB73_Zm00001d020740 | Putative carboxylesterase 2 | 13.5 | 2 | 0.80 | 0.0377 |  |
| 73 | A0A1D6DSQ7 | 100194172 | Pyruvate dehydrogenase E1 component subunit alpha | 26.7 | 7 | 0.80 | 0.0476 |  |
| 74 | B4FMZ0 |  | NADH-cytochrome b5 reductase | 16.5 | 3 | 0.80 | 0.0323 |  |
| 75 | A0A1D6HLS2 | ZEAMMB73_Zm00001d018238 | Histone H1 | 17.5 | 3 | 0.80 | 0.0320 |  |
| 76 | K7TH21 | ZEAMMB73_Zm00001d024314 | Molecular chaperone Hsp40/DnaJ family protein | 19.5 | 7 | 0.80 | 0.0043 | Phenylpropanoid biosynthesis |
| 77 | A0A1D6IBA6 | 100276916 | 40S ribosomal protein S12 | 53.3 | 5 | 0.80 | 0.0500 |  |
| 78 | B6SGJ5 | 100280465 | D-amino acid oxidase | 16.8 | 5 | 0.80 | 0.0146 |  |
| 79 | A0A1D6QRC8 | 100192485 | FAD/NAD(P)-binding oxidoreductase family protein | 10.6 | 3 | 0.80 | 0.0314 | Tyrosine metabolism |
| 80 | B6SKW0 |  | Sm-like protein LSM6A | 7.4 | 1 | 0.80 | 0.0268 |  |
| 81 | B4FSW0 | 100276789 | AIG2-like (Avirulence induced gene) family protein | 10.2 | 1 | 0.79 | 0.0113 | Ribosome |
| 82 | B6UFX4 |  | Acetolactate synthase | 1.8 | 1 | 0.79 | 0.0022 |  |
| 83 | C0PBL7 |  | Ectonucleotide pyrophosphatase/phosphodiesterase 1 | 7.7 | 2 | 0.79 | 0.0126 | Peroxisome |
| 84 | A0A1D6PWC2 | ZEAMMB73_Zm00001d049597 | Carboxypeptidase | 18.5 | 7 | 0.77 | 0.0202 |  |
| 85 | A0A1D6HAP2 | 100285176 | Putative carboxylesterase 2 | 7.9 | 2 | 0.77 | 0.0101 |  |
| 86 | A0A1D6N9T2 | ZEAMMB73_Zm00001d043259 | Pop3 peptide | 23.4 | 2 | 0.76 | 0.0369 | Porphyrin and chlorophyll metabolism |
| 87 | A0A1D6EWJ3 | 100272786 | Putative leucine-rich repeat receptor-like protein kinase family protein | 11.5 | 8 | 0.75 | 0.0061 |  |
| 88 | B6SMC7 | 100282513 | Glutathione reductase cytosolic | 32.9 | 12 | 0.75 | 0.0159 | Ribosome |
| 89 | B8A367 | 100280321 | Stem 28 kDa glycoprotein | 15.7 | 4 | 0.75 | 0.0457 | Carbon metabolism/ Sulfur metabolism/ Cysteine and methionine metabolism |
| 90 | B6SZK3 | 100281854 | Thylakoid-bound ascorbate peroxidase APx8 | 44.8 | 17 | 0.75 | 0.0429 |  |
| 91 | A0A1D6FWI8 | 541786 | Uncharacterized protein | 30.6 | 4 | 0.74 | 0.0160 | Selenocompound metabolism |
| 92 | A0A1D6IAE3 | ZEAMMB73_Zm00001d021351 | Elongation factor 1-alpha | 42.1 | 15 | 0.73 | 0.0078 |  |
| 93 | B4FX25 | 100285211 | Acyl-CoA-binding protein | 48.3 | 4 | 0.73 | 0.0084 | Amino sugar and nucleotide sugar metabolism |
| 94 | B4FJK0 | 100216750 | 40S ribosomal protein S29 | 37.5 | 2 | 0.73 | 0.0079 |  |
| 95 | C0P401 |  | Cysteine synthase | 61.3 | 17 | 0.73 | 0.0173 |  |
| 96 | A0A1D6LGD3 | ZEAMMB73_Zm00001d035434 | NAD(P)H-dependent oxidoreductase | 33.2 | 8 | 0.73 | 0.0048 |  |
| 97 | A0A1D6NDN3 | ZEAMMB73_Zm00001d043620 | 60S ribosomal protein L6 | 53.0 | 12 | 0.73 | 0.0237 | Oxidative phosphorylation |
| 98 | B6TTP0 | 100502113 | Oxidoreductase | 20.9 | 6 | 0.73 | 0.0006 |  |
| 99 | A0A1D6KKU1 | 103643076 | Malic enzyme | 69.0 | 35 | 0.73 | 0.0008 |  |
| 100 | A0A1D6N0K3 | ZEAMMB73_Zm00001d042022 | Putative plastid-lipid-associated protein 12 chloroplastic | 5.2 | 2 | 0.73 | 0.0236 | Phenylpropanoid biosynthesis |
| 101 | A0A1D6PPE3 | 100274279 | La-related protein 6B | 1.9 | 1 | 0.71 | 0.0112 |  |
| 102 | B4G175 |  | USP family protein | 16.3 | 2 | 0.70 | 0.0387 |  |
| 103 | A0A1D6JKT7 | 100193669 | Glutaredoxin family protein | 8.7 | 2 | 0.68 | 0.0013 |  |
| 104 | B6SYW3 |  | Dirigent protein | 34.3 | 4 | 0.67 | 0.0119 |  |
| 105 | A0A1D6KLS9 | 100192754 | Protease Do-like 1 chloroplastic | 31.9 | 10 | 0.63 | 0.0010 | Porphyrin and chlorophyll metabolism |
| 106 | C0HIH1 |  | Abscisic acid stress ripening1 | 47.1 | 4 | 0.60 | 0.0325 |  |
| 107 | A0A1D6EB22 | ZEAMMB73_Zm00001d003712 | Probable UDP-arabinopyranose mutase 1 | 61.8 | 17 | 0.58 | 0.0380 |  |

^1^Protein ID, unique protein identifying number in the UniProt database; ^2^Gene name; name or ID number of the corresponding gene of the identified differentially abundant protein as searched against the maize sequence database Gramene ([http://ensemble.gramene.org/Zea mays](http://ensemble.gramene.org/Zea%20mays)); ^3^Description, annotated biological functions based on Gene Ontology (GO) analysis; ^4^ Coverage (%), sequence coverage is calculated as the number of amino acids in the peptide fragments observed divided by the protein amino acid length; ^5^ Peptides fragments, refer to the number of matched peptide fragments generated by trypsin digestion; ^6^ Ratio (SD_TD), is the ratio of intensities of up-regulated or down-regulated proteins between drought stressed sensitive line and drought stressed tolerant line; All the fold change figures below 1 represents that the proteins were down-regulated. All the figures above 1 means the proteins were up-regulated; ^7^ *p* value, statistical level (using Student`s *t*-test) below < 0.05, at which protein differential expression was accepted as significant; ^8^Pathways, metabolic KEGG pathways in which the identified protein was found to be significantly enriched; ^9^ uncharacterized protein, a protein without any functional annotations ascribed to it at the present.
