# Supplementary material for: Comparative Proteomic and Physiological Analyses of Two Divergent Maize Inbred Lines Provide More Insights into Drought-Stress Tolerance Mechanisms
Source: Int J Mol Sci. 2018 Oct 18;19(10):3225. doi: 10.3390/ijms19103225 (PMC6213998; doi:10.3390/ijms19103225)
Supplement: Supplementary file 1 [file ijms-19-03225-s001.zip › Supplementary Material/SUPPLEMENTARY TABLES/Supplementary Table 5 Enriched GO terms of the DAPs in YE8112 ( TC_TD).docx]

**Supplementary Table 5.** Enriched GO terms of the DAPs specific to YE8112 before and after drought treatment comparison (TC_TD)

| **No.** | **GO_ID** | **GO term** | **Category** | **Representation** | **P value** | **FDR** | **Rich factor** |  |
| --- | --- | --- | --- | --- | --- | --- | --- | --- |
| 1 | GO:0010196 | nonphotochemical quenching | P | + | 0.0003 | 0.018959 | 0.667 | |
| 2 | GO:1990066 | energy quenching | P | + | 0.0003 | 0.018959 | 0.667 | |
| 3 | GO:0010155 | regulation of proton transport | P | + | 0.0003 | 0.018959 | 0.667 | |
| 4 | GO:0009744 | response to sucrose | P | + | 0.0009 | 0.049937 | 0.400 | |
| 5 | GO:0034285 | response to disaccharide | P | + | 0.0009 | 0.049937 | 0.400 | |
| 6 | GO:0009644 | response to high light intensity | P | + | 0.0032 | 0.144518 | 0.222 | |
| 7 | GO:0009642 | response to light intensity | P | + | 0.0040 | 0.144518 | 0.200 | |
| 8 | GO:0009743 | response to carbohydrate | P | + | 0.0048 | 0.144518 | 0.182 | |
| 9 | GO:0043269 | regulation of ion transport | P | + | 0.0058 | 0.144518 | 0.167 | |
| 10 | GO:0019344 | cysteine biosynthetic process | P | + | 0.0058 | 0.144518 | 0.167 | |
| 11 | GO:1901700 | response to oxygen-containing compound | P | + | 0.0069 | 0.144518 | 0.051 | |
| 12 | GO:0044272 | sulfur compound biosynthetic process | P | + | 0.0076 | 0.144518 | 0.071 | |
| 13 | GO:0051049 | regulation of transport | P | + | 0.0079 | 0.144518 | 0.143 | |
| 14 | GO:0046133 | pyrimidine ribonucleoside catabolic process | P | + | 0.0098 | 0.144518 | 1.000 | |
| 15 | GO:0010167 | response to nitrate | P | + | 0.0098 | 0.144518 | 1.000 | |
| 16 | GO:0046135 | pyrimidine nucleoside catabolic process | P | + | 0.0098 | 0.144518 | 1.000 | |
| 17 | GO:0010106 | cellular response to iron ion starvation | P | + | 0.0098 | 0.144518 | 1.000 | |
| 18 | GO:0042759 | long-chain fatty acid biosynthetic process | P | + | 0.0098 | 0.144518 | 1.000 | |
| 19 | GO:1902609 | (R)-2-hydroxy-alpha-linolenic acid biosynthetic process | P | + | 0.0098 | 0.144518 | 1.000 | |
| 20 | GO:0006216 | cytidine catabolic process | P | + | 0.0098 | 0.144518 | 1.000 | |
| 21 | GO:0006636 | unsaturated fatty acid biosynthetic process | P | + | 0.0098 | 0.144518 | 1.000 | |
| 22 | GO:0033559 | unsaturated fatty acid metabolic process | P | + | 0.0098 | 0.144518 | 1.000 | |
| 23 | GO:0001561 | fatty acid alpha-oxidation | P | + | 0.0098 | 0.144518 | 1.000 | |
| 24 | GO:0046087 | cytidine metabolic process | P | + | 0.0098 | 0.144518 | 1.000 | |
| 25 | GO:0009972 | cytidine deamination | P | + | 0.0098 | 0.144518 | 1.000 | |
| 26 | GO:0032879 | regulation of localization | P | + | 0.0102 | 0.146692 | 0.125 | |
| 27 | GO:0006534 | cysteine metabolic process | P | + | 0.0115 | 0.146692 | 0.118 | |
| 28 | GO:0010033 | response to organic substance | P | + | 0.0135 | 0.166304 | 0.042 | |
| 29 | GO:0009070 | serine family amino acid biosynthetic process | P | + | 0.0174 | 0.166304 | 0.095 | |
| 30 | GO:0042454 | ribonucleoside catabolic process | P | + | 0.0195 | 0.166304 | 0.500 | |
| 31 | GO:0015706 | nitrate transport | P | + | 0.0195 | 0.166304 | 0.500 | |
| 32 | GO:0009164 | nucleoside catabolic process | P | + | 0.0195 | 0.166304 | 0.500 | |
| 33 | GO:1901658 | glycosyl compound catabolic process | P | + | 0.0195 | 0.166304 | 0.500 | |
| 34 | GO:0031667 | response to nutrient levels | P | + | 0.0195 | 0.166304 | 0.500 | |
| 35 | GO:0042594 | response to starvation | P | + | 0.0195 | 0.166304 | 0.500 | |
| 36 | GO:0009267 | cellular response to starvation | P | + | 0.0195 | 0.166304 | 0.500 | |
| 37 | GO:0031669 | cellular response to nutrient levels | P | + | 0.0195 | 0.166304 | 0.500 | |
| 38 | GO:0006556 | S-adenosylmethionine biosynthetic process | P | + | 0.0195 | 0.166304 | 0.500 | |
| 39 | GO:0000097 | sulfur amino acid biosynthetic process | P | + | 0.0207 | 0.174015 | 0.087 | |
| 40 | GO:0097305 | response to alcohol | P | + | 0.0224 | 0.18205 | 0.083 | |
| 41 | GO:0009737 | response to abscisic acid | P | + | 0.0224 | 0.18205 | 0.083 | |
| 42 | GO:0006812 | cation transport | P | + | 0.0226 | 0.18205 | 0.036 | |
| 43 | GO:0033993 | response to lipid | P | + | 0.0280 | 0.193772 | 0.074 | |
| 44 | GO:0006527 | arginine catabolic process | P | + | 0.0290 | 0.193772 | 0.333 | |
| 45 | GO:0071496 | cellular response to external stimulus | P | + | 0.0290 | 0.193772 | 0.333 | |
| 46 | GO:0006826 | iron ion transport | P | + | 0.0290 | 0.193772 | 0.333 | |
| 47 | GO:0001676 | long-chain fatty acid metabolic process | P | + | 0.0290 | 0.193772 | 0.333 | |
| 48 | GO:0031668 | cellular response to extracellular stimulus | P | + | 0.0290 | 0.193772 | 0.333 | |
| 49 | GO:0009991 | response to extracellular stimulus | P | + | 0.0290 | 0.193772 | 0.333 | |
| 50 | GO:0000165 | MAPK cascade | P | + | 0.0290 | 0.193772 | 0.333 | |
| 51 | GO:0072529 | pyrimidine-containing compound catabolic process | P | + | 0.0290 | 0.193772 | 0.333 | |
| 52 | GO:0000096 | sulfur amino acid metabolic process | P | + | 0.0300 | 0.197489 | 0.071 | |
| 53 | GO:0006790 | sulfur compound metabolic process | P | + | 0.0307 | 0.199642 | 0.033 | |
| 54 | GO:0009768 | photosynthesis, light harvesting in photosystem I | P | + | 0.0341 | 0.214196 | 0.067 | |
| 55 | GO:0008295 | spermidine biosynthetic process | P | + | 0.0385 | 0.218598 | 0.250 | |
| 56 | GO:0008216 | spermidine metabolic process | P | + | 0.0385 | 0.218598 | 0.250 | |
| 57 | GO:0000041 | transition metal ion transport | P | + | 0.0385 | 0.218598 | 0.250 | |
| 58 | GO:0009611 | response to wounding | P | + | 0.0385 | 0.218598 | 0.250 | |
| 59 | GO:0009627 | systemic acquired resistance | P | + | 0.0385 | 0.218598 | 0.250 | |
| 60 | GO:0009069 | serine family amino acid metabolic process | P | + | 0.0406 | 0.218598 | 0.061 | |
| 61 | GO:0006818 | hydrogen transport | P | + | 0.0409 | 0.218598 | 0.038 | |
| 62 | GO:0015992 | proton transport | P | + | 0.0409 | 0.218598 | 0.038 | |
| 63 | GO:0046395 | carboxylic acid catabolic process | P | + | 0.0452 | 0.218598 | 0.057 | |
| 64 | GO:0016054 | organic acid catabolic process | P | + | 0.0452 | 0.218598 | 0.057 | |
| 65 | GO:0006811 | ion transport | P | + | 0.0460 | 0.218598 | 0.029 | |
| 66 | GO:1905950 | monosaccharide transmembrane transport | P | + | 0.0480 | 0.218598 | 0.200 | |
| 67 | GO:0009445 | putrescine metabolic process | P | + | 0.0480 | 0.218598 | 0.200 | |
| 68 | GO:0008645 | hexose transport | P | + | 0.0480 | 0.218598 | 0.200 | |
| 69 | GO:0046323 | glucose import | P | + | 0.0480 | 0.218598 | 0.200 | |
| 70 | GO:1904659 | glucose transmembrane transport | P | + | 0.0480 | 0.218598 | 0.200 | |
| 71 | GO:0015698 | inorganic anion transport | P | + | 0.0480 | 0.218598 | 0.200 | |
| 72 | GO:0015749 | monosaccharide transport | P | + | 0.0480 | 0.218598 | 0.200 | |
| 73 | GO:0009631 | cold acclimation | P | + | 0.0480 | 0.218598 | 0.200 | |
| 74 | GO:0009446 | putrescine biosynthetic process | P | + | 0.0480 | 0.218598 | 0.200 | |
| 75 | GO:0035428 | hexose transmembrane transport | P | + | 0.0480 | 0.218598 | 0.200 | |
| 76 | GO:0015758 | glucose transport | P | + | 0.0480 | 0.218598 | 0.200 | |
| 77 | GO:0009503 | thylakoid light-harvesting complex | C | + | 0.0001 | 0.010174 | 1.000 | |
| 78 | GO:0030076 | light-harvesting complex | C | + | 0.0001 | 0.010174 | 1.000 | |
| 79 | GO:0009783 | photosystem II antenna complex | C | + | 0.0001 | 0.010174 | 1.000 | |
| 80 | GO:0098807 | chloroplast thylakoid membrane protein complex | C | + | 0.0001 | 0.010174 | 1.000 | |
| 81 | GO:0009517 | PSII associated light-harvesting complex II | C | + | 0.0001 | 0.010174 | 1.000 | |
| 82 | GO:0012511 | monolayer-surrounded lipid storage body | C | + | 0.0098 | 0.144518 | 1.000 | |
| 83 | GO:0005811 | lipid droplet | C | + | 0.0098 | 0.144518 | 1.000 | |
| 84 | GO:0044431 | Golgi apparatus part | C | + | 0.0115 | 0.146692 | 0.118 | |
| 85 | GO:0031410 | cytoplasmic vesicle | C | + | 0.0115 | 0.146692 | 0.118 | |
| 86 | GO:0097708 | intracellular vesicle | C | + | 0.0115 | 0.146692 | 0.118 | |
| 87 | GO:0031982 | vesicle | C | + | 0.0115 | 0.146692 | 0.118 | |
| 88 | GO:0030127 | COPII vesicle coat | C | + | 0.0195 | 0.166304 | 0.500 | |
| 89 | GO:0012507 | ER to Golgi transport vesicle membrane | C | + | 0.0195 | 0.166304 | 0.500 | |
| 90 | GO:0030134 | COPII-coated ER to Golgi transport vesicle | C | + | 0.0195 | 0.166304 | 0.500 | |
| 91 | GO:0005802 | trans-Golgi network | C | + | 0.0290 | 0.193772 | 0.333 | |
| 92 | GO:0005768 | endosome | C | + | 0.0480 | 0.218598 | 0.200 | |
| 93 | GO:0012505 | endomembrane system, hydrolase activity, acting on carbon nitrogen bonds | C | + | 0.0491 | 0.222111 | 0.028 | |
| 94 | GO:0016814 | amidines | F | + | 0.0058 | 0.144518 | 0.167 | |
| 95 | GO:0004126 | cytidine deaminase activity | F | + | 0.0098 | 0.144518 | 1.000 | |
| 96 | GO:0010333 | terpene synthase activity | F | + | 0.0098 | 0.144518 | 1.000 | |
| 97 | GO:0003937 | IMP cyclohydrolase activity | F | + | 0.0098 | 0.144518 | 1.000 | |
| 98 | GO:0004643 | phosphoribosylaminoimidazolecarboxamide formyltransferase activity | F | + | 0.0098 | 0.144518 | 1.000 | |
| 99 | GO:0051213 | dioxygenase activity | F | + | 0.0143 | 0.166304 | 0.105 | |
| 100 | GO:0005057 | signal transducer activity, downstream of receptor | F | + | 0.0195 | 0.166304 | 0.500 | |
| 101 | GO:0004707 | MAP kinase activity, oxidoreductase activity, acting on paired donors | F | + | 0.0195 | 0.166304 | 0.500 | |
| 102 | GO:0016706 | of oxygen into both donors | F | + | 0.0195 | 0.166304 | 0.500 | |
| 103 | GO:0008792 | arginine decarboxylase activity | F | + | 0.0195 | 0.166304 | 0.500 | |
| 104 | GO:0004478 | methionine adenosyltransferase activity | F | + | 0.0195 | 0.166304 | 0.500 | |
| 105 | GO:0004702 | signal transducer, downstream of receptor, with serine/threonine kinase activity | F | + | 0.0195 | 0.166304 | 0.500 | |
| 106 | GO:0004749 | ribose phosphate diphosphokinase activity | F | + | 0.0290 | 0.193772 | 0.333 | |
| 107 | GO:0016778 | diphosphotransferase activity | F | + | 0.0290 | 0.193772 | 0.333 | |
| 108 | GO:0036459 | thiol-dependent ubiquitinyl hydrolase activity | F | + | 0.0290 | 0.193772 | 0.333 | |
| 109 | GO:0101005 | ubiquitinyl hydrolase activity | F | + | 0.0290 | 0.193772 | 0.333 | |
| 110 | GO:0046906 | tetrapyrrole binding | F | + | 0.0339 | 0.214196 | 0.031 | |
| 111 | GO:0031409 | pigment binding | F | + | 0.0341 | 0.214196 | 0.067 | |
| 112 | GO:0016838 | carbon-oxygen lyase activity, acting on phosphates | F | + | 0.0385 | 0.218598 | 0.250 | |
| 113 | GO:0016810 | hydrolase activity, acting on carbon-nitrogen (but not peptide) bonds | F | + | 0.0406 | 0.218598 | 0.061 | |
| 114 | GO:0005355 | glucose transmembrane transporter activity | F | + | 0.0480 | 0.218598 | 0.200 | |
| 115 | GO:0008889 | glycerophosphodiester phosphodiesterase activity | F | + | 0.0480 | 0.218598 | 0.200 | |
| 116 | GO:0019783 | ubiquitin-like protein-specific protease activity | F | + | 0.0480 | 0.218598 | 0.200 | |
| 117 | GO:0005402 | cation:sugar symporter activity | F | + | 0.0480 | 0.218598 | 0.200 | |
| 118 | GO:0015295 | solute:proton symporter activity | F | + | 0.0480 | 0.218598 | 0.200 | |
| 119 | GO:0015149 | hexose transmembrane transporter activity | F | + | 0.0480 | 0.218598 | 0.200 | |
| 120 | GO:0015145 | monosaccharide transmembrane transporter activity | F | + | 0.0480 | 0.218598 | 0.200 | |
| 121 | GO:0005351 | sugar:proton symporter activity | F | + | 0.0480 | 0.218598 | 0.200 | |

**Note**: GO ID: GO term ID; Ontology: GO function; + , overrepresented; P, biological process; C, cell component; F, molecular function; P<0.05 significant level; FDR, false discovery rate; Rich factor,
