# Supplementary material for: Comparative Proteomic and Physiological Analyses of Two Divergent Maize Inbred Lines Provide More Insights into Drought-Stress Tolerance Mechanisms
Source: Int J Mol Sci. 2018 Oct 18;19(10):3225. doi: 10.3390/ijms19103225 (PMC6213998; doi:10.3390/ijms19103225)
Supplement: Supplementary file 1 [file ijms-19-03225-s001.zip › Supplementary Material/SUPPLEMENTARY TABLES/Supplementary Table 6 Enriched GO terms in SD_TD.docx]

**Supplementary Table 6.** Enriched GO terms in the SD_TD comparison group.

| **No.** | **GO_ID** | **Term** | **Category** | **Representation** | **P value** | **FDR** | **Rich factor** |
| --- | --- | --- | --- | --- | --- | --- | --- |
| 1 | GO:0034728 | nucleosome organization | P | + | 0.0002 | 0.0258 | 0.3333 |
| 2 | GO:0006334 | nucleosome assembly | P | + | 0.0002 | 0.0258 | 0.3333 |
| 3 | GO:0071824 | protein-DNA complex subunit organization | P | + | 0.0002 | 0.0258 | 0.2903 |
| 4 | GO:0065004 | protein-DNA complex assembly | P | + | 0.0002 | 0.0258 | 0.2903 |
| 5 | GO:0006333 | chromatin assembly or disassembly | P | + | 0.0002 | 0.0258 | 0.3200 |
| 6 | GO:0031497 | chromatin assembly | P | + | 0.0002 | 0.0258 | 0.3200 |
| 7 | GO:0006323 | DNA packaging | P | + | 0.0003 | 0.0324 | 0.3077 |
| 8 | GO:0006325 | chromatin organization | P | + | 0.0009 | 0.0875 | 0.2432 |
| 9 | GO:0071103 | DNA conformation change | P | + | 0.0014 | 0.1308 | 0.2500 |
| 10 | GO:0044085 | cellular component biogenesis | P | + | 0.0015 | 0.1308 | 0.1292 |
| 11 | GO:0051276 | chromosome organization | P | + | 0.0043 | 0.2856 | 0.1957 |
| 12 | GO:0000463 | maturation of LSU-rRNA from tricistronic rRNA transcript (SSU-rRNA, 5.8S rRNA, LSU-rRNA) | P | + | 0.0050 | 0.2856 | 1.0000 |
| 13 | GO:0007623 | circadian rhythm | P | + | 0.0050 | 0.2856 | 1.0000 |
| 14 | GO:0048511 | rhythmic process | P | + | 0.0050 | 0.2856 | 1.0000 |
| 15 | GO:0090502 | RNA phosphodiester bond hydrolysis, endonucleolytic | P | + | 0.0100 | 0.4522 | 0.4286 |
| 16 | GO:0065003 | macromolecular complex assembly | P | + | 0.0100 | 0.4522 | 0.1277 |
| 17 | GO:0034622 | cellular macromolecular complex assembly | P | + | 0.0112 | 0.4907 | 0.1288 |
| 18 | GO:0048638 | regulation of developmental growth | P | + | 0.0144 | 0.4975 | 0.6667 |
| 19 | GO:0033356 | UDP-L-arabinose metabolic process | P | + | 0.0144 | 0.4975 | 0.6667 |
| 20 | GO:0010075 | regulation of meristem growth | P | + | 0.0144 | 0.4975 | 0.6667 |
| 21 | GO:0090501 | RNA phosphodiester bond hydrolysis | P | + | 0.0152 | 0.4975 | 0.3750 |
| 22 | GO:0022607 | cellular component assembly | P | + | 0.0153 | 0.4975 | 0.1203 |
| 23 | GO:0009404 | toxin metabolic process | P | + | 0.0182 | 0.5815 | 0.2667 |
| 24 | GO:0006790 | sulfur compound metabolic process | P | + | 0.0263 | 0.6671 | 0.1220 |
| 25 | GO:0042493 | response to drug | P | + | 0.0274 | 0.6671 | 0.5000 |
| 26 | GO:0035266 | meristem growth | P | + | 0.0274 | 0.6671 | 0.5000 |
| 27 | GO:0015893 | drug transport | P | + | 0.0274 | 0.6671 | 0.5000 |
| 28 | GO:0009832 | plant-type cell wall biogenesis | P | + | 0.0274 | 0.6671 | 0.5000 |
| 29 | GO:0006855 | drug transmembrane transport | P | + | 0.0274 | 0.6671 | 0.5000 |
| 30 | GO:0010073 | meristem maintenance | P | + | 0.0274 | 0.6671 | 0.5000 |
| 31 | GO:0048509 | regulation of meristem development | P | + | 0.0274 | 0.6671 | 0.5000 |
| 32 | GO:0043933 | macromolecular complex subunit organization | P | + | 0.0279 | 0.6671 | 0.1146 |
| 33 | GO:0090305 | nucleic acid phosphodiester bond hydrolysis | P | + | 0.0293 | 0.6905 | 0.3000 |
| 34 | GO:0071840 | cellular component organization or biogenesis | P | + | 0.0372 | 0.7261 | 0.0959 |
| 35 | GO:0040008 | regulation of growth | P | + | 0.0436 | 0.7261 | 0.4000 |
| 36 | GO:0009631 | cold acclimation | P | + | 0.0436 | 0.7261 | 0.4000 |
| 37 | GO:0048589 | developmental growth | P | + | 0.0483 | 0.7261 | 0.2500 |
| 38 | GO:0046982 | protein heterodimerization activity | F | + | 0.0000 | 0.0066 | 0.3600 |
| 39 | GO:0046983 | protein dimerization activity | F | + | 0.0021 | 0.1823 | 0.1897 |
| 40 | GO:0004473 | malate dehydrogenase (decarboxylating) (NADP) activity | F | + | 0.0050 | 0.2856 | 1.0000 |
| 41 | GO:0004521 | endoribonuclease activity | F | + | 0.0060 | 0.3086 | 0.5000 |
| 42 | GO:0003682 | chromatin binding | F | + | 0.0100 | 0.4522 | 0.4286 |
| 43 | GO:0004540 | ribonuclease activity | F | + | 0.0100 | 0.4522 | 0.4286 |
| 44 | GO:0003677 | DNA binding | F | + | 0.0143 | 0.4975 | 0.1346 |
| 45 | GO:0016894 | endonuclease activity, active with either ribo- or deoxyribonucleic acids and producing 3'-phosphomonoesters | F | + | 0.0144 | 0.4975 | 0.6667 |
| 46 | GO:0052691 | UDP-arabinopyranose mutase activity | F | + | 0.0144 | 0.4975 | 0.6667 |
| 47 | GO:0033897 | ribonuclease T2 activity | F | + | 0.0144 | 0.4975 | 0.6667 |
| 48 | GO:0033743 | peptide-methionine (R)-S-oxide reductase activity | F | + | 0.0144 | 0.4975 | 0.6667 |
| 49 | GO:0016892 | endoribonuclease activity, producing 3'-phosphomonoesters | F | + | 0.0144 | 0.4975 | 0.6667 |
| 50 | GO:0004519 | endonuclease activity | F | + | 0.0152 | 0.4975 | 0.3750 |
| 51 | GO:0016667 | oxidoreductase activity, acting on a sulfur group of donors | F | + | 0.0197 | 0.6172 | 0.1552 |
| 52 | GO:0004518 | nuclease activity | F | + | 0.0216 | 0.6536 | 0.3333 |
| 53 | GO:0008948 | oxaloacetate decarboxylase activity | F | + | 0.0274 | 0.6671 | 0.5000 |
| 54 | GO:0015238 | drug transmembrane transporter activity | F | + | 0.0274 | 0.6671 | 0.5000 |
| 55 | GO:0090484 | drug transporter activity | F | + | 0.0274 | 0.6671 | 0.5000 |
| 56 | GO:0005515 | protein binding | F | + | 0.0353 | 0.7261 | 0.1050 |
| 57 | GO:0016671 | oxidoreductase activity, acting on a sulfur group of donors, disulfide as acceptor | F | + | 0.0387 | 0.7261 | 0.1852 |
| 58 | GO:0016725 | oxidoreductase activity, acting on CH or CH2 groups | F | + | 0.0436 | 0.7261 | 0.4000 |
| 59 | GO:0004124 | cysteine synthase activity | F | + | 0.0436 | 0.7261 | 0.4000 |
| 60 | GO:0000785 | chromatin | C | + | 0.0000 | 0.0009 | 0.3529 |
| 61 | GO:0044427 | chromosomal part | C | + | 0.0000 | 0.0009 | 0.3429 |
| 62 | GO:0000786 | nucleosome | C | + | 0.0000 | 0.0009 | 0.3667 |
| 63 | GO:0044815 | DNA packaging complex | C | + | 0.0000 | 0.0009 | 0.3667 |
| 64 | GO:0032993 | protein-DNA complex | C | + | 0.0000 | 0.0009 | 0.3667 |
| 65 | GO:0005694 | chromosome | C | + | 0.0000 | 0.0066 | 0.2791 |
| 66 | GO:0000788 | nuclear nucleosome | C | + | 0.0032 | 0.2571 | 0.6000 |
| 67 | GO:0000790 | nuclear chromatin | C | + | 0.0037 | 0.2849 | 0.4000 |
| 68 | GO:0098687 | chromosomal region | C | + | 0.0050 | 0.2856 | 1.0000 |
| 69 | GO:0000775 | chromosome, centromeric region | C | + | 0.0050 | 0.2856 | 1.0000 |
| 70 | GO:0043228 | non-membrane-bounded organelle | C | + | 0.0222 | 0.6536 | 0.1009 |
| 71 | GO:0043232 | intracellular non-membrane-bounded organelle | C | + | 0.0222 | 0.6536 | 0.1009 |
| 72 | GO:0044454 | nuclear chromosome part | C | + | 0.0055 | 0.2917 | 0.3636 |
| 73 | GO:0000228 | nuclear chromosome | C | + | 0.0055 | 0.2917 | 0.3636 |

**Note**: GO ID: GO term ID; Ontology: GO function; + , overrepresented; P, biological process; C, cell component; F, molecular function; P<0.05 significant level; FDR, false discovery rate; Rich factor,
