# Supplementary material for: Comparative Proteomic and Physiological Analyses of Two Divergent Maize Inbred Lines Provide More Insights into Drought-Stress Tolerance Mechanisms
Source: Int J Mol Sci. 2018 Oct 18;19(10):3225. doi: 10.3390/ijms19103225 (PMC6213998; doi:10.3390/ijms19103225)
Supplement: Supplementary file 1 [file ijms-19-03225-s001.zip › Supplementary Material/SUPPLEMENTARY TABLES/Supplementary Table 7 Enriched GO terms of theDAPs in MO17 (SC_SD).docx]

**Supplementary Table 7.** Enriched GO terms of the DAPs specific to MO17 before and after drought treatment comparison (SD_SC)

| **No.** | **GO_ID** | **GO term** | **Category** | **Representation** | **P value** | **FDR** | **Rich factor** | |
| --- | --- | --- | --- | --- | --- | --- | --- | --- |
| 1 | GO:0007059 | chromosome segregation | P | + | 0.009708 | 0.471316 | 0.5 |  |
| 2 | GO:0006265 | DNA topological change | P | + | 0.009708 | 0.471316 | 0.5 |  |
| 3 | GO:0006338 | chromatin remodeling | P | + | 0.009708 | 0.471316 | 0.5 |  |
| 4 | GO:0051276 | chromosome organization | P | + | 0.010964 | 0.471316 | 0.130435 |  |
| 5 | GO:0009081 | branched-chain amino acid metabolic process | P | + | 0.014735 | 0.471316 | 0.230769 |  |
| 6 | GO:0016192 | vesicle-mediated transport | P | + | 0.015338 | 0.471316 | 0.138889 |  |
| 7 | GO:0010228 | vegetative to reproductive phase transition of meristem | P | + | 0.015741 | 0.471316 | 0.4 |  |
| 8 | GO:0006551 | leucine metabolic process | P | + | 0.015741 | 0.471316 | 0.4 |  |
| 9 | GO:0006325 | chromatin organization | P | + | 0.017156 | 0.471316 | 0.135135 |  |
| 10 | GO:1901264 | carbohydrate derivative transport | P | + | 0.022974 | 0.471316 | 0.333333 |  |
| 11 | GO:0046907 | intracellular transport | P | + | 0.031753 | 0.471316 | 0.088889 |  |
| 12 | GO:0006886 | intracellular protein transport | P | + | 0.036699 | 0.471316 | 0.092105 |  |
| 13 | GO:0034219 | carbohydrate transmembrane transport | P | + | 0.040608 | 0.471316 | 0.25 |  |
| 14 | GO:0006260 | DNA replication | P | + | 0.040608 | 0.471316 | 0.25 |  |
| 15 | GO:0061504 | cyclic threonylcarbamoyladenosine biosynthetic process | P | + | 0.04148 | 0.471316 | 1 |  |
| 16 | GO:0048235 | pollen sperm cell differentiation | P | + | 0.04148 | 0.471316 | 1 |  |
| 17 | GO:0048232 | male gamete generation | P | + | 0.04148 | 0.471316 | 1 |  |
| 18 | GO:0022412 | cellular process involved in reproduction in multicellular organism | P | + | 0.04148 | 0.471316 | 1 |  |
| 19 | GO:0018212 | peptidyl-tyrosine modification | P | + | 0.04148 | 0.471316 | 1 |  |
| 20 | GO:0036065 | fucosylation | P | + | 0.04148 | 0.471316 | 1 |  |
| 21 | GO:0071569 | protein ufmylation | P | + | 0.04148 | 0.471316 | 1 |  |
| 22 | GO:0048571 | long-day photoperiodism | P | + | 0.04148 | 0.471316 | 1 |  |
| 23 | GO:0055046 | microgametogenesis | P | + | 0.04148 | 0.471316 | 1 |  |
| 24 | GO:0046836 | glycolipid transport | P | + | 0.04148 | 0.471316 | 1 |  |
| 25 | GO:0006268 | DNA unwinding involved in DNA replication | P | + | 0.04148 | 0.471316 | 1 |  |
| 26 | GO:0010051 | xylem and phloem pattern formation | P | + | 0.04148 | 0.471316 | 1 |  |
| 27 | GO:0009804 | coumarin metabolic process | P | + | 0.04148 | 0.471316 | 1 |  |
| 28 | GO:0048574 | long-day photoperiodism, flowering | P | + | 0.04148 | 0.471316 | 1 |  |
| 29 | GO:0071266 | 'de novo' L-methionine biosynthetic process | P | + | 0.04148 | 0.471316 | 1 |  |
| 30 | GO:0009805 | coumarin biosynthetic process | P | + | 0.04148 | 0.471316 | 1 |  |
| 31 | GO:0007276 | gamete generation | P | + | 0.04148 | 0.471316 | 1 |  |
| 32 | GO:0018108 | peptidyl-tyrosine phosphorylation | P | + | 0.04148 | 0.471316 | 1 |  |
| 33 | GO:0071103 | DNA conformation change | P | + | 0.041492 | 0.471316 | 0.125 |  |
| 34 | GO:0006414 | translational elongation | P | + | 0.045744 | 0.504906 | 0.121212 |  |
| 35 | GO:0051649 | establishment of localization in cell | P | + | 0.046489 | 0.50834 | 0.082474 |  |
| 36 | GO:0033036 | macromolecule localization | P | + | 0.047575 | 0.510903 | 0.078261 |  |
| 37 | GO:0071702 | organic substance transport | P | + | 0.030711 | 0.471316 | 0.078014 |  |
| 38 | GO:0044427 | chromosomal part | C | + | 0.000466 | 0.471316 | 0.2 |  |
| 39 | GO:0005694 | chromosome | C | + | 0.001689 | 0.471316 | 0.162791 |  |
| 40 | GO:0000785 | chromatin | C | + | 0.002371 | 0.471316 | 0.176471 |  |
| 41 | GO:0000786 | nucleosome | C | + | 0.007078 | 0.471316 | 0.166667 |  |
| 42 | GO:0044815 | DNA packaging complex | C | + | 0.007078 | 0.471316 | 0.166667 |  |
| 43 | GO:0032993 | protein-DNA complex | C | + | 0.007078 | 0.471316 | 0.166667 |  |
| 44 | GO:0070013 | intracellular organelle lumen | C | + | 0.010853 | 0.471316 | 0.090909 |  |
| 45 | GO:0031974 | membrane-enclosed lumen | C | + | 0.010853 | 0.471316 | 0.090909 |  |
| 46 | GO:0043233 | organelle lumen | C | + | 0.010853 | 0.471316 | 0.090909 |  |
| 47 | GO:0005759 | mitochondrial matrix | C | + | 0.008844 | 0.471316 | 0.136364 |  |
| 48 | GO:0000228 | nuclear chromosome | C | + | 0.009036 | 0.471316 | 0.272727 |  |
| 49 | GO:0044454 | nuclear chromosome part | C | + | 0.009036 | 0.471316 | 0.272727 |  |
| 50 | GO:0032991 | macromolecular complex | C | + | 0.018946 | 0.471316 | 0.057971 |  |
| 51 | GO:0030117 | membrane coat | C | + | 0.022061 | 0.471316 | 0.2 |  |
| 52 | GO:0048475 | coated membrane | C | + | 0.022061 | 0.471316 | 0.2 |  |
| 53 | GO:0030118 | clathrin coat | C | + | 0.022974 | 0.471316 | 0.333333 |  |
| 54 | GO:0031969 | chloroplast membrane | C | + | 0.026343 | 0.471316 | 0.1875 |  |
| 55 | GO:0031410 | cytoplasmic vesicle | C | + | 0.031036 | 0.471316 | 0.176471 |  |
| 56 | GO:0044431 | Golgi apparatus part | C | + | 0.031036 | 0.471316 | 0.176471 |  |
| 57 | GO:0031982 | vesicle | C | + | 0.031036 | 0.471316 | 0.176471 |  |
| 58 | GO:0097708 | intracellular vesicle | C | + | 0.031036 | 0.471316 | 0.176471 |  |
| 59 | GO:0005762 | mitochondrial large ribosomal subunit | C | + | 0.031296 | 0.471316 | 0.285714 |  |
| 60 | GO:0000315 | organellar large ribosomal subunit | C | + | 0.031296 | 0.471316 | 0.285714 |  |
| 61 | GO:0009706 | chloroplast inner membrane | C | + | 0.031296 | 0.471316 | 0.285714 |  |
| 62 | GO:0009528 | plastid inner membrane | C | + | 0.040608 | 0.471316 | 0.25 |  |
| 63 | GO:0097346 | INO80-type complex | C | + | 0.04148 | 0.471316 | 1 |  |
| 64 | GO:0005657 | replication fork | C | + | 0.04148 | 0.471316 | 1 |  |
| 65 | GO:0031298 | replication fork protection complex | C | + | 0.04148 | 0.471316 | 1 |  |
| 66 | GO:0009330 | DNA topoisomerase complex (ATP-hydrolyzing) | C | + | 0.04148 | 0.471316 | 1 |  |
| 67 | GO:0030008 | TRAPP complex | C | + | 0.04148 | 0.471316 | 1 |  |
| 68 | GO:0043596 | nuclear replication fork | C | + | 0.04148 | 0.471316 | 1 |  |
| 69 | GO:1902562 | H4 histone acetyltransferase complex | C | + | 0.04148 | 0.471316 | 1 |  |
| 70 | GO:0035267 | NuA4 histone acetyltransferase complex | C | + | 0.04148 | 0.471316 | 1 |  |
| 71 | GO:0033202 | DNA helicase complex | C | + | 0.04148 | 0.471316 | 1 |  |
| 72 | GO:0043189 | H4/H2A histone acetyltransferase complex | C | + | 0.04148 | 0.471316 | 1 |  |
| 73 | GO:0031011 | Ino80 complex | C | + | 0.04148 | 0.471316 | 1 |  |
| 74 | GO:0070603 | SWI/SNF superfamily-type complex | C | + | 0.04148 | 0.471316 | 1 |  |
| 75 | GO:0005886 | plasma membrane | C | + | 0.042631 | 0.479603 | 0.071856 |  |
| 76 | GO:0043228 | non-membrane-bounded organelle | C | + | 0.048034 | 0.510903 | 0.061162 |  |
| 77 | GO:0043232 | intracellular non-membrane-bounded organelle | C | + | 0.048034 | 0.510903 | 0.061162 |  |
| 78 | GO:0003676 | nucleic acid binding | F | + | 0.007377 | 0.471316 | 0.066194 |  |
| 79 | GO:0008135 | translation factor activity, RNA binding | F | + | 0.004065 | 0.471316 | 0.126984 |  |
| 80 | GO:0003924 | GTPase activity | F | + | 0.007035 | 0.471316 | 0.142857 |  |
| 81 | GO:0003918 | DNA topoisomerase type II (ATP-hydrolyzing) activity | F | + | 0.009708 | 0.471316 | 0.5 |  |
| 82 | GO:0061505 | DNA topoisomerase II activity | F | + | 0.009708 | 0.471316 | 0.5 |  |
| 83 | GO:0030976 | thiamine pyrophosphate binding | F | + | 0.015741 | 0.471316 | 0.4 |  |
| 84 | GO:0005048 | signal sequence binding | F | + | 0.015741 | 0.471316 | 0.4 |  |
| 85 | GO:0008171 | O-methyltransferase activity | F | + | 0.018193 | 0.471316 | 0.214286 |  |
| 86 | GO:0003746 | translation elongation factor activity | F | + | 0.02088 | 0.471316 | 0.153846 |  |
| 87 | GO:0003723 | RNA binding | F | + | 0.027168 | 0.471316 | 0.068273 |  |
| 88 | GO:0008094 | DNA-dependent ATPase activity | F | + | 0.031296 | 0.471316 | 0.285714 |  |
| 89 | GO:0005515 | protein binding | F | + | 0.036105 | 0.471316 | 0.068493 |  |
| 90 | GO:0140097 | catalytic activity, acting on DNA | F | + | 0.040608 | 0.471316 | 0.25 |  |
| 91 | GO:0046556 | alpha-L-arabinofuranosidase activity | F | + | 0.040608 | 0.471316 | 0.25 |  |
| 92 | GO:0047334 | diphosphate-fructose-6-phosphate 1-phosphotransferase activity | F | + | 0.04148 | 0.471316 | 1 |  |
| 93 | GO:0017089 | glycolipid transporter activity | F | + | 0.04148 | 0.471316 | 1 |  |
| 94 | GO:0061503 | tRNA threonylcarbamoyladenosine dehydratase | F | + | 0.04148 | 0.471316 | 1 |  |
| 95 | GO:0050342 | tocopherol O-methyltransferase activity | F | + | 0.04148 | 0.471316 | 1 |  |
| 96 | GO:0008107 | galactoside 2-alpha-L-fucosyltransferase activity | F | + | 0.04148 | 0.471316 | 1 |  |
| 97 | GO:0042409 | caffeoyl-CoA O-methyltransferase activity | F | + | 0.04148 | 0.471316 | 1 |  |
| 98 | GO:0004852 | uroporphyrinogen-III synthase activity | F | + | 0.04148 | 0.471316 | 1 |  |
| 99 | GO:0051861 | glycolipid binding | F | + | 0.04148 | 0.471316 | 1 |  |
| 100 | GO:0030942 | endoplasmic reticulum signal peptide binding | F | + | 0.04148 | 0.471316 | 1 |  |
| 101 | GO:0008417 | fucosyltransferase activity | F | + | 0.04148 | 0.471316 | 1 |  |
| 102 | GO:0003917 | DNA topoisomerase type I activity | F | + | 0.04148 | 0.471316 | 1 |  |
| 103 | GO:0017137 | Rab GTPase binding | F | + | 0.04148 | 0.471316 | 1 |  |
| 104 | GO:0017112 | Rab guanyl-nucleotide exchange factor activity | F | + | 0.04148 | 0.471316 | 1 |  |
| 105 | GO:0051741 | 2-methyl-6-phytyl-1,4-benzoquinone methyltransferase activity | F | + | 0.04148 | 0.471316 | 1 |  |
| 106 | GO:0004121 | cystathionine beta-lyase activity | F | + | 0.04148 | 0.471316 | 1 |  |
| 107 | GO:0031127 | alpha-(1,2)-fucosyltransferase activity | F | + | 0.04148 | 0.471316 | 1 |  |
| 108 | GO:0071566 | UFM1 activating enzyme activity | F | + | 0.04148 | 0.471316 | 1 |  |
| 109 | GO:0008757 | S-adenosylmethionine-dependent methyltransferase activity | F | + | 0.045744 | 0.504906 | 0.121212 |  |
| 110 | GO:0003916 | DNA topoisomerase activity | F | + | 0.009708 | 0.471316 | 0.5 |  |

**Note**: GO ID: GO term ID; Ontology: GO function; + , + represented; P, biological process; C, cell component; F, molecular function; P<0.05 significant level; FDR, false discovery rate; Rich factor,
