# Supplementary material for: Comparative Proteomic and Physiological Analyses of Two Divergent Maize Inbred Lines Provide More Insights into Drought-Stress Tolerance Mechanisms
Source: Int J Mol Sci. 2018 Oct 18;19(10):3225. doi: 10.3390/ijms19103225 (PMC6213998; doi:10.3390/ijms19103225)
Supplement: Supplementary file 1 [file ijms-19-03225-s001.zip › Supplementary Material/SUPPLEMENTARY TABLES/Supplementary Table 8 Summary of genes and primers used for qRT-PCR.docx]

**Supplementary Table 8**. Summary of genes and gene-specific primers used for qRT-PCR

| **No.** | **Gene name** | **Forward primer 5’-3'）** | **Reverse primer 5’-3'）** | **F Temp (˚C)** | **R Temp (˚C)** | **GC% (F)** | **GC% (R)** | **PCR Product** |
| --- | --- | --- | --- | --- | --- | --- | --- | --- |
| 1 | Lhcb5-1 | GCTGTGTGTGAGGGTCTGAA | CCAACTCAAACGCACCCTTA | 59.89 | 58.4 | 55 | 50 | 180 |
| 2 | 100193714 | AGGTAGCCCAATCGAAACCA | TACTTCTGCCCTCACTCCG | 59.01 | 59.1 | 50 | 55 | 191 |
| 3 | ZEAMMB73_Zm00001d012677 | GAATTGACATCCCCACACCG | TGCAAAGTCACAGTGTCACG | 58.91 | 58.99 | 55 | 50 | 194 |
| 4 | 100286059 | CCAGCACGAGACACAATCAC | CACGGTGTTCCCTTTTCTCG | 59.21 | 59.13 | 55 | 55 | 215 |
| 5 | 100383306 | TATCAGCACGTTTTCAGCGG | GTATCACCTTGGGCATGCTG | 58.92 | 58.97 | 50 | 55 | 210 |
| 6 | ZEAMMB73_Zm00001d039613 | AGGAAGACGTCGATGATGCT | CTGCCTTTCTGTGTCCTTGG | 58.89 | 58.76 | 50 | 55 | 190 |
| 7 | ZEAMMB73_Zm00001d021334 | GAGGTGCCCACTTTCGTCTT | TATCGAAGCGTTGCTTGTCG | 60.25 | 59.01 | 55 | 50 | 188 |
| 8 | 100191245 | GGATTGGATGCTTCGGATCG | ATAAGCTGATCGACCGAGGG | 58.85 | 59.04 | 55 | 55 | 189 |
| 9 | 101027254 | GGTCTTTTCTTCTGCCAGCC | AGCACCATGAGAACTCCCAA | 59.12 | 58.93 | 55 | 50 | 242 |
| 10 | TPS7 | CGCAGCTCACCTTGTTACTC | AAGCTCTAGTATCACGGGGC | 58.93 | 58.96 | 55 | 55 | 244 |
| 11 | 100383595 | AGAAGAAGAAGCGGAGGGAC | TTAGTGTCCTTCCCTGCTGG | 59.1 | 59.02 | 55 | 55 | 220 |
| 12 | 542304 | GCACTCTACGAGAAGCACGA | GGGAGGATCAATCAGCCGAA | 59.83 | 59.53 | 55 | 55 | 203 |
| 13 | 100282063 | TCAAAGGTTCAGTGGGTCCA | CGGACAGAAAGCATGGATGG | 58.78 | 58.98 | 50 | 55 | 178 |
| 14 | 100282951 | AAGATGTCCTTCGAGCCACA | ACTTCCCTCGGCTTCTCTTC | 59.02 | 59.1 | 50 | 55 | 248 |
| 15 | 100272744 | CCTGATGTTGGAGGTGTTGC | AATGGTACCTGCTGTGTCGA | 59.12 | 59.03 | 55 | 50 | 183 |
| 16 | ZEAMMB73_Zm00001d018627 | TGCGGAGGAGAGATAGGGAT | CTCCTGCTTCTTCCGGTACA | 59.22 | 59.1 | 55 | 55 | 224 |
| 17 | ZEAMMB73_Zm00001d009084 | TATATCACGTCCTGGTCCGC | GACCTGACCTTGCCAATCCT | 59.04 | 59.67 | 55 | 55 | 161 |
| 18 | 100286323 | GTGGGGATGAAGGGGAGATG | AAAACCAGATCCTCGCATGC | 59.52 | 58.9 | 60 | 50 | 187 |
| 19 | 100283248 | GCAGCATATCGTCGTTAGCC | GCGAGCGAGACAAATCCAAT | 59.22 | 58.99 | 55 | 50 | 178 |
| 20 | MSRB3 | ACGGGTGAGTACGACAAGTT | CGTCTTAAACCCCTCCCCTT | 58.96 | 59.01 | 50 | 55 | 237 |
| 21 | 100501403 | GAGATCATCGACGAGAGCCA | TGAGCATCCAGTCCACCAAT | 59.05 | 59.01 | 55 | 50 | 239 |
| 22 | 100382079 | CGTCAAGATACCACAGCTGC | CAGGATGGAGTGGTCGTAGG | 59 | 59.25 | 55 | 60 | 217 |
| 23 | 100280198 | ATTGCGGAGAACAAGGAGGA | GTTCTCCACAGCCTTCTTGC | 59.02 | 59.12 | 50 | 55 | 234 |
| 24 | ZEAMMB73_Zm00001d029457 | CACAAACACCCACCACTCTG | CCTCAACGCTGCTGATCTTC | 58.98 | 58.99 | 55 | 55 | 172 |
| 25 | 103627523 | AATGGACCGGCTTTGAACAC | TGAGCATGCGAGATTGAAGC | 59.04 | 58.99 | 50 | 50 | 250 |
| 26 | ZEAMMB73_Zm00001d049059 | AGAAGTTCATCACGCACAGC | GGAGCGCCTTCAACTTTTCA | 58.85 | 59.05 | 50 | 50 | 182 |
| 27 | 100192719 zea mays | AAGAACAAGTATGGCGACGC | CATCAGAGTCAGAGCGCAAC | 58.92 | 59 | 50 | 55 | 173 |
| 28 | ZEAMMB73_Zm00001d021351 | CGTTTTGACCGAGCAGCATT | TTGGGTCTGCACTCTTCTGT | 59.76 | 58.87 | 50 | 50 | 190 |
| 29 | 100216750 | TGCACTGTCTCTCTCTGTCC | ACCGGTGTTTCCCAGATTGA | 58.75 | 59.23 | 50 | 55 | 183 |
| 30 | ZEAMMB73_Zm00001d043620 | GTTGTACGTGGATGTGACCG | GAATCCGAGCAAAGTCCGTC | 58.93 | 59 | 55 | 55 | 237 |

**Notes:**

1. Primers were designed using Primer Premier 5 Designer software.
2. Genes/DAPs 1-11 were derived from TD-TC specific DAPs (labelled **Area I** in Figure 3 of the manuscript); Genes 12-13 are from (**Area III**); Genes 14-17 are from the overlapping DAPs between TD_TC and SD_SC (labelled **Area IV**); Genes 18-30 represents DAPs derived from Area **II (**specific DAPs of SD_TD).
3. Description of groups (Reference to Figure 3 and Tables 2-5) from which key drought responsive DAPs were derived:

**Area I** represents specific DAPs of TD_TC, that is, the specific drought responsive DAPs of the drought tolerant line YE8112.

**Area II** represents specific DAPs of SD_TD, that is, specific DAPs shared between the drought sensitive and drought tolerant lines after drought treatment.

**Area III** represents the 3 specifically shared DAPs between TD_TC and SD_TD, that is, drought responsive DAPs of the tolerant line that were also differentially expressed between the tolerant and sensitive lines after drought treatment.

**Area IV** represents the 5 DAPs shared by TD_TC and SD_SC, that is, the common (overlapping) drought responsive DAPs within line.
