# Supplementary figures and images for: Comparative Proteomic and Physiological Analyses of Two Divergent Maize Inbred Lines Provide More Insights into Drought-Stress Tolerance Mechanisms
Source: Int J Mol Sci. 2018 Oct 18;19(10):3225. doi: 10.3390/ijms19103225 (PMC6213998; doi:10.3390/ijms19103225)

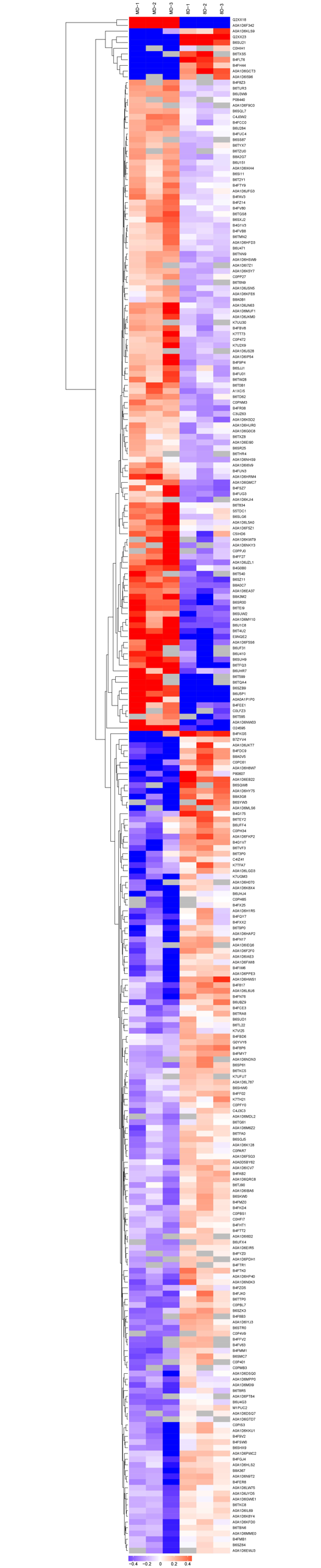

Supplement: Supplementary file 1 [file ijms-19-03225-s001.zip › Supplementary Material/Figure 4.jpg]
